# MIENTURNET: an interactive web tool for microRNA-target enrichment and network-based analysis

**DOI:** 10.1186/s12859-019-3105-x

**Published:** 2019-11-04

**Authors:** Valerio Licursi, Federica Conte, Giulia Fiscon, Paola Paci

**Affiliations:** 1 Institute for Systems Analysis and Computer Science “Antonio Ruberti”, National Research Council, Via dei Taurini 19, Rome, 00185 Italy; 2 Department of Biology and Biotechnology “Charles Darwin”, “Sapienza” University of Rome, Via dei Sardi 70, Rome, 00185 Italy

**Keywords:** Network analysis, miRNA regulatory network, Bioinformatics tool

## Abstract

**Background:**

miRNAs regulate the expression of several genes with one miRNA able to target multiple genes and with one gene able to be simultaneously targeted by more than one miRNA. Therefore, it has become indispensable to shorten the long list of miRNA-target interactions to put in the spotlight in order to gain insight into understanding the regulatory mechanism orchestrated by miRNAs in various cellular processes. A reasonable solution is certainly to prioritize miRNA-target interactions to maximize the effectiveness of the downstream analysis.

**Results:**

We propose a new and easy-to-use web tool MIENTURNET (MicroRNA ENrichment TURned NETwork) that receives in input a list of miRNAs or mRNAs and tackles the problem of prioritizing miRNA-target interactions by performing a statistical analysis followed by a fully featured network-based visualization and analysis. The statistics is used to assess the significance of an over-representation of miRNA-target interactions and then MIENTURNET filters based on the statistical significance associated with each miRNA-target interaction. In addition, the holistic approach of the network theory is used to infer possible evidences of miRNA regulation by capturing emergent properties of the miRNA-target regulatory network that would be not evident through a pairwise analysis of the individual components.

**Conclusion:**

MIENTURNET offers the possibility to consistently perform both statistical and network-based analyses by using only a single tool leading to a more effective prioritization of the miRNA-target interactions. This has the potential to avoid researchers without computational and informatics skills to navigate multiple websites and thus to independently investigate miRNA activity in every cellular process of interest in an easy and at the same time exhaustive way thanks to the intuitive web interface. The web application along with a well-documented and comprehensive user guide are freely available at http://userver.bio.uniroma1.it/apps/mienturnet/ without any login requirement.

## Background

MicroRNAs (miRNAs) are small, endogenously-initiated non-coding RNAs of about 22 nucleotides that post-transcriptionally control gene expression via either translation inhibition or degradation of their target mRNAs [1, 2]. The significance of miRNAs had been long overlooked until their initial discovery in the worm Caenorhabditis elegans [3] and successively in plants, animals, and even viruses [2]. Nowadays, it is becoming more and more evident that miRNAs are playing significant roles in regulatory mechanisms operating in various organisms, including developmental timing, cell differentiation, proliferation, apoptosis as well as tumorigenesis [4, 5]. Consistently, the identification and understanding of interactions between miRNAs and their targets is the foremost aim for deciphering the regulatory mechanisms that control the biogenesis and functionality of miRNAs in various cellular processes.

It is now widely known that the interactions between miRNA and mRNA in animals are usually restricted to the “seed” sequence near the 5’ terminus (i.e. nucleotides 2-7 at the 5’-end of the mature miRNA sequence). In particular, miRNAs control the target expression by base pairing to sequence motifs in the 3’ UTR of mRNAs with perfect or near perfect complementarities [6]. Conversely, most plant miRNAs regulate their targets based on a complete sequence complementarity [7]. However, there are still enigmas to be uncovered about principles governing miRNA-mRNA interactions and the nature of miRNA modulation.

In the last few decades, many miRNA-related bioinformatic tools have been established in order to predict candidate mRNAs based on the information from sequence, structure associated free energy and evolutionary conservation [8, 9]. Those bioinformatic methods usually result in the prediction of tens or hundreds of targets for each miRNA with high false positive rates [10]. Therefore, further experiments are still needed to determine how many of these predicted targets are genuinely targeted by miRNAs. Unfortunately, this utterly important task is hampered by the impossibility to experimentally validate all candidate genes individually that would be too laborious, time-consuming, and cost-inefficient.

A reasonable solution to the problem of the identification of effective miRNA-target interactions is certainly to prioritize them as to maximize the efficiency of the downstream validation experiments. More precisely, the principle is to identify the most promising candidate interactions and filter out the ones that appear of limited relevance, and then to investigate these promising candidate interactions more thoroughly.

Several computational methods have been proposed to tackle this prioritization problem and they have been used in practice to derive hypotheses that can be validated experimentally. Among the others, TargetScan predicts biological targets of miRNAs by searching for the exact matching between the seed region of a miRNA and the 3’ UTR of its targets and prioritizes miRNA-target interactions based on the predicted efficacy of targeting and also, as an option, based on the probability of conserved targeting [11].

More recently, new web-based applications such as miTEA [12], GSEA [13]^1^ and miEAA [14] have been developed to assess the statistical significance of an over-representation of miRNA-target interactions (i.e. miRNA-target enrichment analysis), and then filter them based on the resulting *p*-values.

Other web tools, like miRNet [15] and miRTargetLink [16], use instead network-based visualization methods to prioritize miRNA-target interactions and then filter by looking for those miRNAs linked simultaneously to multiple genes of interest.

Altogether these resources can help to prioritize candidate miRNA-target interactions, but researchers need to navigate multiple websites and then merge the results for further analysis. Thus, despite integrating information from different databases can be useful to cut down the number of miRNA-target interactions to be experimentally validated, this procedure requires general familiarity with the contents and structure of different databases as well as with their query languages. Moreover, information retrieval from distributed heterogeneous data sources still remains a challenging issue since data are stored in different ways and described by various formats, and this entails a semantic heterogeneity that includes problems on naming such as with synonyms, that is same concept expressed with different terms. For example, GSEA provides the results of miRNA-target enrichment analysis reporting the miRNA family name according its own standard name (e.g. ACATTCC_MIR1_MIR206), whereas miRNet requires as input miRNA identifiers from miRBase (e.g. ID: hsa-miR-206, or ACCESSION: MIMAT0000462). Thus, the output of GSEA could not be directly processed by miRNet but needs of a not always trivial integration coding.

Here, we propose a new web tool called MIENTURNET (MicroRNA ENrichment TURned NETwork) that tackles the problem of prioritizing miRNA-target interactions, both computationally predicted and experimentally validated, and then it filters based on the statistical significance resulting from a miRNA-target enrichment analysis. Finally, MIENTURNET makes use of the holistic approach of the network theory to infer possible evidences (computational or experimental) of miRNA regulation by capturing topological properties of the miRNA-target regulatory network that would be not evident through a pairwise analysis of the individual components. Therefore, MIENTURNET offers the possibility to consistently perform all the above-mentioned analyses by using only a single tool and is especially meant for non-expert users thanks to the simple and intuitive web interface, to the delivery of an exhaustive and well-documented set of output information, and to the practical user guide provided as Additional file 1. MIENTURNET fetches data of computationally predicted and experimentally validated miRNA-target interactions only from TargetScan and miRTarBase, respectively. It is noteworthy that TargetScan appears as the most up-to-date tool for sequence-based miRNA-target predictions [8], whereas miRTarBase appears as the most up-to-date tool for validated interactions, and it offers an easy downloadable spreadsheet [17].

## Implementation

MIENTURNET web tool was implemented by using the R programming language (Release 3.4.4, March 2018) for statistical computing and graphics (http://www.rproject.org/). The whole web framework was developed based on the *shiny* package (version 1.2) from RStudio (http://shiny.rstudio.com). Indeed, *shiny* is a free, open source, extensible package that allows to create an interactive web interface for sharing analysis and graphics from R. The performance of *shiny* package was widely tested and validated by several successful web applications [18–21]. Additional R packages used to create MIENTURNET include: *visNetwork* (ver. 2.0.4), *igraph* (ver. 1.2.2) [22], *shinyWidgets* (ver. 0.4.4), *shinyBS* (ver. 0.61) and *clusterProfiler* (ver. 3.8.1) [23].

### Data retrieval

#### Simulated data

We generated simulated expression profiles for 100 samples spanning 12440 genes that are available in Targetscan (Release 7.2) [11] and for 100 samples spanning 14888 genes that are available in miRTarBase (Release 7.0, September 2017) [24]. In each simulated dataset *E*, the expression level of each gene *g* in each sample *s* was independently randomly drawn from a standard normal distribution, *E*(*g*,*s*)∼*N*(0,1), and 10 random miRNAs were simulated to be active at different levels of influence: the first repressing its top 100 targets, the second repressing its top 200 targets, the third repressing its top 300 targets, and so on until the last repressing its top 1000 targets. In addition, for each simulated data set, the level of miRNAs activity *α* is increased from 0.3 to 1 with 0.05 steps. The miRNA repression was simulated by reducing the expression levels of the miRNA targets for 50 of the 100 samples. The reduction level is set equal to (*α* + *ε*) with *ε* drawn from a standard normal distribution. This means that the expression value for a target *g* of an active miRNA in an affected sample *s* is given by *E*(*g*,*s*)=*N*(0,1)−(*α*+*N*(0,1)).

#### Expression profiling

miRNA expression profiles from six different human tissue types were obtained from DASHR 2.0 [25], which is a comprehensive database of human small non-coding RNA genes and mature products. Repeat measurements of the same tissue were averaged resulting in one profile for each tissue type, and miRNAs whose expression levels were greater than the 75^*th*^ percentile of the tissue type distribution were selected as the most tissue-representative miRNAs. Protein expression levels from the same human tissue types based on immunohistochemistry using tissue microarrays were downloaded from the Human Protein Atlas version 18.1 [26]. For each tissue type, proteins with high expression levels in that tissues were selected as the most tissue-representative proteins. Note that both definitions of the most tissue-representative miRNAs and proteins ensure that they are highly expressed *at least* in a certain tissue, but it does not require they are expressed *only* in that tissue.

#### Positive predictive value

To test the performance of MIENTURNET in capturing the most tissue-representative miRNAs, we computed the positive predictive value (PPV) for different tissues, which is defined as [27]:

$$\begin{aligned} PPV = \frac{\text{number of true positives}}{(\text{number of false positives} + \text{number of true positives})} \end{aligned} $$

where a “true positive” is an outcome where the model *correctly* predicts the positive class, whereas a “false positive” is an outcome where the model *incorrectly* predicts the positive class. In our analysis, PPV represents the number of the most tissue-representative miRNAs on the total number of miRNAs identified by MIENTURNET targeting an input list of the most tissue-representative proteins. The ideal value of the PPV in a perfect test is 1 (100%), and the worst possible value would be zero. The PPV statistic is often called “precision” and a small positive predictive value (e.g. PPV < 50%) indicates that more than half of the positive results from the testing procedure are false positives.

#### miRNA-target interactions

The predictions of miRNA targets and the information about the miRNA family members with their seed were downloaded from TargetScan web site (Release 7.2, March 2018) [11]. The experimentally validated miRNA-target interactions were downloaded from miRTarBase web site (Release 7.0, September 2017) [24].

Currently, MIENTURNET supports the choice of six organisms shared from TargetScan and miRTarBase, that is: Human (*Homo sapiens*), Mouse (*Mus musculus*), Rat (*Rattus norvegicus*), Worm (*Caenorhabditis elegans*), Fruit fly (*Drosophila melanogaster*), Zebrafish (*Danio rerio*).

All miRNA entries are annotated according to the latest miRBase database (Release 22, March 2018) [28], while all mRNA entries are annotated according to the latest NCBI database (Release 227, August 2018) [29].

### Tool description

**Fig. 1:**
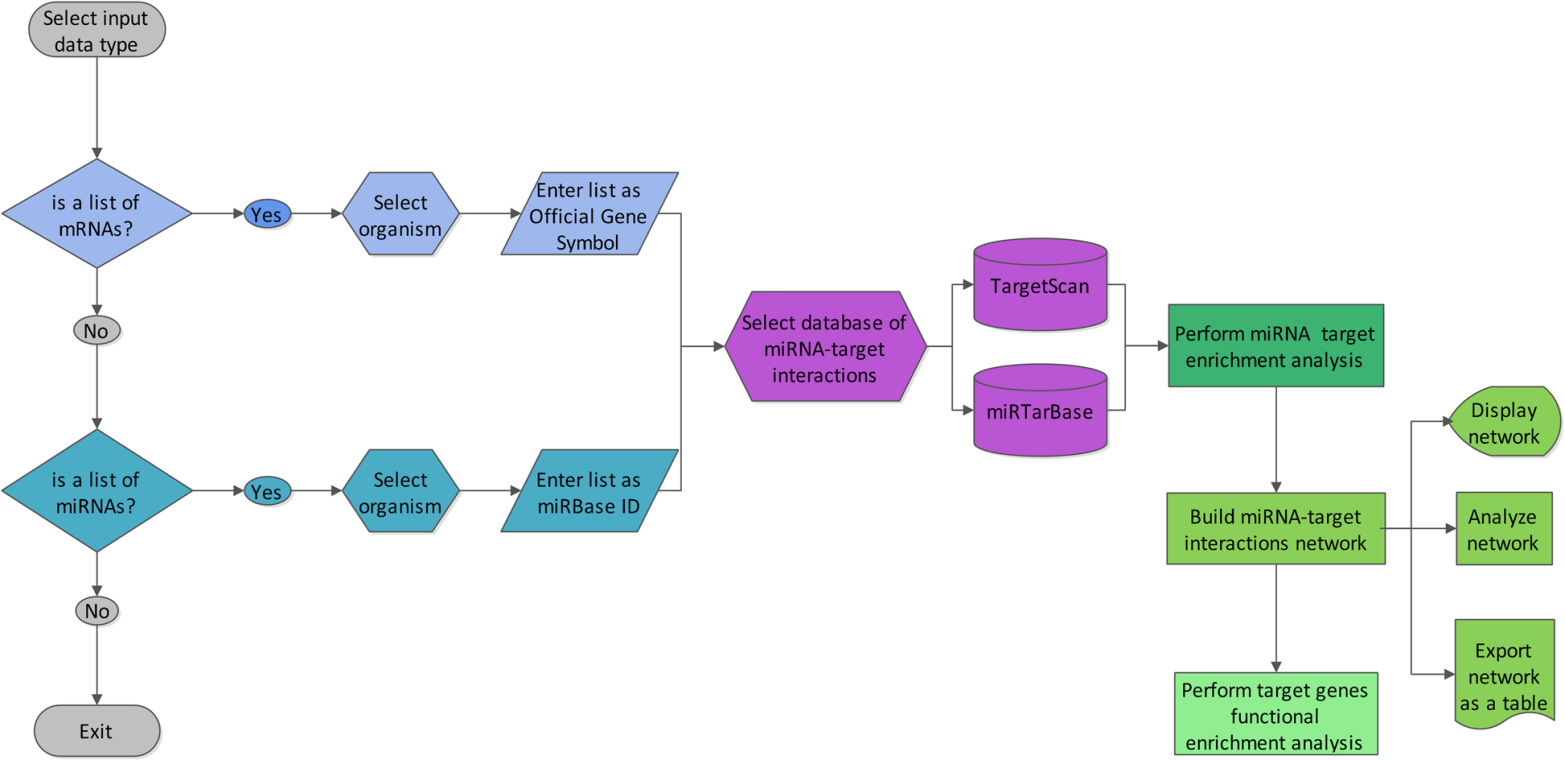
Flowchart of MIENTURNET web tool
- receiving in input a list of mature miRNAs according to miRBase ID (e.g. hsa-miR-15a-5p) and inferring possible evidences (computational or experimental) of their regulation on target genes based on a statistical analysis for over-representation of miRNA-target interactions.

The flowchart of MIENTURNET is shown in Fig. 1. MIENTURNET is devised for:

- receiving in input a list of genes according to Official Gene Symbol (e.g. PTEN for *human* species, Pten for *mouse* species) and inferring possible evidences (computational or experimental) of miRNA regulation based on a statistical analysis for over-representation of miRNA-target interactions;

The resulting miRNA-target interactions are visualized as a network and then analyzed according their topological features.

**Fig. 2:**
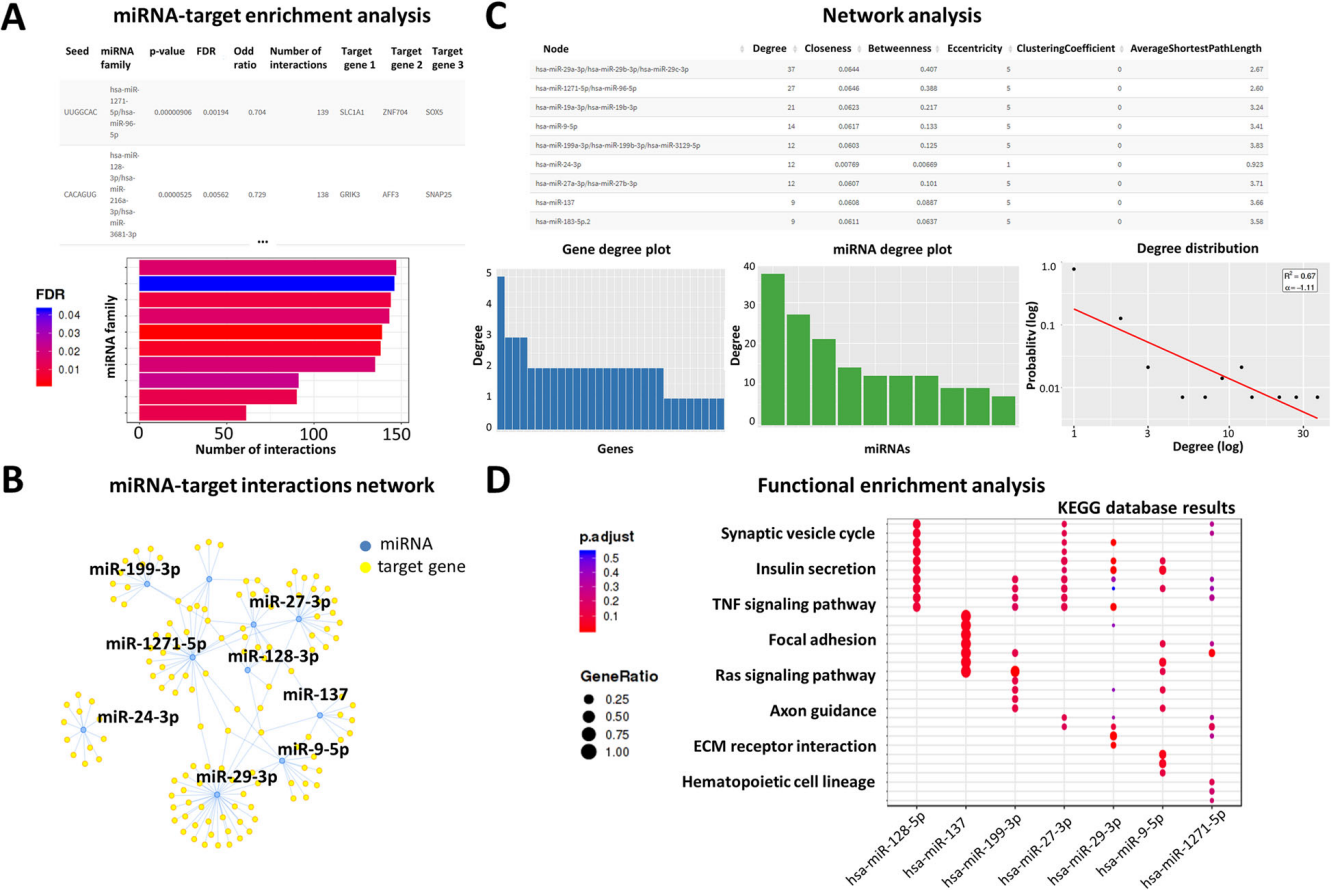
Outputs of MIENTURNET web tool with the example list of genes as input. **a** Table of results from miRNA-target enrichment analysis (top); bar plot representing each miRNA resulting from the enrichment along with the number of its target genes (bottom). The color of the bars represent the adjusted *p*-values (FDR). **b** Visualization of miRNA-target interaction network where blue circles refer to miRNAs, while yellow circles refer to their target genes. **c** Table of network topological properties (top); network degree plots for target genes (bottom-left) and for miRNAs (bottom-middle); and nodes degree distribution shown on double logarithmic axis (log-log plot), in which the straight line corresponds to the power-law fit (bottom-right). **d** Dot plot of functional enrichment analysis for target genes of selected miRNAs resulting from the enrichment analysis. The Y-axis reports the annotation categories (e.g. KEGG pathways) and the X-axis reports the selected miRNAs. The color of the dots represent the adjusted *p*-values (FDR), whereas the size of the dots represents gene ratio (i.e. the number of miRNA targets found enriched in each category over the number of total genes associated to that category)

MIENTURNET performs a miRNA-target enrichment analysis (Fig. 2a) by calculating the following statistics (i.e. *p*-value resulting from the hypergeometric test):

$$p = 1- \sum_{i=0}^{X-1} \frac{\binom{K}{i}\binom{M-K}{N-i}}{\binom{M}{N}} $$

where *M* is the dimension of the universe, that is the number of all predicted (validated) miRNA-target interactions encompassed in TargetScan (miRTarBase); *N* is the length of the input list; *K* is the number of predicted (validated) miRNA-target interactions encompassed in TargetScan (miRTarBase) for a selected gene or miRNA according to the type of the input list; *X* is the number of predicted (validated) miRNA-target interactions encompassed in the input list for the selected gene or miRNA.

MIENTURNET allows to visualize the resulting miRNA-target interactions as a network that can be filtered, explored, and customized interactively in order to improve its visualization and understanding (Fig. 2b). For example, by choosing miRTarBase database, the results can be filtered according to the type of evidence categories used by miRTarBase to validate the miRNA-target interactions: ‘Strong’ for considering strong experimental methods (e.g., Luciferase assay, Western); ‘Weak’ for considering weaker experimental evidence (e.g., CLIP); ‘Strong and Weak’ for considering both strong and weak experimental methods. In addition, MIENTURNET computes the topological properties for each node in the miRNA-target interaction network (i.e. degree, betweenness, average shortest path length, eccentricity, clustering coefficient) in order to find nodes displaying a central role (Fig. 2c). Then, it estimates the nodes degree distribution (i.e. the probability distribution of degrees over the whole network) along with the power-law fit, in order to determine whether the network exhibits a scale-free behaviour (Fig. 2c), consistent with how emerged so far in almost all biological networks [30–32]. MIENTURNET offers also the possibility to perform a functional enrichment analysis of the targets of selected miRNAs (Fig. 2d), in order to gain insight into understanding the biological processes underlying the target gene activity. For this analysis, currently the choice is among the following annotation databases: KEGG pathways [33], Reactome [34], WikiPathways [35] and Disease Ontology [36] (only with Homo sapiens).

MIENTURNET reports numerous output files containing the results of its analyses (i.e. miRNA-target enrichment analysis, selected miRNA-target functional enrichment analysis and network analysis). These files are simple tabular output files that can be viewed with any spreadsheet application (such as Microsoft Excel). However, browsing these files by eye is not especially easy, and working with data across multiple files can be quite difficult and could require nontrivial scripts. MIENTURNET drastically simplifies data exploration task by creating, for each of the performed analyses, publication-ready plots.

## Results and discussion

### Performance evaluation

**Fig. 3:**
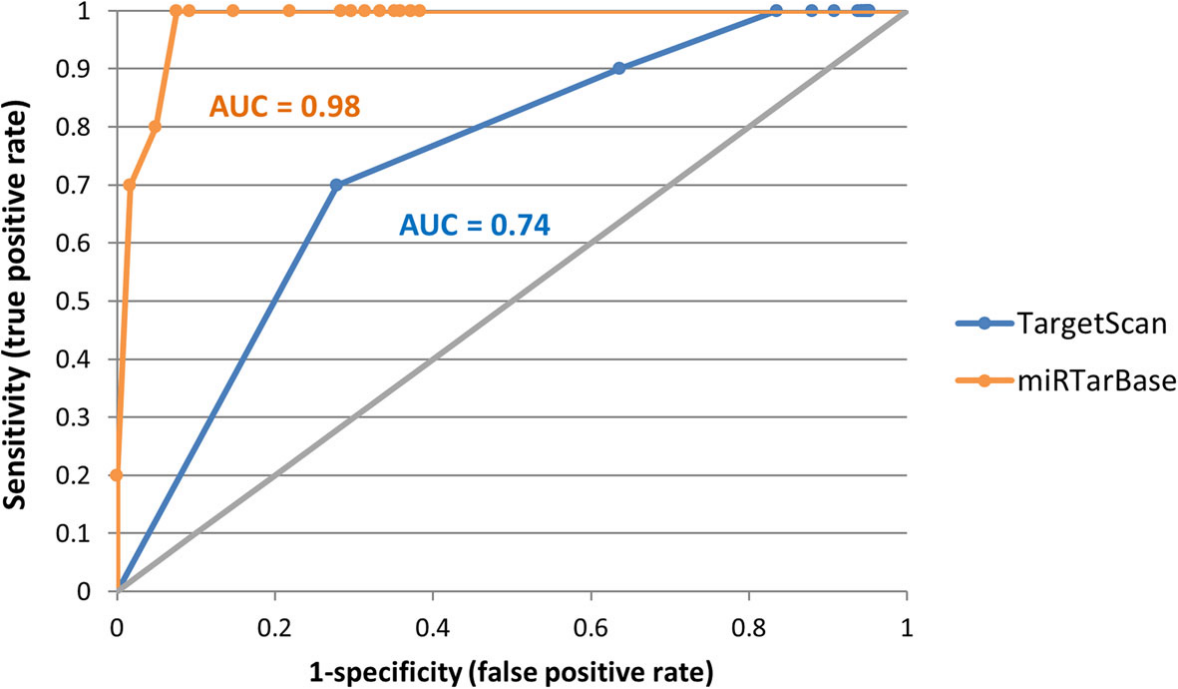
Performance of MIENTURNET in detecting miRNA activity. ROC curves resulting by the MIENTURNET application on simulated data by considering both predicted interactions from TargetScan (blue curve) and validated interactions from miRTarBase (orange curve). The data simulated the activity of 10 miRNAs at different levels (*α*∈ [0.3-1] with 0.05 steps). For each level of *α*, we computed the true positive rate (i.e. sensitivity) placed on Y-axis, and the false positive rate (i.e. 1 - specificity) placed on X-axis. Sensitivity is the rate of truly active miRNAs identified by MIENTURNET on the total number of active miRNAs; specificity is the rate of truly inactive miRNAs identified by MIENTURNET on the total number of inactive miRNAs. We run MIENTURNET under default parameters. Diagonal grey line represents the line of no-discrimination

To evaluate the performance of MIENTURNET, we tested it on simulated dataset designed to model the activity of a small set of miRNAs in a subset of samples (see “Implementation” section for further details). We analyzed the performance of MIENTURNET for different levels of miRNA activity by using Receiver Operator Characteristic (ROC) curve both for computationally predicted and experimentally validated miRNA-target interactions. We found that MIENTURNET is able to detect active miRNAs with high level of sensitivity and specificity even in cases of low activity, both considering miRTarBase (AUC = 98%) and TargetScan (AUC = 74%) as reference database (Fig. 3).

**Fig. 4:**
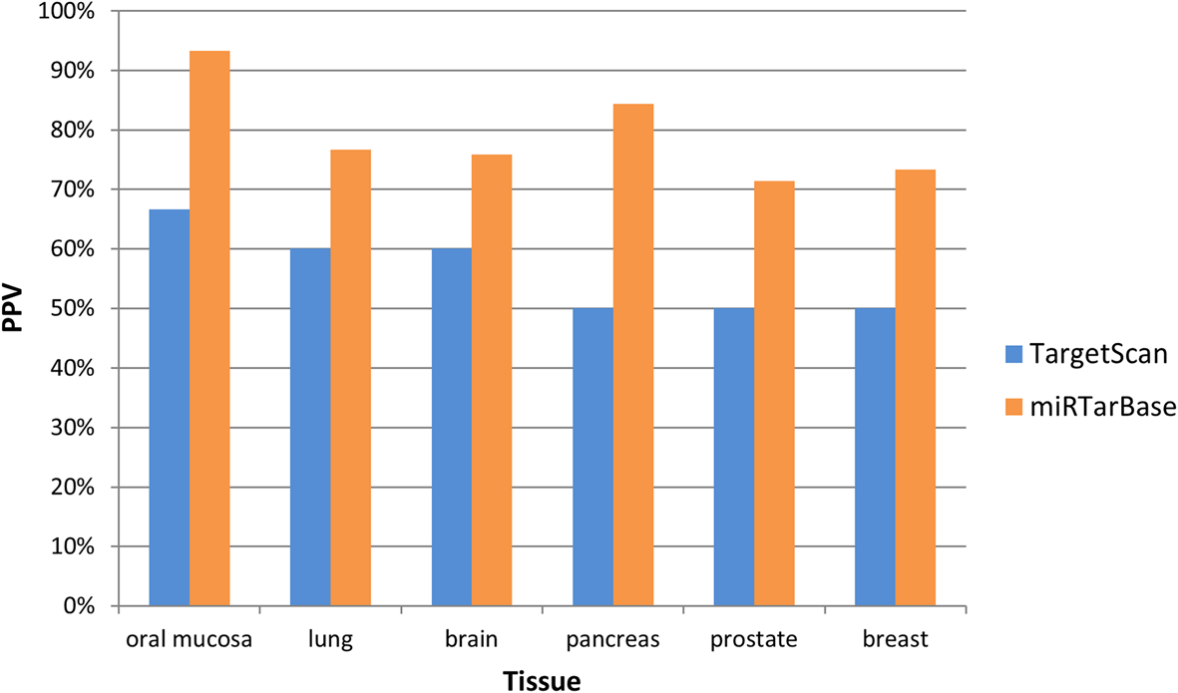
Performance of MIENTURNET in detecting the most tissue-representative miRNA activity. Bar plot of the positive predictive value (PPV) computed by considering predicted interactions from TargetScan (blue bars) and validated interactions from miRTarBase (orange bars) along with each tissues type. PPV is the number of the most tissue-representative miRNAs on the total number of miRNAs identified by MIENTURNET targeting an input list of the most tissue-representative proteins. We run MIENTURNET under default parameters

To better assess the precision of MIENTURNET in detecting miRNA activity, we tested it on a more practical context of less controlled dataset, publicly available both for miRNA and protein expression profiles in human healthy tissue types (see “Implementation” section for further details). We analyzed the performance of MIENTURNET in different tissues, where miRNA and protein expression profiles were both available, by computing the positive predictive value (PPV) both for computationally predicted and experimentally validated miRNA-target interactions. The rational behind this analysis was to give as input list to MIENTURNET the most tissue-representative proteins and evaluate the precision of MIENTURNET in capturing the most tissue-representative miRNAs targeting them. We found that MIENTURNET is able to detect the most tissue-representative interactions with an high precision, both considering miRTarBase (PPV > 70%) and TargetScan (PPV > 50%) as reference database (Fig. 4).

### Comparing MIENTURNET to other existing tools

As we stated above, the last few years have witnessed the increasing of web-based applications for supporting the identification of miRNA-target interactions that could be involved in various crucial cell processes or developmentally important cellular functions. Among them, the most used are miTEA [12], GSEA [13], miEAA [14], miRNet [15], and miRTargetLink [16]. A comprehensive comparison of these tools is given in Table 1.

**Table 1:** Comparison of MIENTURNET with other web tools developed for the identification and analysis of miRNA-target interactions (MTIs)

Tools	miTEA	GSEA/MSigDB	miEAA	miRNet	miRTargetLink	MIENTURNET
Reference	[12]	[13]	[14]	[15]	[16]	
Last Release	February 2013	July 2018	April 2016	March 2018	April 2016	March 2019
Input list						
Gene identifiers	Gene symbol, RefSeq, Uniprot, Unigene, Ensembl	Gene symbol, Entrez	-	Gene symbol, Ensembl, Entrez	Gene symbol	Gene symbol
miRNA identifiers ^∗^	-	-	ID	ID, Accession	ID	ID
Others	-	-	-	✓	-	-
Queries						
Multiple item	✓	✓	✓	✓	✓	✓
Organisms						
Species ^∗∗^	Human, Mouse, Rat, Zebrafish, Fruit fly	Human	Human	Human, Mouse, Rat, Cattle, Chicken, Zebrafish, Fruit fly, Worm, Helminth	Human	Human, Mouse, Rat, Zebrafish, Fruit fly, Worm
Species #	5	1	1	9	1	6
Reference database						
Predicted target genes	TargetScan, MicroCosm, EIMMo	MSigDB	-	miRanda ^∗∗∗^	miRanda	TargetScan
Experimental target genes	-	-	miRTarBase	TarBase, miRTarBase, miRecords	miRTarBase, in-house data	miRTarBase
Others	-	-	✓	✓	-	-
Statistical analysis						
Differential expression	-	-	-	✓	-	-
Functional enrichment	-	✓	✓	✓	✓	✓
miRNA-target enrichment	✓	✓	✓	-	-	✓
MTI network						
Visualization	-	-	-	✓	✓	✓
Customization	-	-	-	✓	✓	✓
Filtering	-	-	-	✓	✓	✓
MTI network analysis						
Degree	-	-	-	✓	-	✓
Betweenness	-	-	-	✓	-	✓
Closeness	-	-	-	-	-	✓
Average shortest path	-	-	-	-	-	✓
Eccentricity	-	-	-	-	-	✓
Clustering coefficient	-	-	-	-	-	✓
Fit power low	-	-	-	-	-	✓

^*^miRNA identifiers from miRBase database [28]

^**^species common name (*scientific name*): Human (*Homo sapiens*), Mouse (*Mus musculus*), Rat (*Rattus norvegicus*), Cattle (*Bos taurus*), Chicken (*Gallus gallus*), Zebrafish (*Danio rerio*), Fruit fly (*Drosophila melanogaster*), Worm (*Caenorhabditis elegans*), Helminth (*Schistosoma mansoni*)

^***^only for Cattle, Chicken, Helminth

Almost all these tools look for miRNAs targeting an input list of candidate genes by exploiting information on miRNA-target interactions computationally predicted and/or experimentally validated, and allow to perform functional enrichment analysis of the target genes. Exceptions are miEAA being tailored *only* for miRNA input, while miRNet and miRTargetLink being tailored *also* for miRNA input.

The prioritization problem of miRNA-target candidate genes is faced by all these tools, but each of them makes use of a different approach to deal with it. In particular, miTEA, GSEA and miEAA perform a statistical analysis to evaluate possible miRNA-target enrichment in the input list by exploiting different methodologies: miTEA takes advantage of a novel non-parametric hypothesis test, called minimum-mHG, requiring a previously ranked list of genes [12]; GSEA and miEAA implement an over-representation analysis based on the hypergeometric statistic test [13, 14]. However, miEAA takes into account only validated miRNA-target interactions from miRTarbase, whereas miTEA and GSEA explore only predicted miRNA-target interactions from different databases: miTEA from TargetScan and, as an option, from MicroCosm and EIMMo; GSEA from its internal Molecular Signatures Database (MSigDB), which contains targets of only 221 human miRNAs cataloged in an outdated version of miRBase.

Conversely, miRTargetLink and miRNet assist researchers in understanding miRNAs and their targets through a network-based visualization method and offer the possibility to consider computationally predicted or experimentally validated miRNA-target interactions, both selecting as reference databases miRanda and miRTarBase, respectively. It is worthy mentioning that miRanda web server is not maintained anymore and the standalone version is updated to 2010. Additionally, miRNet collects experimentally validated miRNA-target interaction data also from TarBase and miRecords. However, the current version of TarBase is not downloadable and the previous one requires a prior consent, whereas last update of miRecords back to 2013.

MIENTURNET was designed to exploit the strengths of the above-mentioned tools and to overcome some of their limitations. Indeed, MIENTURNET tackles the problem of prioritizing miRNA-target interactions by using a statistical analysis together with a network-based visualization and analysis. It is able to accept as input a list of genes as well as a list of miRNAs searching for both predicted and experimentally validated miRNA-target interactions from the most reliable and updated databases, TargetScan and miRTarBase, respectively. MIENTURNET offers also the possibility to perform functional enrichment analysis of target genes of individual miRNAs by querying widespread annotation databases (i.e. KEGG, Reactome, WikiPathways).

### Future perspectives

Currently, MIENTURNET supports the choice of six organisms (Table 1) for which both predicted and experimentally validated miRNA-target interactions were available. In future, we would like to include other organisms, like plants, when well-structured, easy downloadable and queryable databases will be available for both predicted and validated miRNA-target interactions.

Moreover, we plan to integrate the two miRNA regulatory networks obtained by considering both predicted and experimentally validated miRNA-target interactions and prioritize them according to the guilt-by-association concept. In practice, this method should favor candidate genes that are linked simultaneously to multiple miRNAs sharing common features. For instance, if a candidate gene interacts with two miRNAs that are already known to be involved in the same phenotype and only one of these two interactions has been experimentally validated, the other appears promising for effective miRNA targeting. This can be extended by considering multiple sources of information beside miRNA-target interactions, which is often what prioritization methods do.

Finally, although MIENTURNET exploits the most up-to-date tool for sequence-based miRNA-target predictions (i.e. TargetScan) and the most up-to-date tool for validated interactions (i.e. miRTarBase), it could be likely that in the future both databases could no longer be updated. If this is the case, we will use the most up-to-date tools at that time.

## Conclusions

We developed MIENTURNET, a user-friendly web tool that integrates the miRNA-target enrichment analysis with a network-based visualization and analysis in order to tackle the prioritization problem of miRNA-target candidate interactions. Although the procedure implemented by MIENTURNET assumes basic skills of network theory and statistics, the tool does not require extensive bioinformatics expertise and is meant for novices and experts alike.

The protocol begins with an input list of miRNAs or mRNAs and produces a network of statistically significant miRNA-target interactions together with a fully-featured analysis of the topological properties of all nodes as well as a fully-featured analysis of the functional enriched categories in a specific set of nodes. The numerous output files containing the analysis results are tab-delimited text files that can be opened with spreadsheet programs such as Microsoft Excel. In addition, MIENTURNET creates publication-quality visualizations of the analysis results.

The strength of MIENTURNET is to enable researchers without computational and informatics skills to play with their data in an easy and at the same time exhaustive fashion and hence to stimulate miRNA-related research activities by investigating miRNA activity in every cellular process of interest. The weakness of MIENTURNET relies on the databases that were selected to be used. Despite being the most comprehensive and reliable databases in miRNomics field, they have some limitations: TargetScan is based on predicted data, which are not always confirmed by experimental methods, and miRTarBase does not contain all published data.

## Availability and requirements

**Project name**: MIENTURNET

**Project home page**: http://userver.bio.uniroma1.it/apps/mienturnet/

**Operating system(s)**: Windows, macOS, and Linux

**Programming language**: R programming language (Release 3.4.4, March 2018)

**Other requirements**: MIENTURNET was tested and found compatible with Chrome 70.0 and Firefox 62.0 on Ubuntu 16.04, MacOS 10.13.6 and Windows 10 as well as with Safari 12.0.2 and Internet Explorer 11

**License**: GNU GPL

**Any restrictions to use by non-academics**: MIENTURNET is free and open to all users and there is no login requirement

## Supplementary information

**Additional file 1** This file contains the user guide of MIENTURNET showing how the tool works and how to use it.

## Data Availability

The interactive web application along with a well-documented and comprehensive user guide are freely available at http://userver.bio.uniroma1.it/apps/mienturnet/. This website is free and open to all users and there is no login requirement.

## Abbreviations

AUC: Area Under Curve
FDR: False Discovery Rate
mHG: minimum-hypergeometric
MIENTURNET: MicroRNA ENrichment TURned NETwork
miRNAs: microRNAs
MTI: miRNA-target interaction
PPV: Positive Predictive Value
ROC: Receiver Operator Characteristic

## Footnotes

1 Hereafter, when citing GSEA we refer to the Investigate Gene Sets tool from GSEA/MSigDB web site.
