# Supplementary material for: MIENTURNET: an interactive web tool for microRNA-target enrichment and network-based analysis
Source: BMC Bioinformatics. 2019 Nov 4;20:545. doi: 10.1186/s12859-019-3105-x (PMC6829817; doi:10.1186/s12859-019-3105-x)
Supplement: Supplementary file 1 — Additional file 1 This file contains the user guide of MIENTURNET showing how the tool works and how to use it. [file 12859_2019_3105_MOESM1_ESM.pdf]

Home

MIENTURNET (MIcroRNAENrichmentTURnedNETwork) is an easy-to-use web tool devised for:

- receiving in input a list of genes and inferring possible evidences (computational or experimental) of miRNA regulation based on a statistical analysis for over-representation of miRNA-target interactions;
- receiving in input a list of mature miRNAs and inferring possible evidences (computational or experimental) of their regulation on target genes based on a statistical analysis for over-representation of miRNA-target interactions.

The resulting miRNA-target interactions are visualized as a network.

MIENTURNET makes use of computationally predicted or experimentally validated miRNA-target interactions downloaded from TargetScan [1] website Release 7.2 (March 2018) and miRTarBase [2] website Release 7.0 (September 2017), respectively (Figure 1).

Currently, MIENTURNET supports the choice of six organisms: Homo sapiens, Mus musculus, Rattus norvegicus, Caenorhabditis elegans, Drosophila melanogaster, Danio rerio.

MIENTURNET is especially meant for non-expert users through the development of a simple and intuitive web interface as well as the delivery of an exhaustive and well-documented set of output information (Figure 2).

Availability and requirements

MIENTURNET was created using the shiny package from RStudio (https://cran.r-project.org/web/packages/shiny), it has been developed by Valerio Licursi, Federica Conte, Giulia Fiscon and Paola Paci of Institute for Systems Analysis and Computer Science “Antonio Ruberti”, National Research Council, Rome, Italy. The interactive MIENTURNET web application is available at http://userver.bio.uniroma1.it/apps/mienturnet/. This website is free and open to all users and there is no login requirement.

Browser compatibility

OS	Version	Chrome	Firefox	Microsoft Edge	Internet Explorer	Safari
**Linux**	Ubuntu 16.04	70.0	62.0	n/a	n/a	n/a
**MacOS**	High Sierra 10.13.6	70.0	62.0	n/a	n/a	12.0.2
**Windows**	10	70.0	62.0	17.17134	11	n/a

How to cite MIENTURNET

If you use MIENTURNET, please cite the following article.

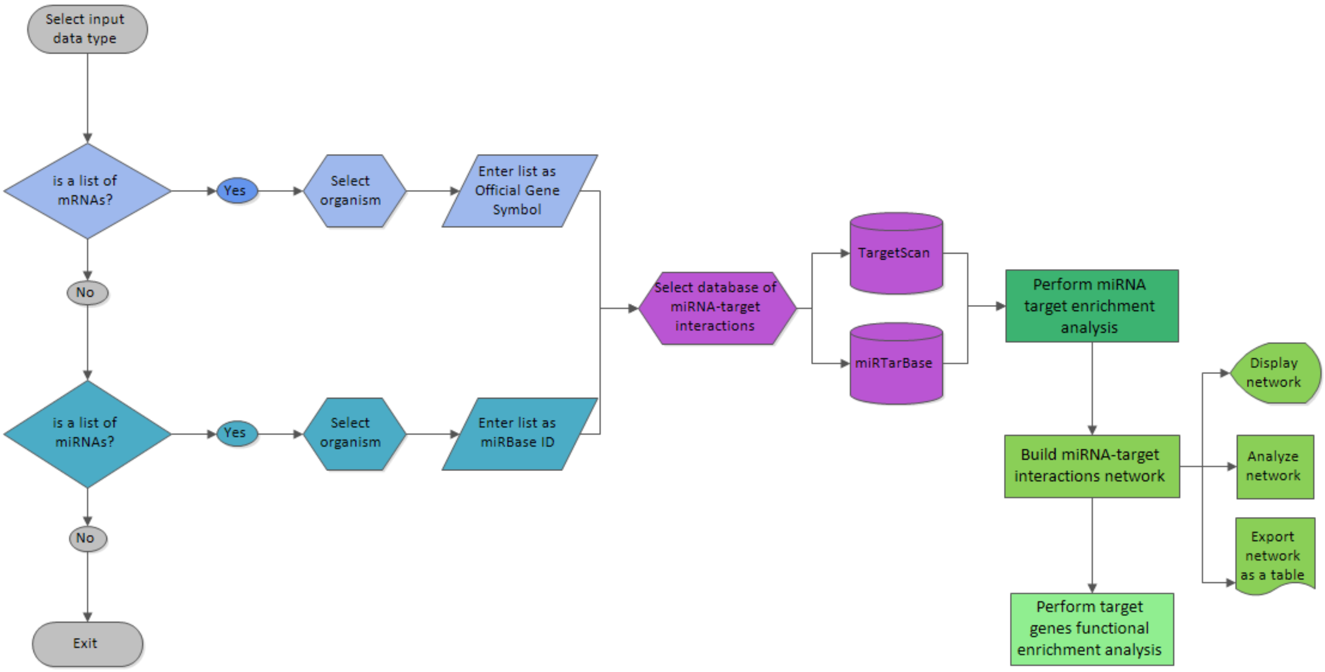

Figure 1. Flowchart of MIENTURNET web tool.

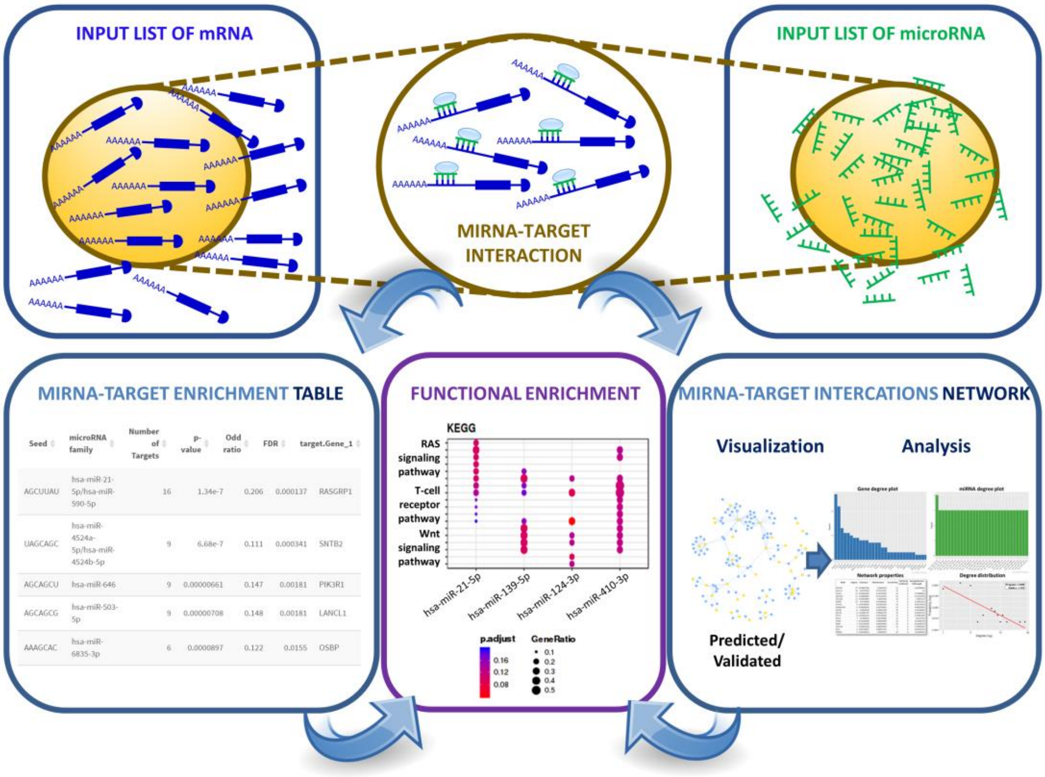

Figure 2. Sketch of MIENTURNET input/output files. MIENTURNET looks for miRNAs targeting an input list of mRNAs (top-left) or mRNAs targeted by an input list of miRNAs (top-right) and it performs a statistical analysis for over-representation of miRNA-target interactions (bottom-left) together with a network-based visualization and analysis of the resulting enriched interactions (bottom-right).

Tutorial

From the **Home** page of MIENTURNET, click on **Data Upload** link on the top navigation bar to start the analysis.

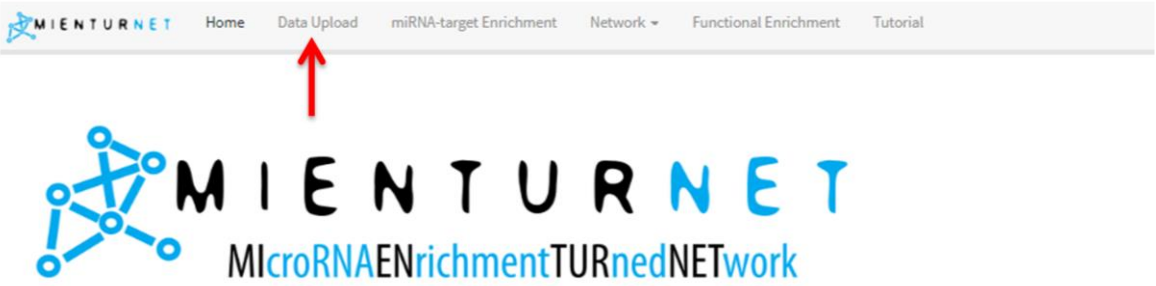

Entering a list of miRNAs

Data Upload link

If the user is interested to find targets of a given list of miRNAs, from the **Data Upload** page, select **miRBase ID** as type of the input list**.**

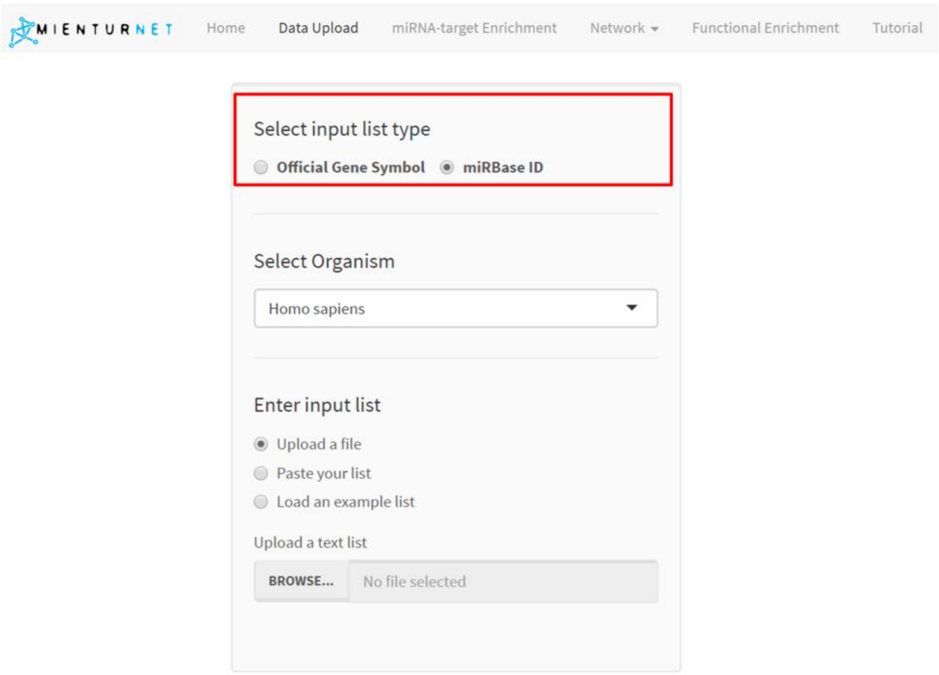

Select the organism from the drop-down menu.

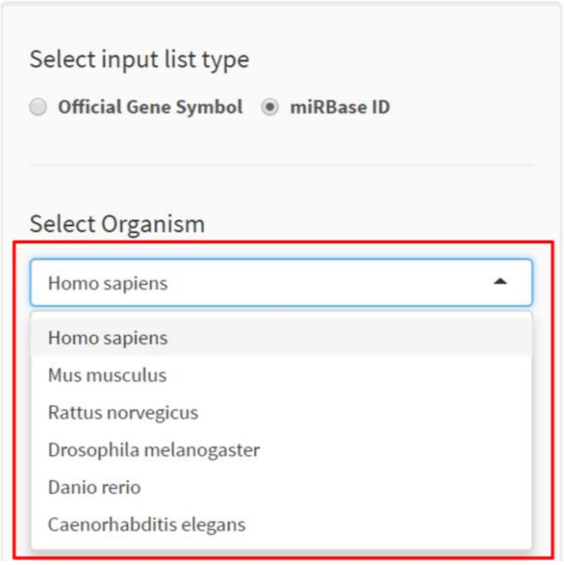

Enter the input list by using one of the following options:

1) **Upload a file** if the list is saved in a text file on the own computer and then click BROWSE to quickly access to the file

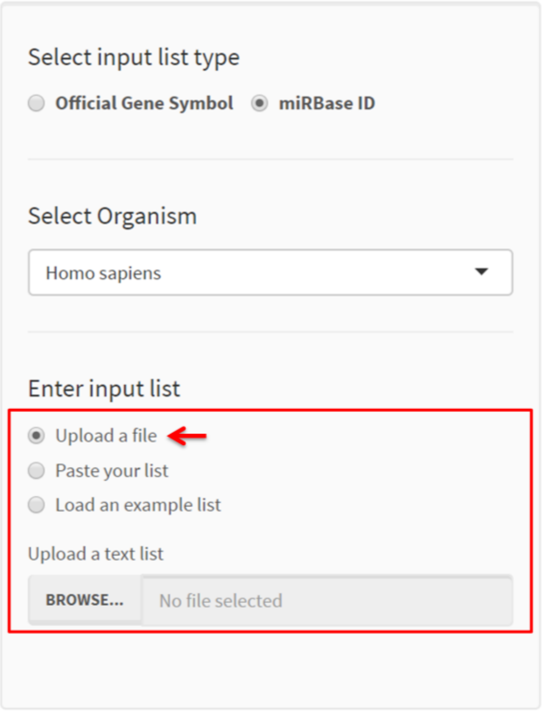

2) **Paste your list** in the specific text area and then click SUBMIT

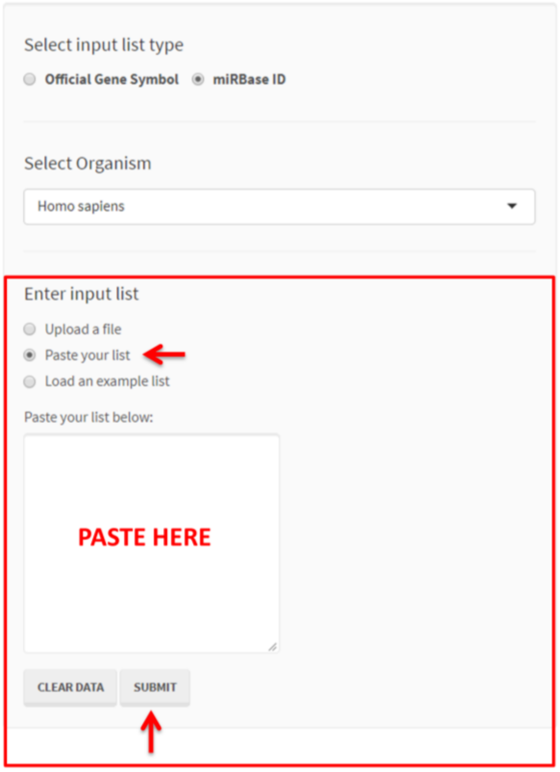

3) **Load an example list** for playing with data. With this choice, the user has the possibility to explore the functionality of MIENTURNET by exploiting a usage example.

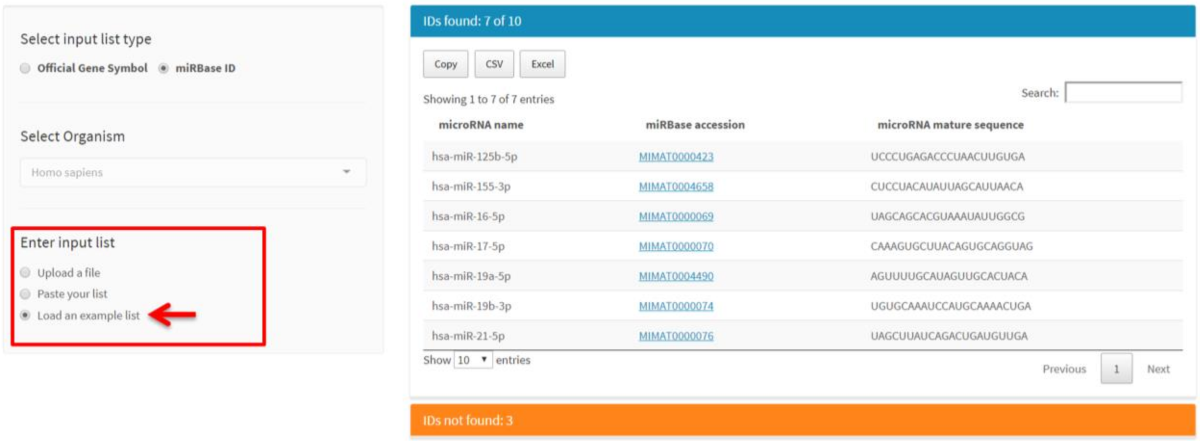

Hereafter, we will show how MIENTURNET works by loading the example input list of miRNAs selected by the authors. Note that this list represents a subset of known oncomiR.

Input list identifiers

The input list must include identifiers of the mature miRNAs as miRBase ID (e.g., hsa-miR-15a-5p). The tool processes the input file and immediately provides in the **IDs found** collapsible panel the total number of miRNAs recognized by miRBase database together with a table that includes: microRNA names, miRBase accession IDs as hyperlinks to miRBase, and microRNA mature sequences.

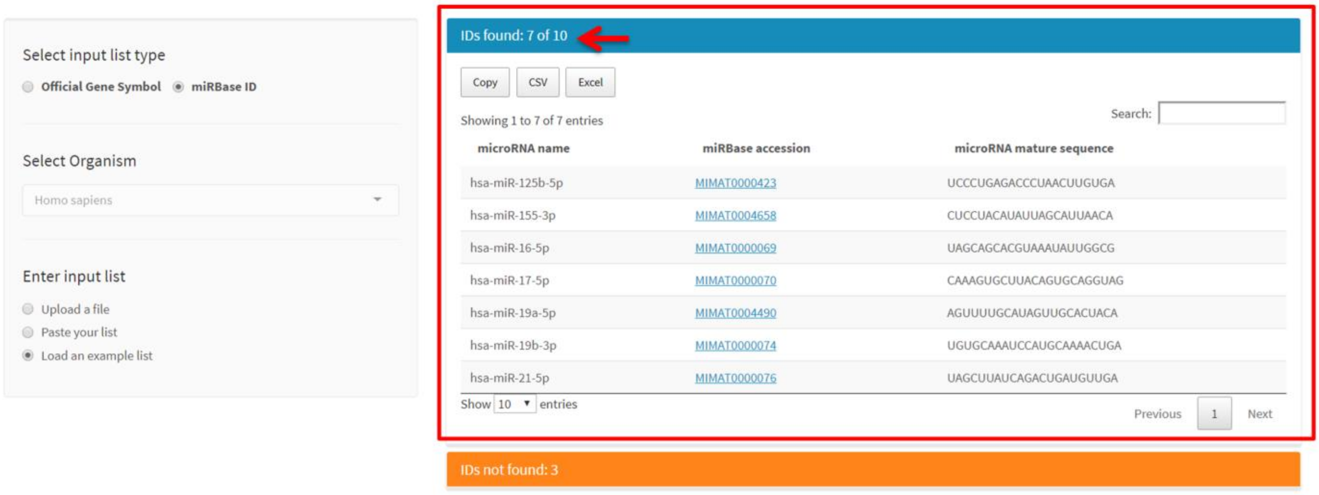

In the input list table, the user has also the possibility to:

i. visualize the first 10, 25, 50, 100 entries by clicking the drop-down menu at the bottom-left
ii. navigate the table trough the navigation controller buttons at the bottom-right
iii. search for a specific miRNA in the input list by typing the name in the search bar at the top-right.
iv. copy the table to the clipboard, export it in a comma separated format (CSV) or in EXCEL format through the dedicated buttons placed at the top-left.

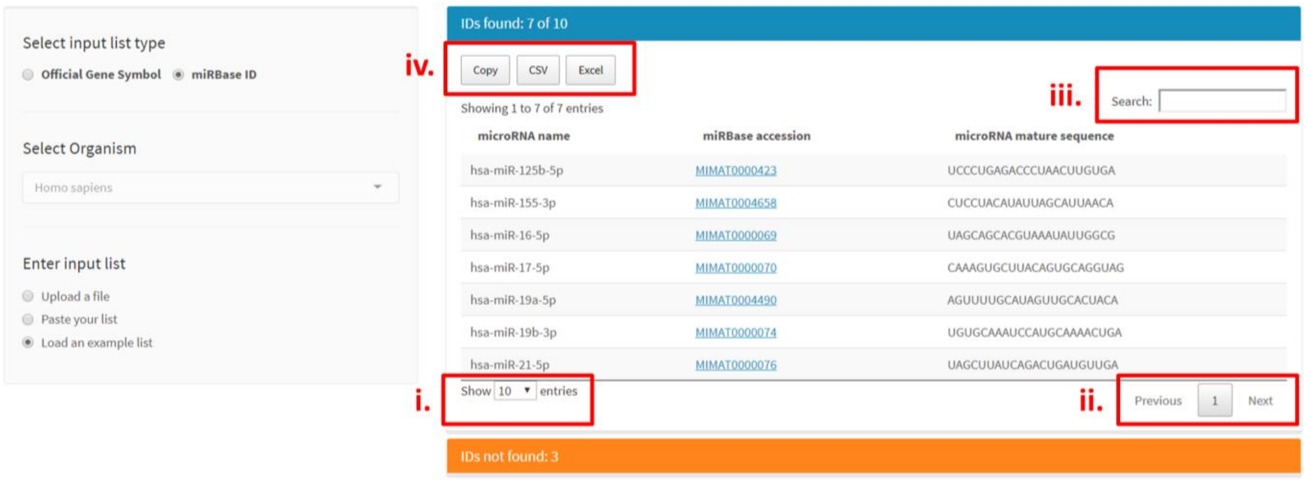

The list of not recognized miRNAs (if any) can be viewed by clicking on the **IDs not found** collapsible panel.

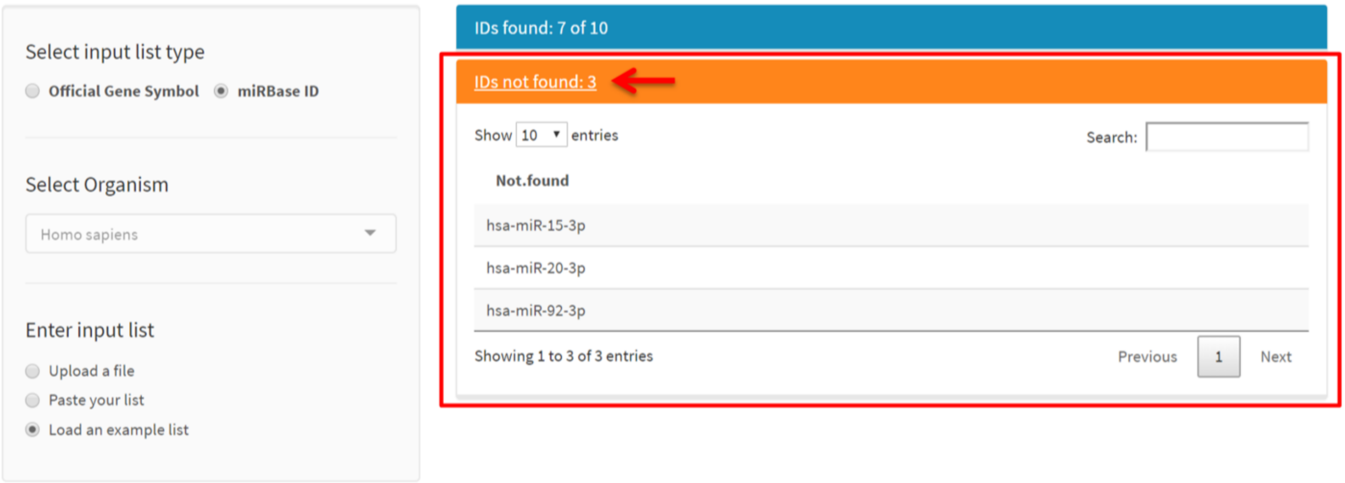

An error message appears either if the input list contains all miRBase IDs that are not valid or if the organism is incorrectly selected.

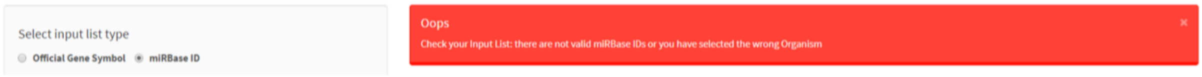

miRNA-target Enrichment link

By clicking on **miRNA-target Enrichment** link, MIENTURNET will perform a statistical analysis for over-representation of miRNA-target interactions in the input miRNAs list.

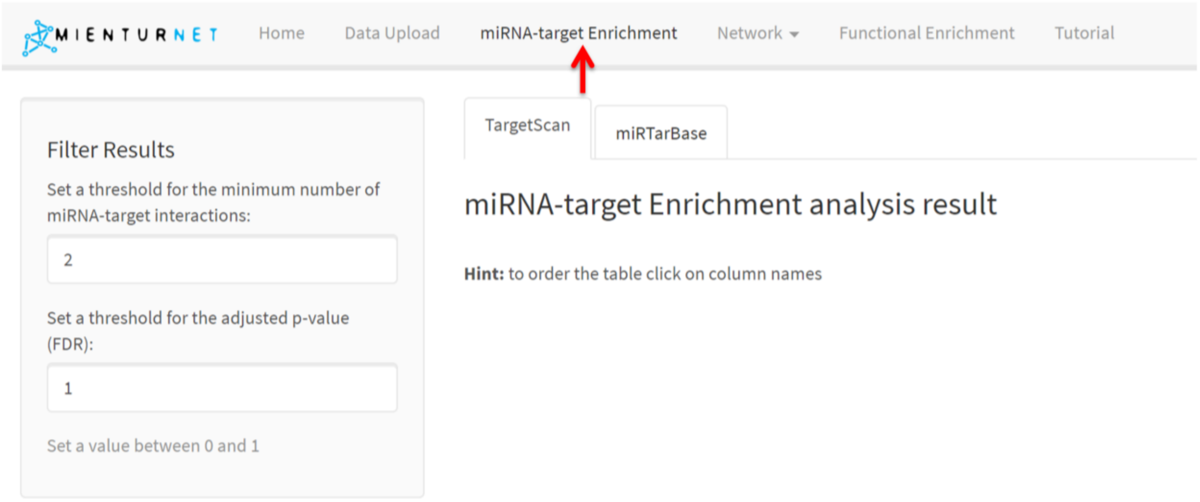

The user has the possibility to perform the enrichment analysis by using a threshold on:

i. the minimum number of miRNA-target interactions (default is 2)
ii. the adjusted p-values (default is 1)

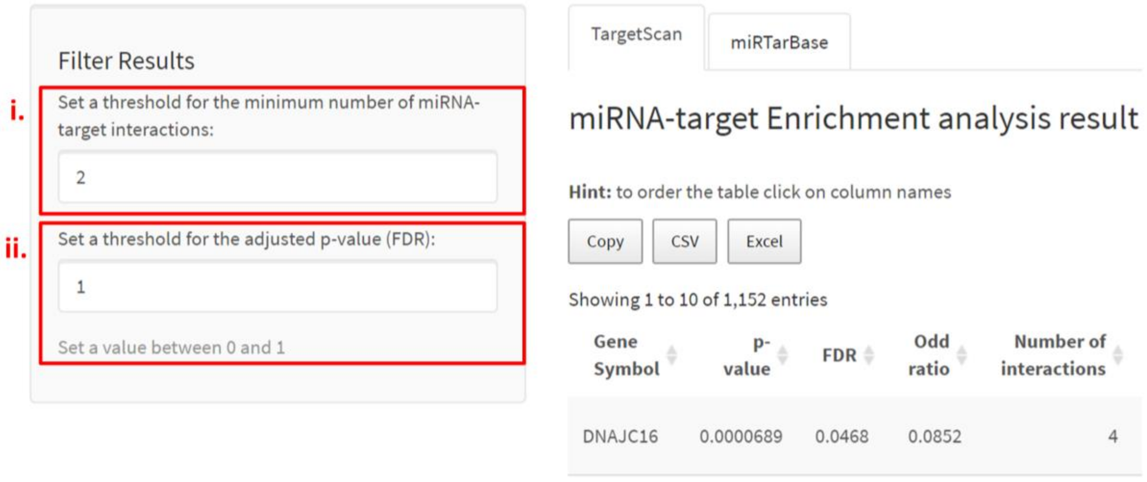

From **miRNA-target Enrichment** page, the user can choose the reference database for the miRNA-target interactions (i.e., TargetScan and/or miRTarBase) to be used to perform the enrichment analysis.

Caveat: regardless of the database choice, MIENTURNET performs the miRNA-target enrichment analysis searching for both predicted and validated interactions. A progress bar in action will appear at the bottom-right of the page as long as both analyses are not completed.

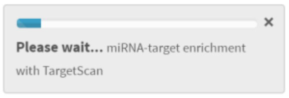

By clicking on **TargetScan** tab, the computationally predicted miRNA-target interactions will be considered.

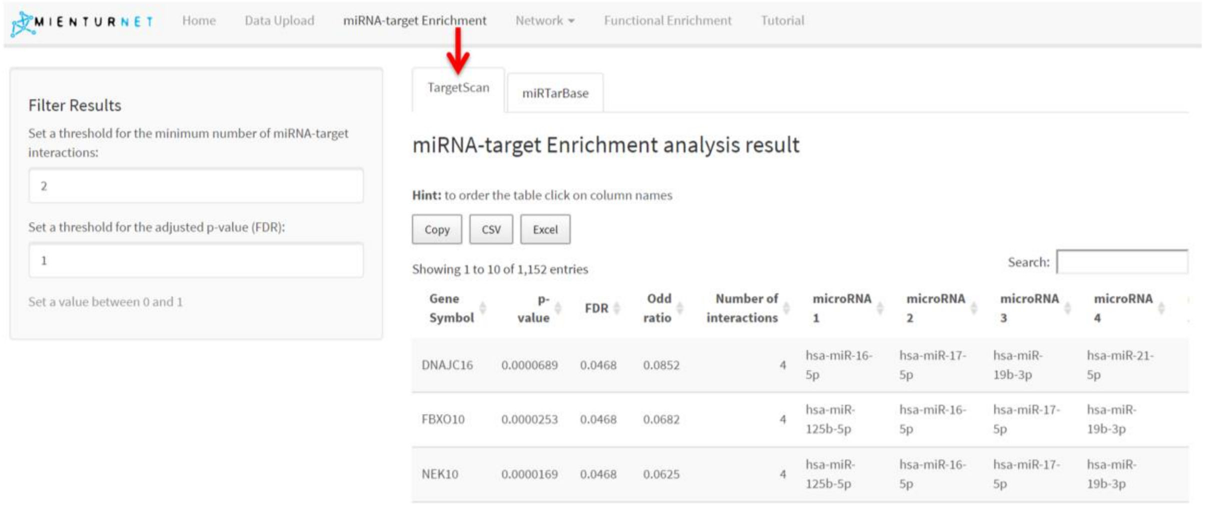

Caveat: TargetScan predicts biological targets of miRNAs by searching for the presence of conserved sites that match the seed region (i.e., the region comprising nucleotides 2-7 at the 5′-end of the mature miRNA sequence) of each miRNA [3]. Note that, a miRNA family is comprised of miRNAs with the same seed sequence. This implies that members of the same miRNA family all share the same predicted targets. As a consequence, in this case, the enrichment analysis was performed considering miRNA families instead of single miRNAs.

By clicking on **miRTarBase** tab, the experimentally validated miRNA-target interactions will be considered.

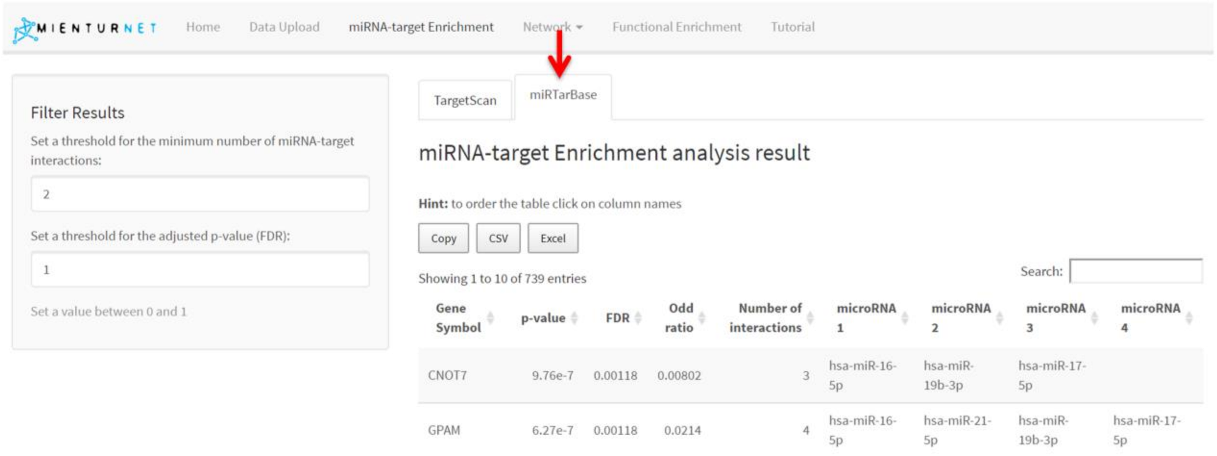

Caveat: miRTarBase collects miRNA-target interactions experimentally validated by reporter assay, western blot, microarrays, and next-generation sequencing experiments. As a consequence, in this case, the enrichment analysis was performed considering miRNAs instead of miRNA families.

For each gene in the chosen database (TargetScan or miRTarBase), the hypergeometric test was used to calculate the significance (p-value <0.05) of the enrichment of the input list in miRNAs targeting that gene.

The p-value is computed as

$$p = 1 - \sum_{i=0}^{X-1} \frac{\binom{K}{i}\binom{M-K}{N-i}}{\binom{M}{N}}$$

where M is the dimension of the universe, that is the number of all predicted (validated) miRNA-target interactions encompassed in TargetScan (miRTarBase); K is the number of predicted (validated) miRNA-target interactions encompassed in TargetScan (miRTarBase) for the selected target gene; N is the number of miRNA families (number of miRNAs) of the input list recognized by TargetScan (miRTarBase); X is the number of miRNA families (number of miRNAs) for which predicted (validated) miRNA-target interactions, for the selected gene, exist.

The results of the enrichment analysis are presented as[1]:

i. a bar plot where the Y-axis refers to the top ten target genes resulted from the enrichment analysis, while the X-axis represents the number of miRNAs targeting them. The color code reflects the FDR value increasing from red to blue. This plot is resizable by grabbing and dragging an active corner at the bottom right of the page.

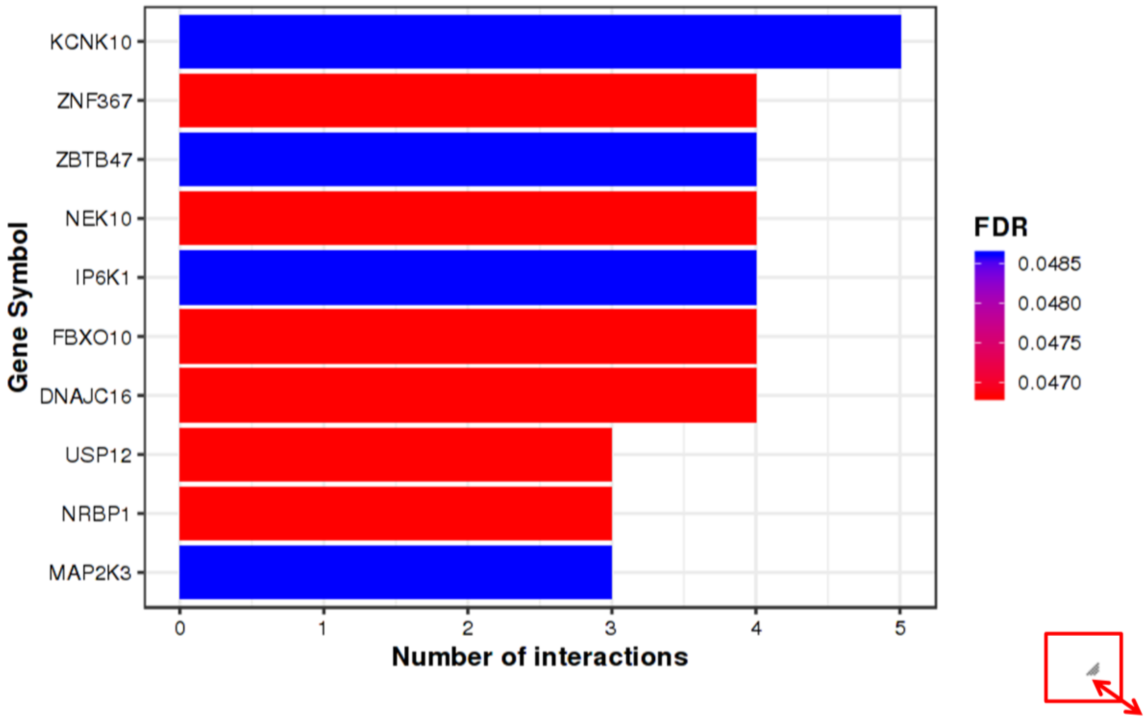

[1] Hereafter, all snapshots refer to TargetScan visualization.

ii. an interactive table where the user has also always the possibility to:

1. visualize the first 10, 25, 50, 100 entries by clicking the drop-down menu at the bottom-left
2. navigate the table trough the navigation controller buttons at the bottom-right
3. search for a miRNA or a Target Gene of interest by typing the name in the search bar at the top-right
4. copy the table to the clipboard, export it in a comma separated format (CSV) or in EXCEL format through the dedicated buttons placed at the top-left

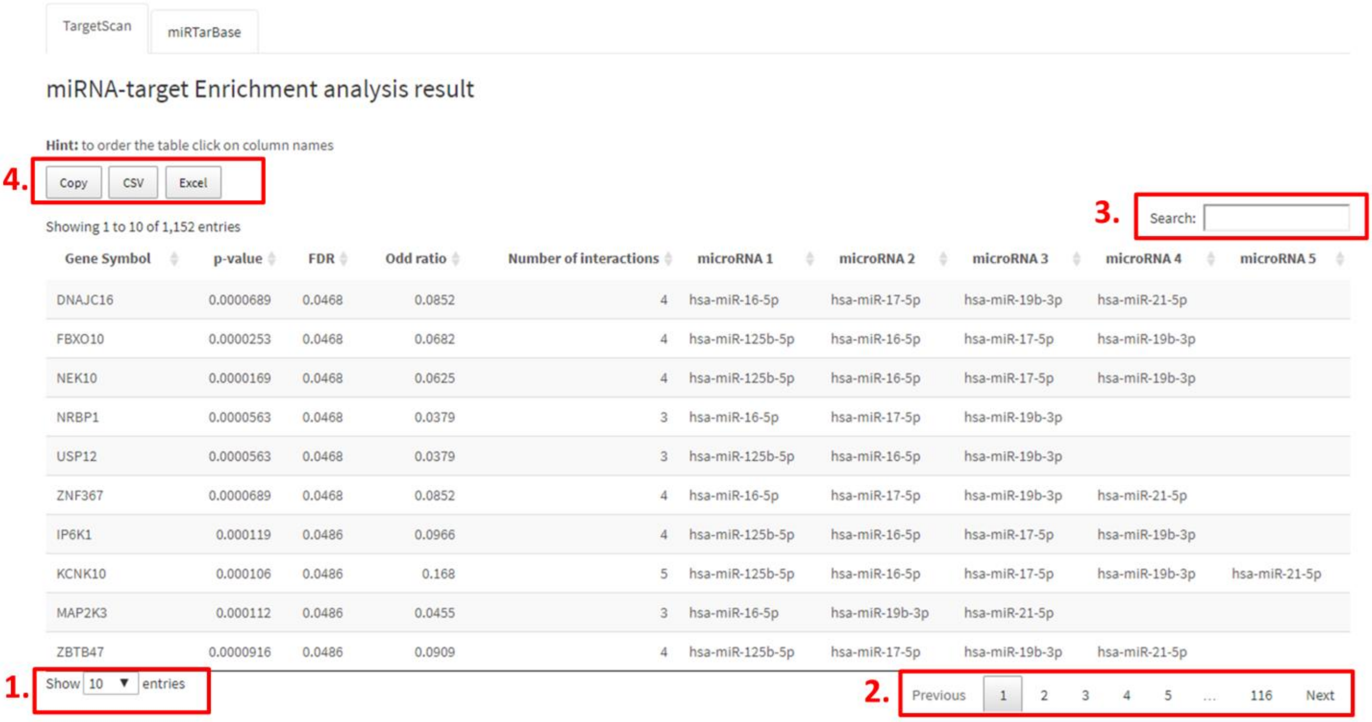

Gene Symbol	p-value	FDR	Odd ratio	Number of interactions	microRNA 1	microRNA 2	microRNA 3	microRNA 4	microRNA 5
DNAJC16	0.0000689	0.0468	0.0852	4	hsa-miR-16-5p	hsa-miR-17-5p	hsa-miR-19b-3p	hsa-miR-21-5p	
FBXO10	0.0000253	0.0468	0.0682	4	hsa-miR-125b-5p	hsa-miR-16-5p	hsa-miR-17-5p	hsa-miR-19b-3p	
NEK10	0.0000169	0.0468	0.0625	4	hsa-miR-125b-5p	hsa-miR-16-5p	hsa-miR-17-5p	hsa-miR-19b-3p	
NRBP1	0.0000563	0.0468	0.0379	3	hsa-miR-16-5p	hsa-miR-17-5p	hsa-miR-19b-3p		
USP12	0.0000563	0.0468	0.0379	3	hsa-miR-125b-5p	hsa-miR-16-5p	hsa-miR-19b-3p		
ZNF367	0.0000689	0.0468	0.0852	4	hsa-miR-16-5p	hsa-miR-17-5p	hsa-miR-19b-3p	hsa-miR-21-5p	
IP6K1	0.000119	0.0486	0.0966	4	hsa-miR-125b-5p	hsa-miR-16-5p	hsa-miR-17-5p	hsa-miR-19b-3p	
KCNK10	0.000106	0.0486	0.168	5	hsa-miR-125b-5p	hsa-miR-16-5p	hsa-miR-17-5p	hsa-miR-19b-3p	hsa-miR-21-5p
MAP2K3	0.000112	0.0486	0.0455	3	hsa-miR-16-5p	hsa-miR-19b-3p	hsa-miR-21-5p		
ZBTB47	0.0000916	0.0486	0.0909	4	hsa-miR-125b-5p	hsa-miR-17-5p	hsa-miR-19b-3p	hsa-miR-21-5p	

The columns of the table report:

- *Gene Symbol:* names of target genes
- *p-value:* p-values of the enrichment analysis
- *FDR:* the adjusted p-values obtained by using the Benjamini-Hochberg (False Discovery Rate, FDR) procedure for multiple testing
- *Odd ratio:* ratios calculated as: $\frac{K/M}{X/N}$
- *Number of interactions:* total numbers of interactions for each gene
- Additional extra columns reporting the names of miRNA of the input list for each gene, named with increasing numerical values

Network menu

In order to build the network of miRNA-target interactions identified by the enrichment analysis, click on **Network** menu and choose the reference database (i.e., TargetScan and/or miRTarBase) from the drop-down menu.

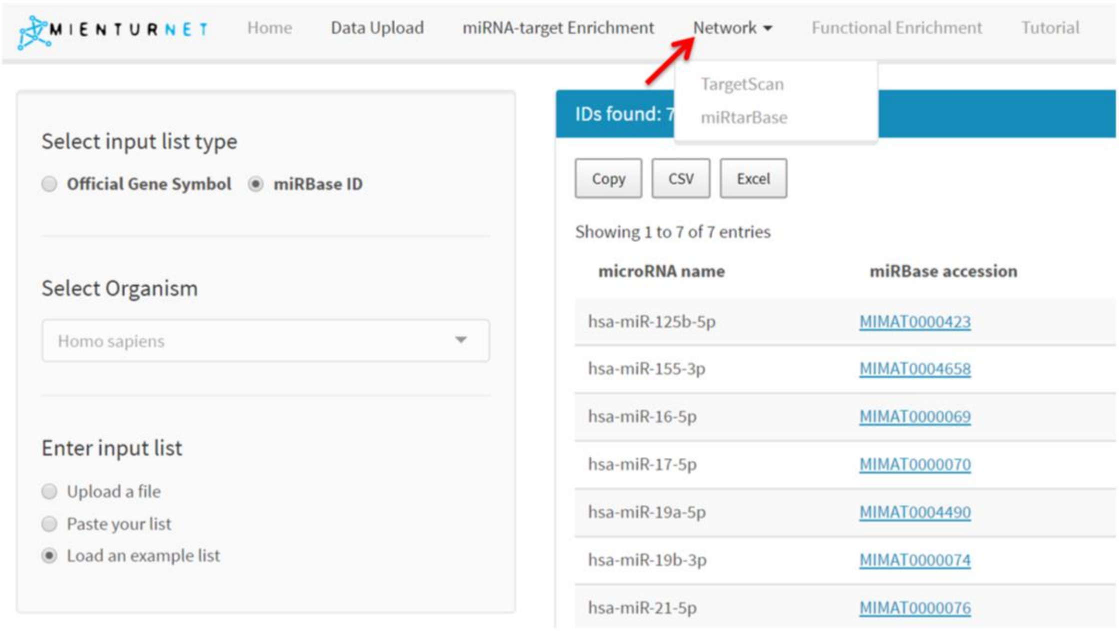

Selecting miRNA-target interactions database and filtering

By choosing **TargetScan**, the results can be filtered by using a threshold on:

i. the minimum number of miRNA-target interactions
ii. the adjusted p-values obtained from the miRNA-target enrichment analysis
iii. the *cumulative weighted context++ score* (CWCS) parameter [4] provided by TargetScan[2]
iv. the *probability of conserved targeting* (P_{CT}) parameter [5] provided by TargetScan

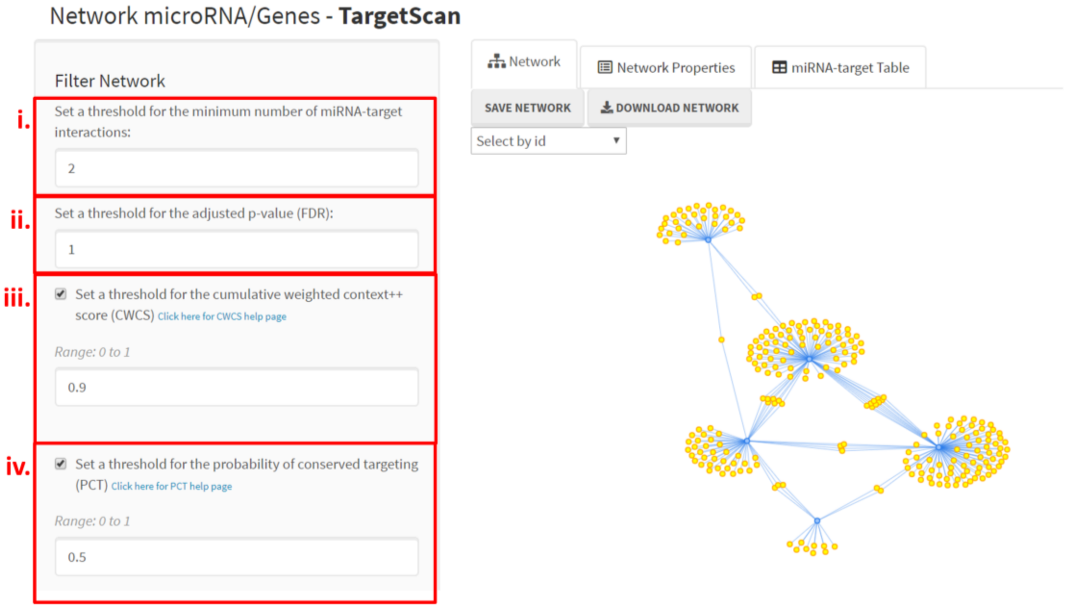

[2] This threshold refers to a percentile of the CWCS distribution.

The user can choose to apply one of these filters, more than one or none. By default, the first two thresholds are set to the value chosen in the **miRNA-target Enrichment** page. The other two parameters, CWCS and P_{CT}, are set by default to 0.9 and 0.5, respectively.

By choosing **miRTarBase**, the results can be filtered according to

i. the minimum number of miRNA-target interactions
ii. the adjusted p-values obtained from the miRNA-target enrichment analysis
iii. the type of *Evidence categories* used by miRTarBase to validate the miRNA-target interactions:
    a. ‘Strong’ for considering strong experimental methods (e.g., Luciferase assay, Western)
    b. ‘Weak’ for considering weaker experimental evidence (e.g., CLIP)
    c. ‘Strong and Weak’ for considering both strong and weak experimental methods

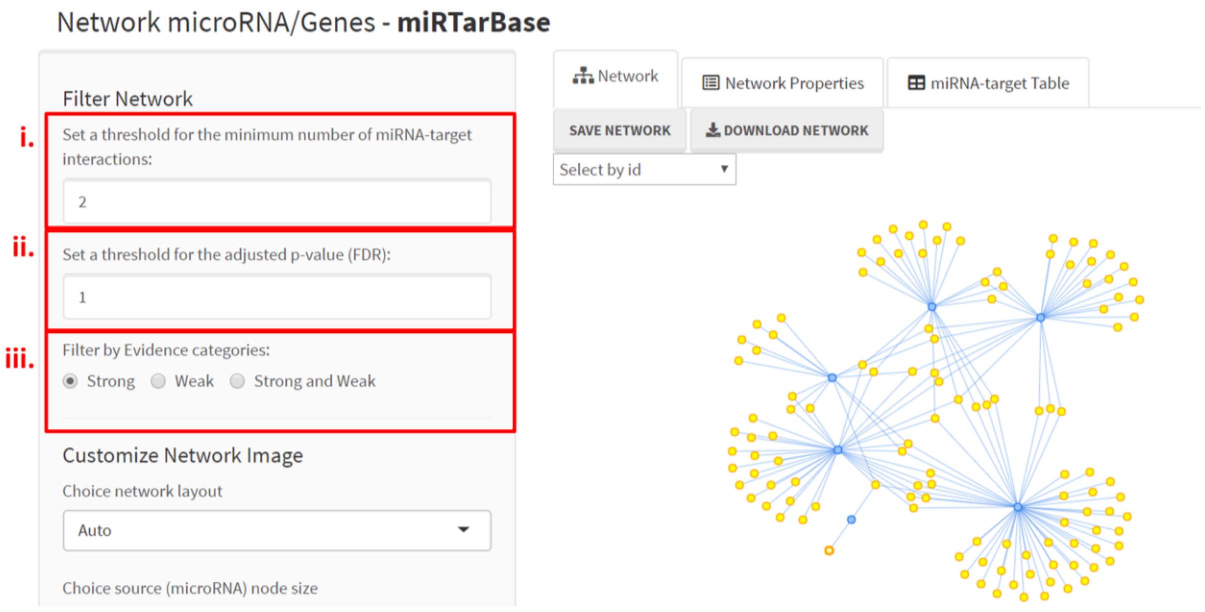

As before, by default the first two thresholds are set to the value chosen in the **miRNA-target Enrichment** page. Instead, the *Evidence categories* filter is configured as ‘Strong’.

Caveat: regardless of the database choice, the user that is not interested in the enrichment analysis can skip the previous step (i.e., the user can avoid to click on **miRNA-target Enrichment** link) and jump to the next step of **Network** menu. In this case, MIENTURNET will still perform the enrichment analysis, but will set the default value for the adjusted p-values to 1.

Network visualization and analysis

Both for **TargetScan** and **miRTarBase**, the user can interactively play with the miRNA-interaction network by pressing the following tabs:

1. **Network** tab: it allows to visualize, customize, and download the network in the form of an edgelist (i.e., a 2-column table where each row represents a single edge)
2. **Network properties** tab: it allows to perform a network analysis
3. **miRNA-target Table** tab: it allows to view and download a table where the columns are the miRNAs with the corresponding own targets

In the following, there is a detailed description for each of these tabs[3].

1. Network tab

From **Network** tab, the user can visualize the miRNA-target interaction network:

i. as an image that can be interactively explored and customized
ii. as a 2-column table in the form of an edgelist (i.e., each row represents a miRNA-target interaction).

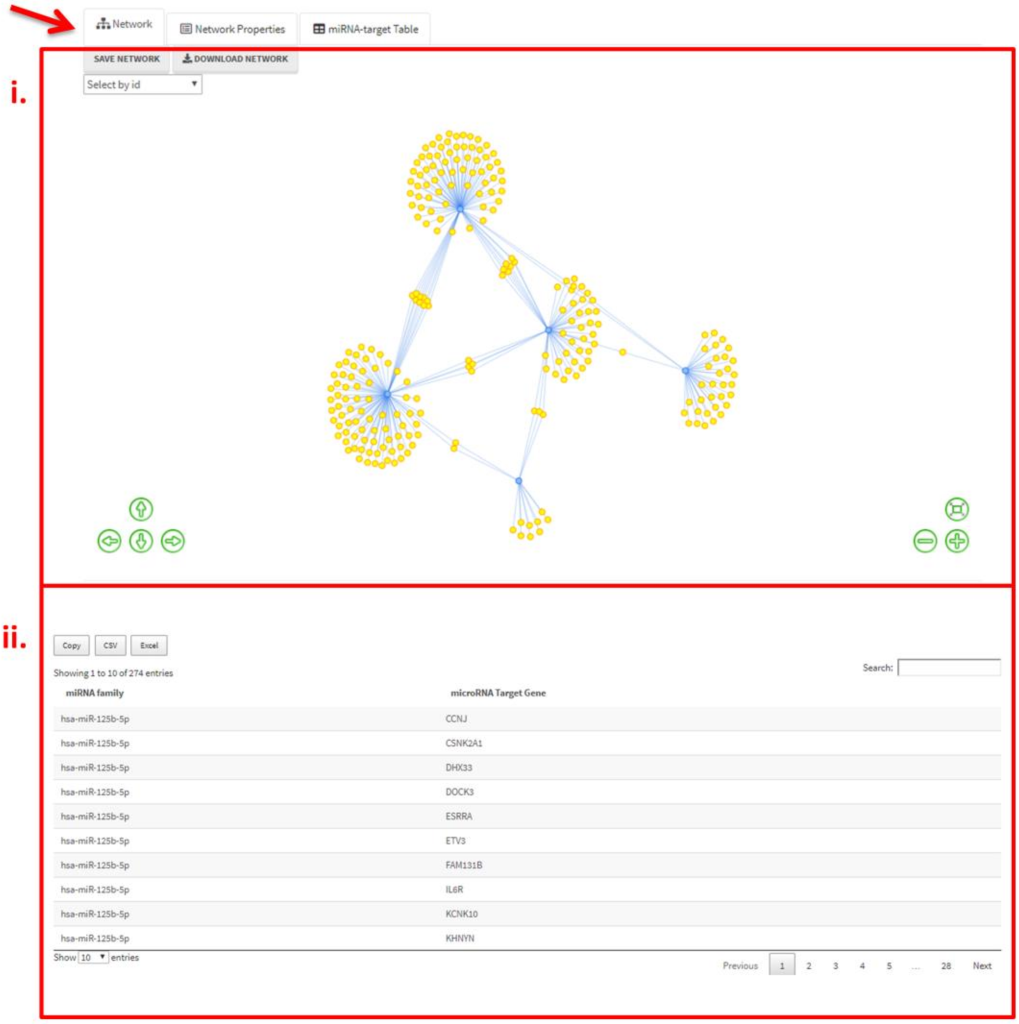

[3] All snapshots refer to TargetScan visualization, where source nodes are the miRNA families to which each miRNA of the input list belongs. Otherwise, in the miRTarBase visualization, source nodes are the miRNAs of the input list.

By using the *Customize network image* menu on the left, the user may customize:

- the network layout (details about the layout algorithms are available at http://igraph.org)
- the size of source/target nodes
- the shape of source/target nodes
- the color of source/target nodes. By default, source nodes (miRNA families or single miRNAs) are blue circles, while target nodes (mRNAs) are yellow circles.

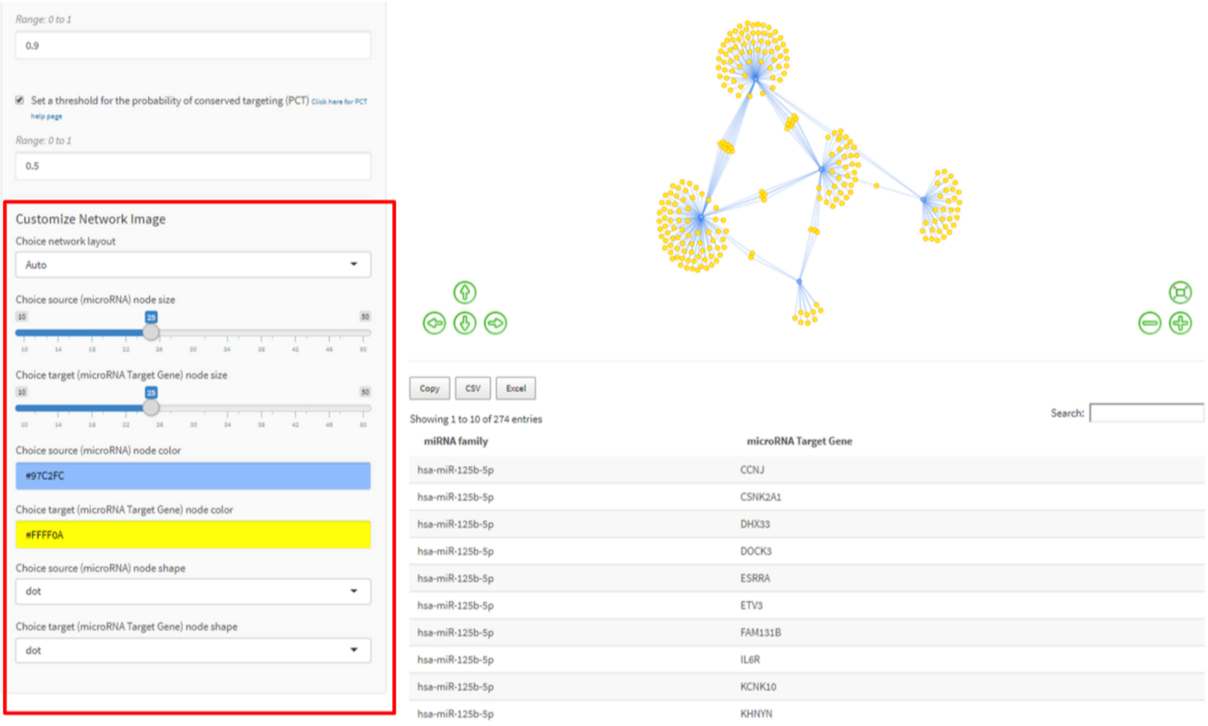

On the network image, the user has also the possibility to:

i. move the network around by using the up-down left-right arrows
ii. zoom in/out the image by using the plus/minus buttons
iii. select a specific node by scrolling down the drop-down menu "Select by id" at the top-left

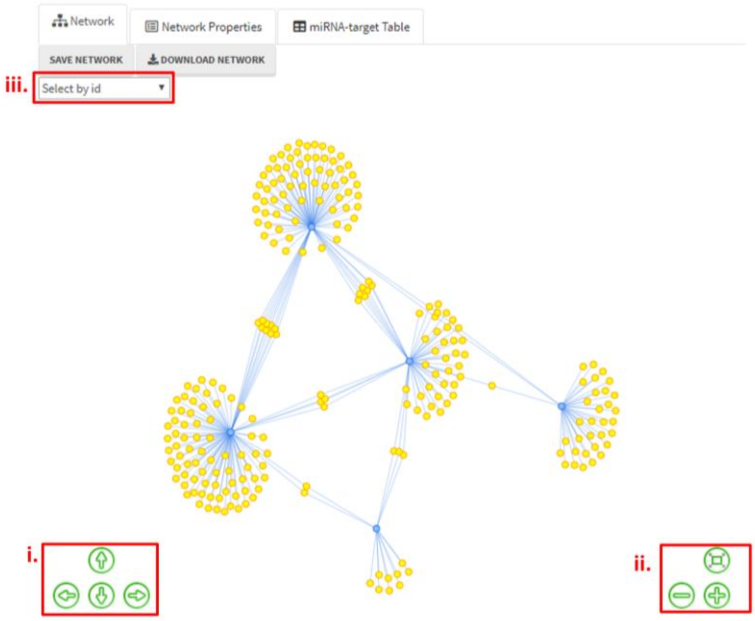

iv. select a specific node by placing the pointer over the node to be selected. Note, the selection will interest the node and its first neighbors. For example, if a miRNA is selected both miRNA and its targets will be highlighted.

v. move one or more node by selecting and dragging them with mouse

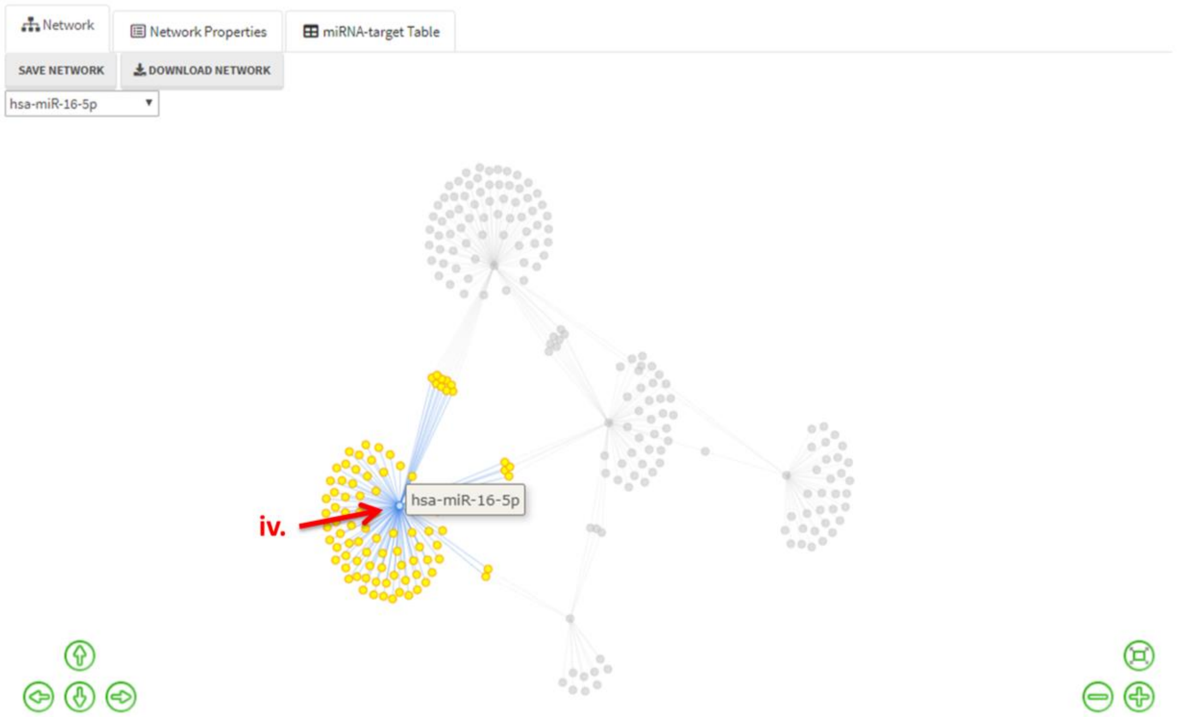

The user can export the network picture as:

i. a dynamic image in HTML format, by clicking first SAVE NETWORK button and then DOWNLOAD NETWORK button at the top-left

ii. a static image in PNG format, by pressing down on the right mouse button (i.e., right-clicking)

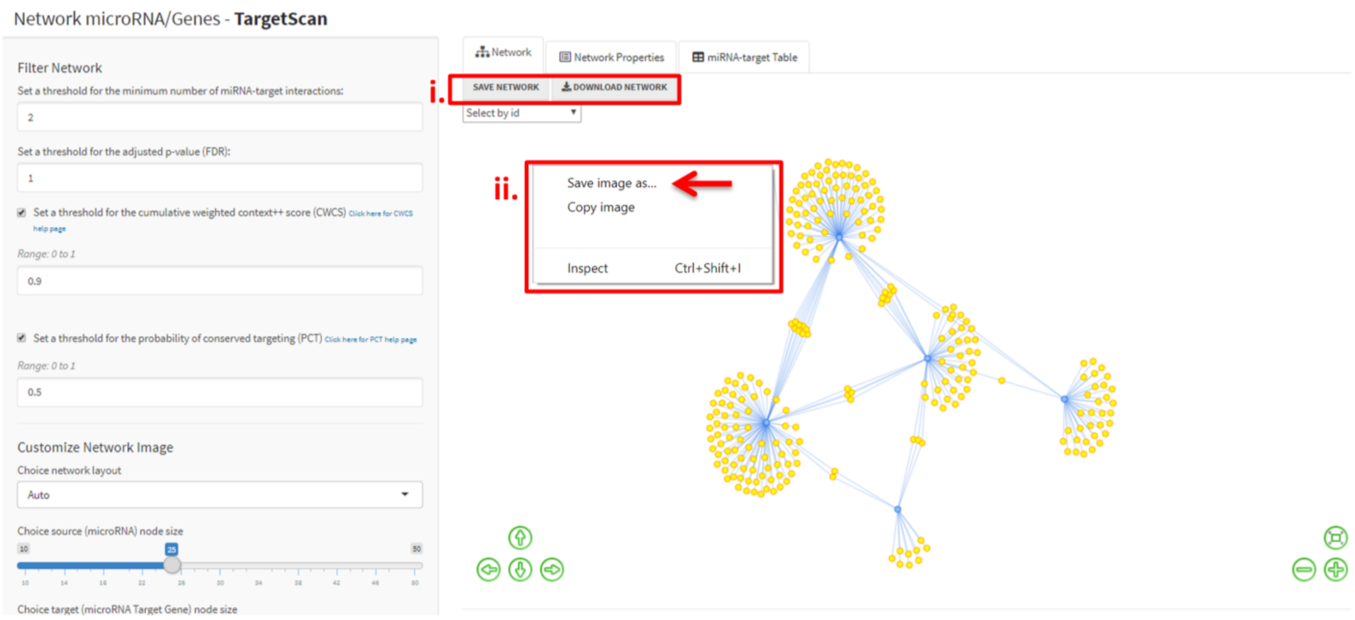

On the network table below the network image, the user has always the possibility to:

i. visualize the first 10, 25, 50, 100 entries by clicking the drop-down menu at the bottom-left
ii. navigate the table trough the navigation controller buttons at the bottom-right
iii. search for a miRNA or a target gene of interest by typing the name in the search bar at the top-right
iv. copy the table to the clipboard, export it in a comma separated format (CSV) or in EXCEL format through the dedicated buttons placed at the top-left.

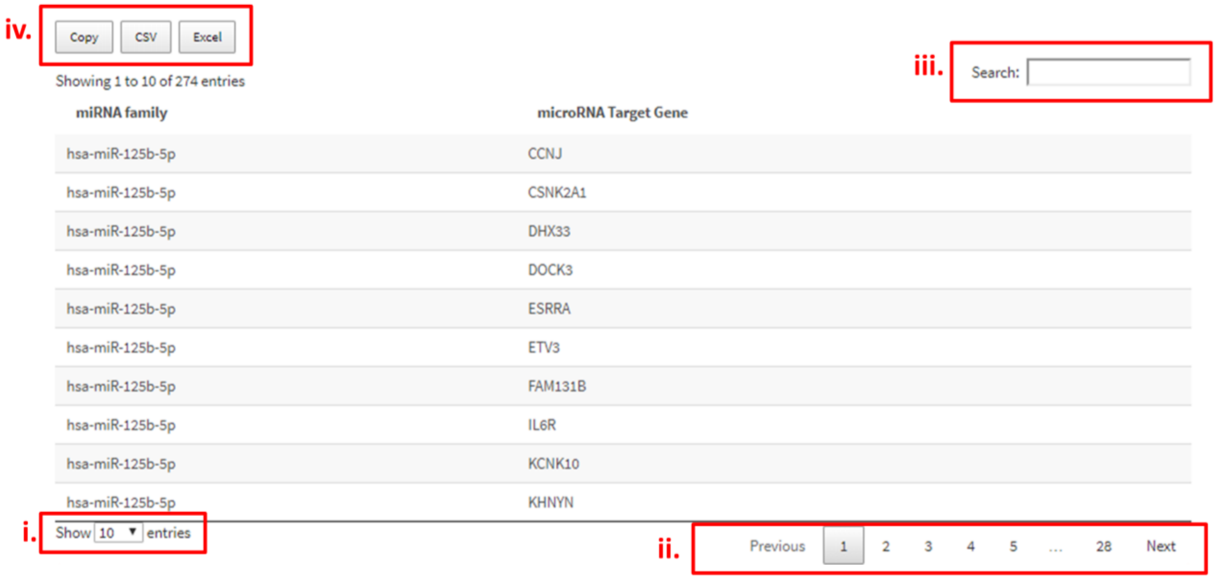

One or more miRNAs in the input list, although recognized in miRBase, could not be in the chosen database (TargetScan or miRTarBase). In this case, the miRNAs not found are listed in the **IDs not found** collapsible panel.

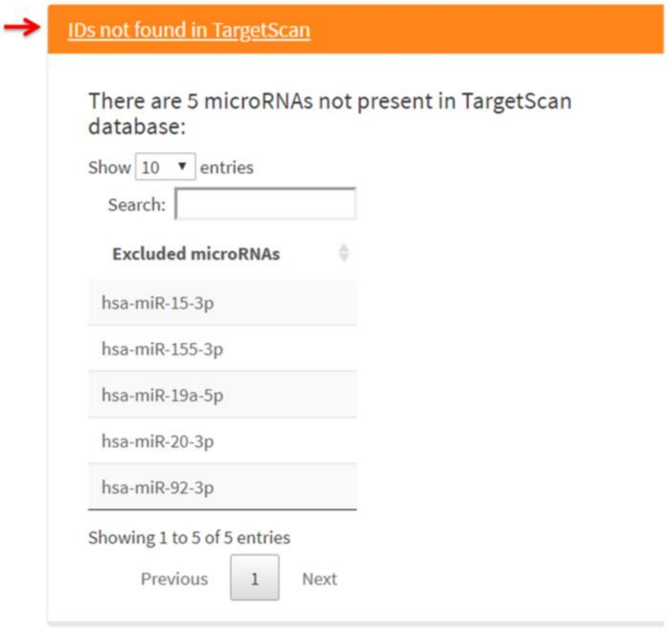

2. Network Properties tab

From **Network Properties** tab, the user can:

i. visualize a table with the topological properties for each node[4], where it is possible to:
   1. sort each column in decreasing or increasing order by clicking on the up-down arrows close to the column names
   2. visualize the first 10, 25, 50, 100 entries by clicking the drop-down menu at the bottom-left
   3. navigate the table trough the navigation controller buttons at the bottom-right
   4. search for a node of interest by typing the name in the search bar at the top-right
   5. copy the table to the clipboard, export it in a comma separated format (CSV) or in EXCEL format through the dedicated buttons placed at the top-left

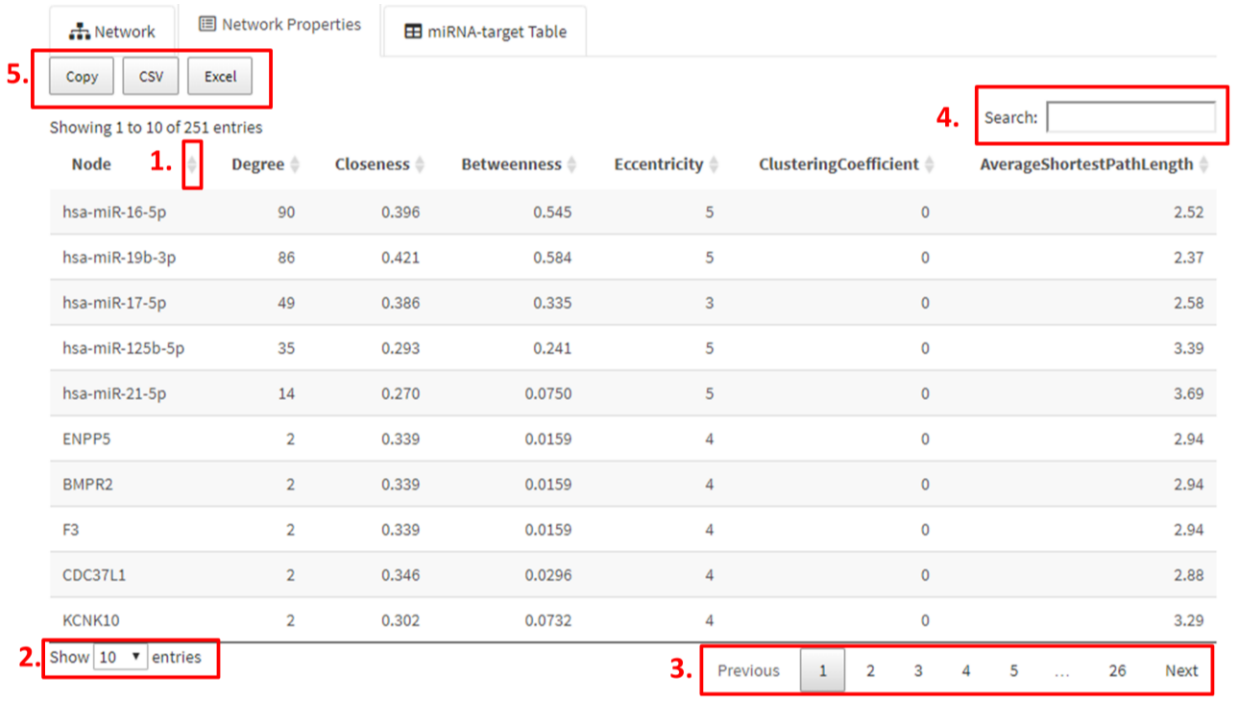

Node	Degree	Closeness	Betweenness	Eccentricity	ClusteringCoefficient	AverageShortestPathLength
hsa-miR-16-5p	90	0.396	0.545	5	0	2.52
hsa-miR-19b-3p	86	0.421	0.584	5	0	2.37
hsa-miR-17-5p	49	0.386	0.335	3	0	2.58
hsa-miR-125b-5p	35	0.293	0.241	5	0	3.39
hsa-miR-21-5p	14	0.270	0.0750	5	0	3.69
ENPP5	2	0.339	0.0159	4	0	2.94
BMPR2	2	0.339	0.0159	4	0	2.94
F3	2	0.339	0.0159	4	0	2.94
CDC37L1	2	0.346	0.0296	4	0	2.88
KCNK10	2	0.302	0.0732	4	0	3.29

In particular, the columns of the table report:

- *degree* - the number of incoming and outgoing edges of each node
- *betweenness* – the number of shortest paths[5] going through the node
- *average shortest path length* – the average length of the shortest paths between a node and all other nodes in the network
- *eccentricity* – the shortest path distance of a node from the farthest other node in the network
- *clustering coefficient* – measurement the probability that the adjacent nodes of a node are connected. The clustering coefficient is set to 0 for nodes with degree zero and one.
- *closeness* – the inverse of the average length of the shortest paths between a node and all other nodes in the network. Note that the closeness centrality measure is not well-defined for disconnected graphs. If there is no (directed) path between node *i* and *j*, then the total number of nodes is used in the formula instead of the path length.

ii. visualize the following network plots:

[4] Details about the network topological properties are available at http://igraph.org
[5] the shortest path between two nodes is the minimum number of consecutive edges connecting them

a. a bar plot of the mRNA degree, where X-axis refers to the first 30 target genes (sorted in a decreasing order according to the degree) and Y-axis refers to their degree (i.e., number of miRNA targeting them)

b. a bar plot of the miRNA degree, where X-axis refers to the first 30 miRNAs (sorted in a decreasing order according to the degree) and Y-axis refers to their degree (i.e., number of their targets)

c. the nodes degree distribution $P(k)$, with k being the degree, by plotting $\log k$ on the X-axis and $\log P(k)$ on the Y-axis. Then, the method of least squares is used to find the best-fitting curve $\log P \sim \alpha \log k$, where α is the constant to estimate. The sum of the squared residuals R^2 (i.e., the sum of the vertical deviations from the best-fitting line) is reported together with the estimation of the coefficient α.

All plots can be exported as a compressed .zip file by clicking on SAVE DEGREE PLOTS button.

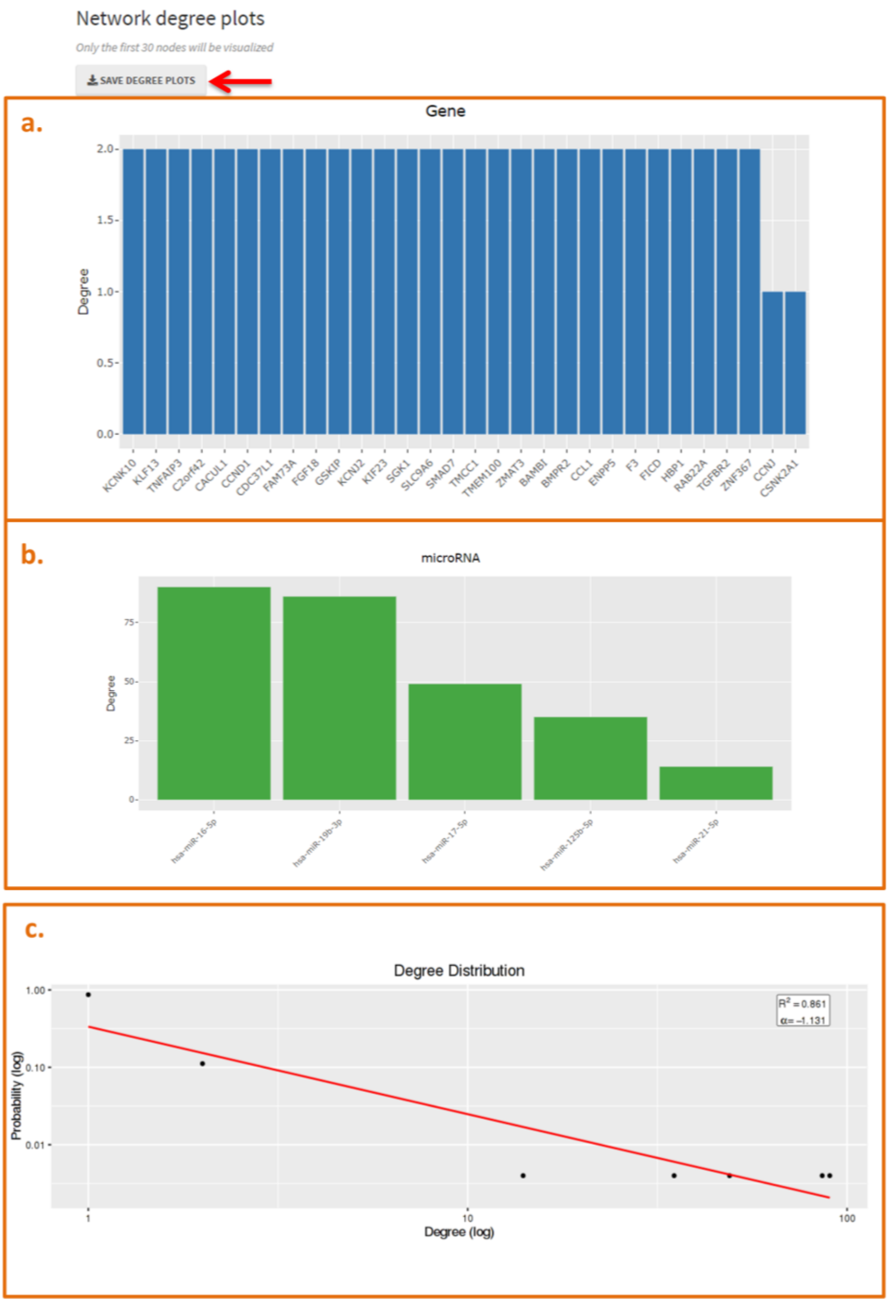

3. miRNA-target Table tab

From **miRNA-target Table** tab, the user can visualize an interactive table where the columns are the miRNAs of the input list found in the selected database (TargetScan or miRTarBase) with the corresponding own targets. The columns of the table are sorted in a decreasing order according to the number of target genes of each mRNA. The user has always the possibility to:

i. visualize the first 10, 25, 50, 100 entries by clicking the drop-down menu at the top-left
ii. navigate the table trough the navigation controller buttons at bottom-right
iii. search for a miRNA or a Target Gene of interest by typing the name in the search bar at the top-right
iv. copy the table to the clipboard, export it in a comma separated format (CSV) or in EXCEL format through the dedicated buttons placed at the top-left.

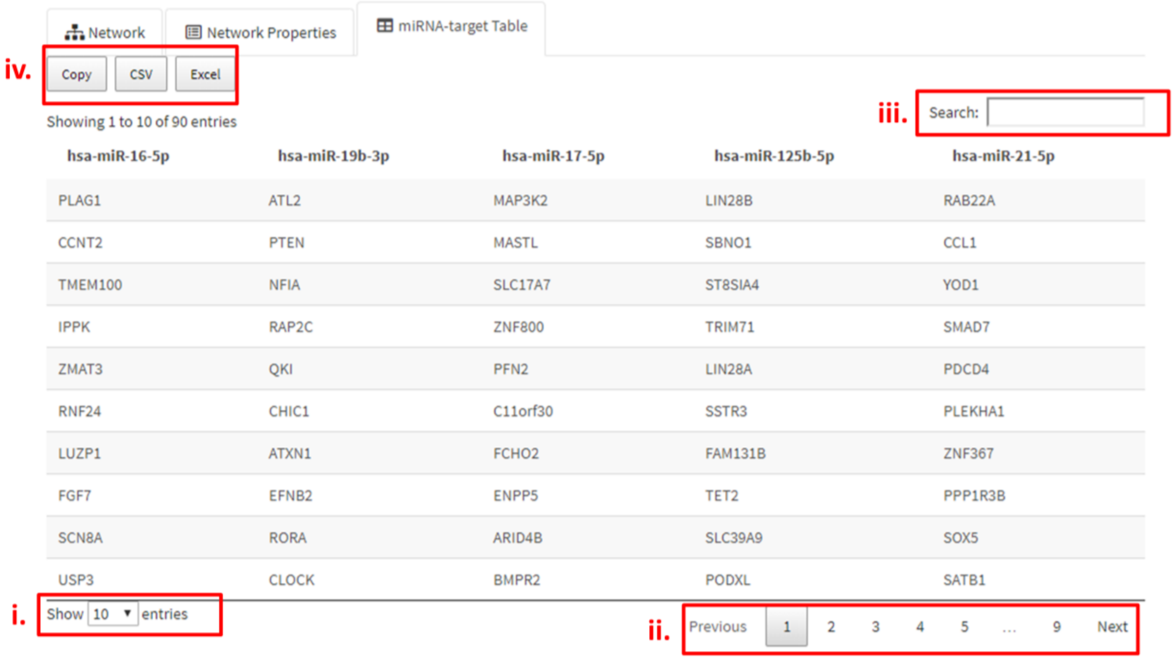

hsa-miR-16-5p	hsa-miR-19b-3p	hsa-miR-17-5p	hsa-miR-125b-5p	hsa-miR-21-5p
PLAG1	ATL2	MAP3K2	LIN28B	RAB22A
CCNT2	PTEN	MASTL	SBNO1	CCL1
TMEM100	NFIA	SLC17A7	ST8SIA4	YOD1
IPPK	RAP2C	ZNF800	TRIM71	SMAD7
ZMAT3	QKI	PFN2	LIN28A	PDCD4
RNF24	CHIC1	C11orf30	SSTR3	PLEKHA1
LUZP1	ATXN1	FCHO2	FAM131B	ZNF367
FGF7	EFNB2	ENPP5	TET2	PPP1R3B
SCN8A	RORA	ARID4B	SLC39A9	SOX5
USP3	CLOCK	BMPR2	PODXL	SATB1

Functional Enrichment link

In order to perform the functional enrichment analysis of target genes of selected miRNAs, click on **Functional Enrichment** link and choose TargetScan or miRTarBase [6].

Currently, MIENTURNET allows to query the following annotation databases: KEGG, REACTOME, WikiPathways, Disease Ontology. Note that the last one will be available only when *Homo sapiens* is the selected organism.

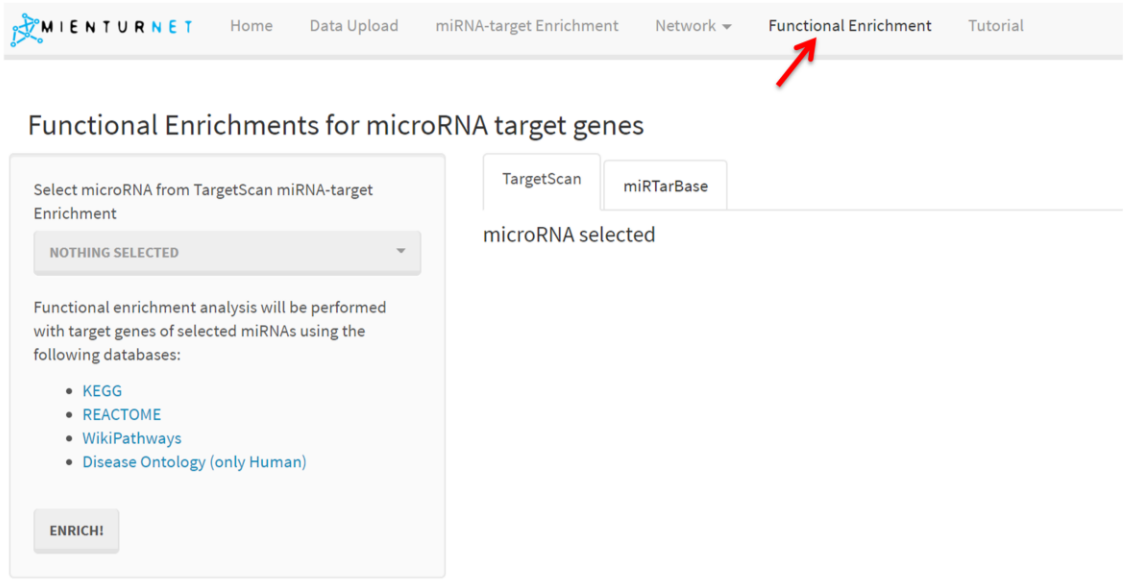

The user can select one or more miRNA families (or single miRNA in the case of miRTarBase) by clicking on each item or by typing the name in the search box. In this example, the first three miRNA families have been selected.

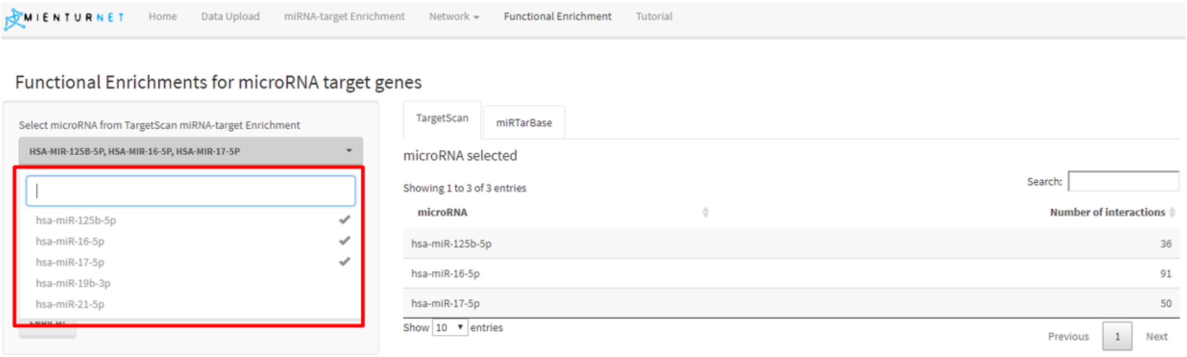

[6] All snapshots refer to TargetScan visualization, where for each selected miRNA the family to which it belongs will be reported. Otherwise, in the miRTarBase visualization, the selected miRNAs will be considered.

The selected miRNA families will appear in a table on the right along with the number of their interactions.

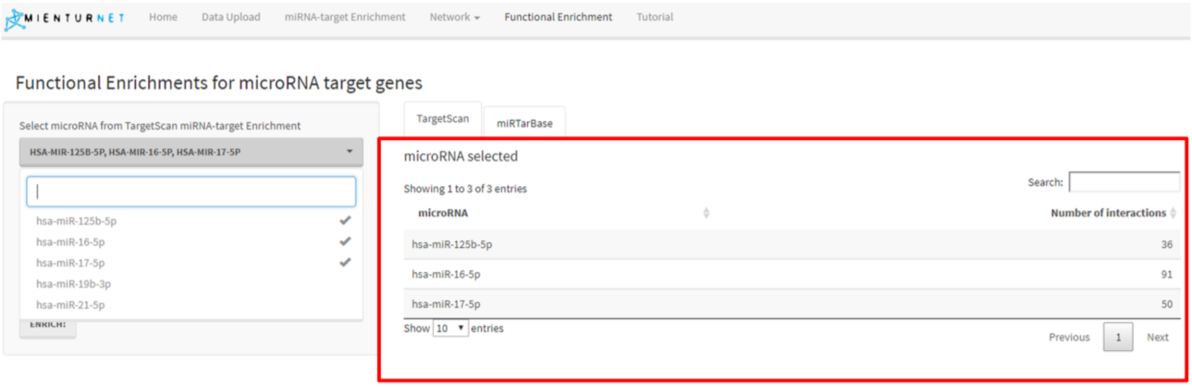

To start the analysis, click the ENRICH button.

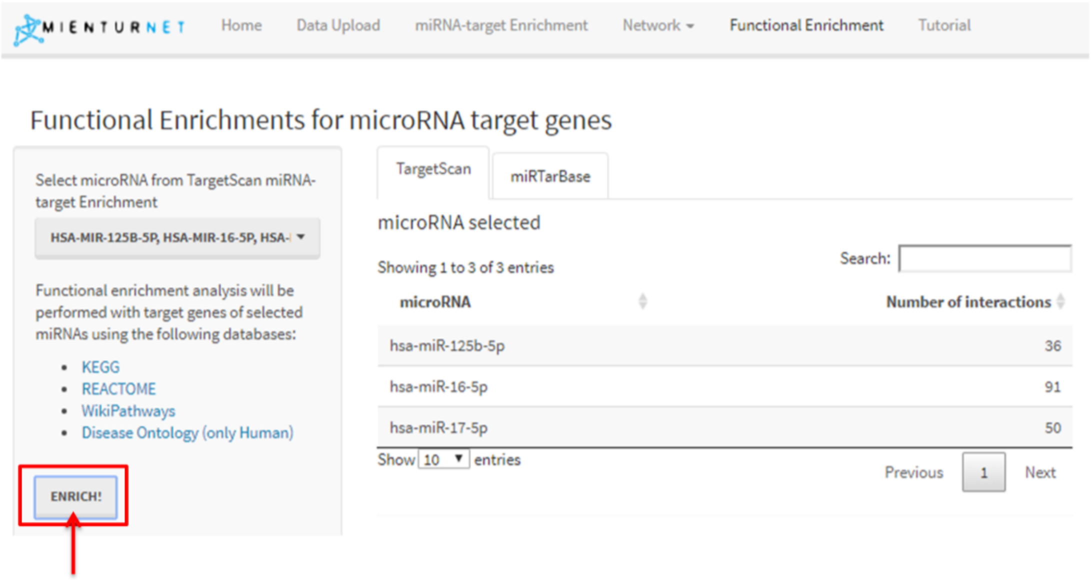

Caveat: The function enrichment analysis will regard the targets of each selected miRNA, separately. The user could see a progress bar in action at the bottom-right of the page.

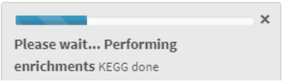

For each annotation database, the results of the functional enrichment analysis are presented as[7]:

1. an interactive table reporting: the miRNA name, the category name of the annotation database (e.g., KEGG ID), the category description, the p-value and the adjusted p-value (FDR), the list of target genes, the number of targets genes.
2. a dot plot, where the Y-axis reports the annotation categories (e.g., KEGG pathways) and the X-axis reports the miRNA family with the number of recognized targets in round brackets. The colors of the dots represent the adjusted p-values (FDR), whereas the size of the dots represents gene ratio (i.e., the number of miRNA targets found annotated in each category over the number of total genes annotated in that category). All the plots are resizable by grabbing and dragging an active corner at the bottom right of the page.

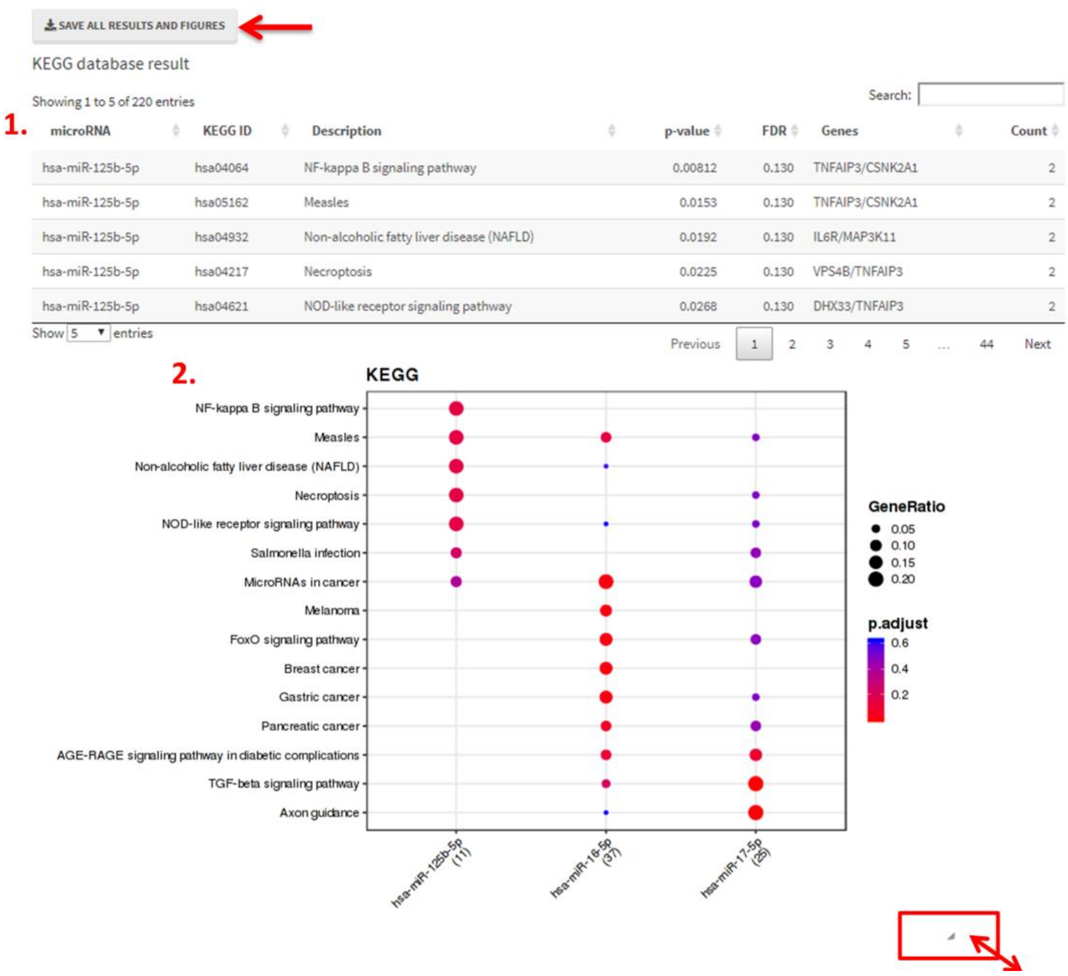
SAVE ALL RESULTS AND FIGURES

KEGG database result

Showing 1 to 5 of 220 entries

Search:

1.

microRNA	KEGG ID	Description	p-value	FDR	Genes	Count
hsa-miR-125b-5p	hsa04064	NF-kappa B signaling pathway	0.00812	0.130	TNFAIP3/CSNK2A1	2
hsa-miR-125b-5p	hsa05162	Measles	0.0153	0.130	TNFAIP3/CSNK2A1	2
hsa-miR-125b-5p	hsa04932	Non-alcoholic fatty liver disease (NAFLD)	0.0192	0.130	IL6R/MAP3K11	2
hsa-miR-125b-5p	hsa04217	Necroptosis	0.0225	0.130	VPS4B/TNFAIP3	2
hsa-miR-125b-5p	hsa04621	NOD-like receptor signaling pathway	0.0268	0.130	DHX33/TNFAIP3	2

Show 5 entries

Previous 1 2 3 4 5 ... 44 Next

[7] The results presented here refer to the KEGG enrichment analysis.

Entering a list of mRNAs

Data Upload link

If the user is interested to find miRNAs targeting a given list of mRNAs, from the **Data Upload** page, select **Official Gene Symbol** as type of the input list**.**

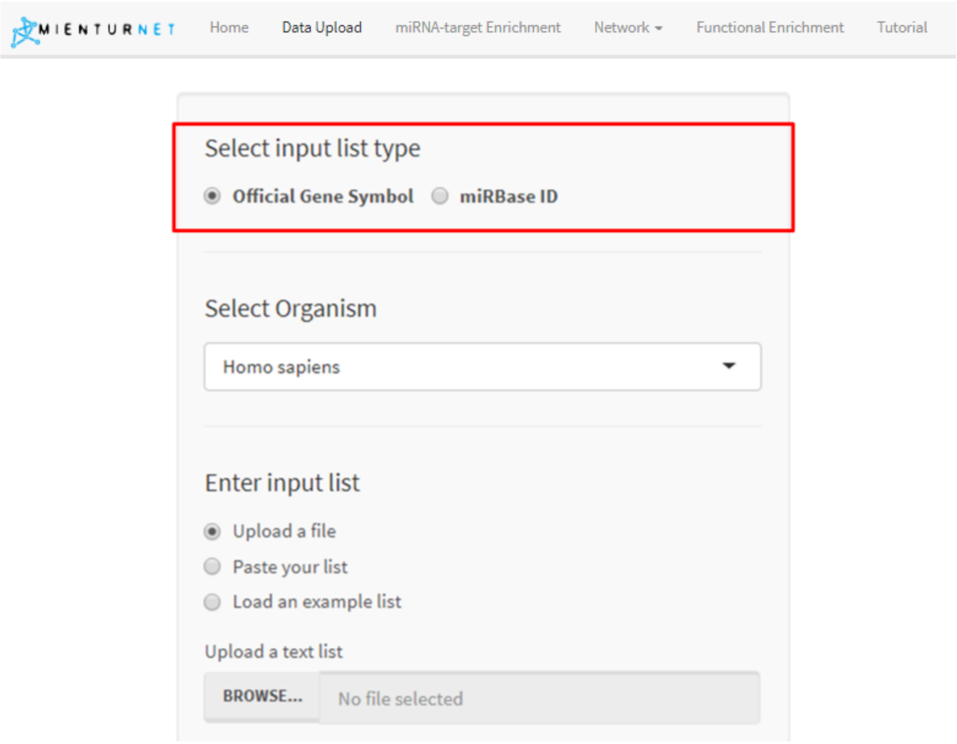

Select the organism from the drop-down menu.

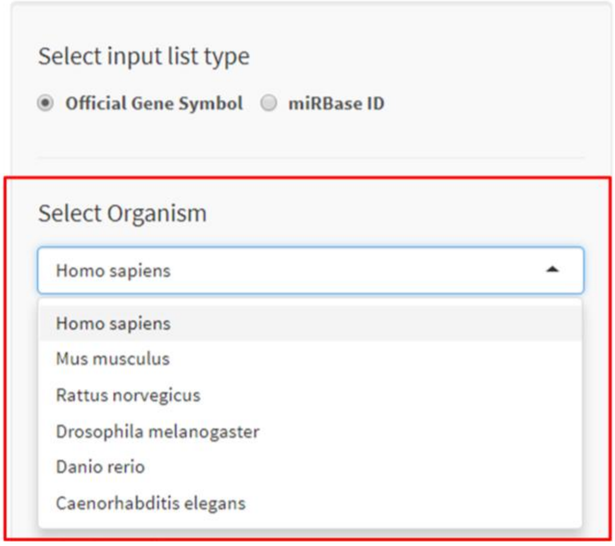

Enter the input list by using one of the following options:

1) **Upload a file** if the list is saved in a text file on the own computer and then click BROWSE to quickly access to the file

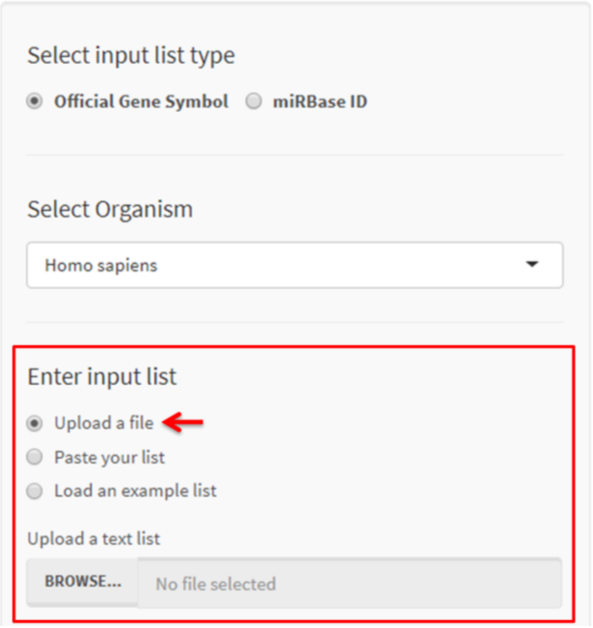

2) **Paste your list** in the specific text area and then click SUBMIT

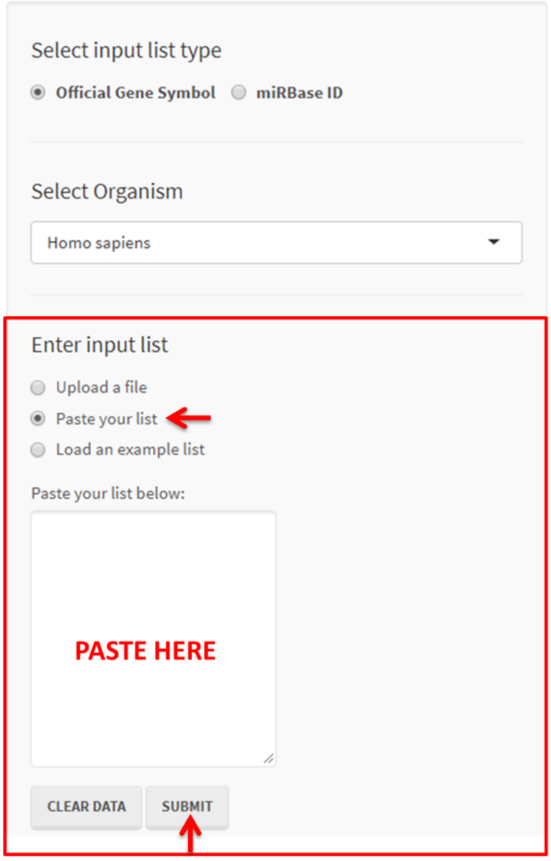

3) **Load an example list** for playing with data. With this choice, the user has the possibility to explore the functionality of MIENTURNET by exploiting a usage example.

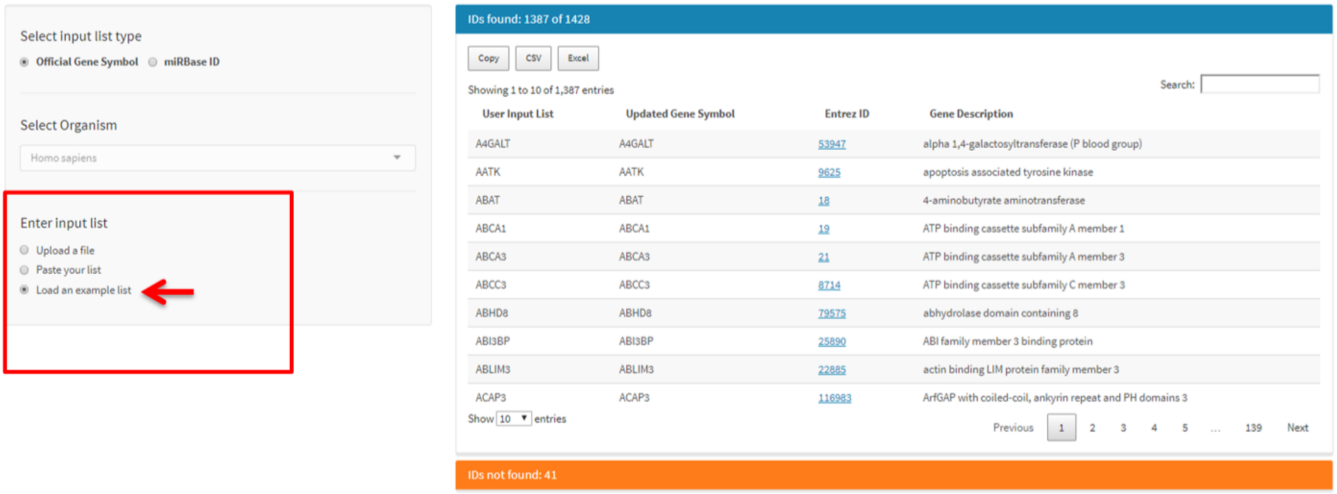

Hereafter, we will show how MIENTURNET works by loading the example input list of mRNAs selected by the authors. Note that this list represents the differentially expressed genes resulting from a recent computational study of glioblastoma cells that was aimed to identify genes playing a key role in the transition from stem-like tumor propagating cells towards differentiated glioblastoma cells [6].

Input list identifiers

The input list must include identifiers of mRNAs as Official Gene Symbol (e.g., PTEN for *human* species, Pten for *mouse* species). The tool processes the input file and immediately provides in the **IDs found** collapsible panel the total number of mRNAs recognized by NCBI database together with a table that includes: gene symbols of the input list, the corresponding updated gene symbols (i.e., the latest version of the gene name according to NCBI database), Entrez IDs as hyperlinks to NCBI Gene, and gene descriptions.

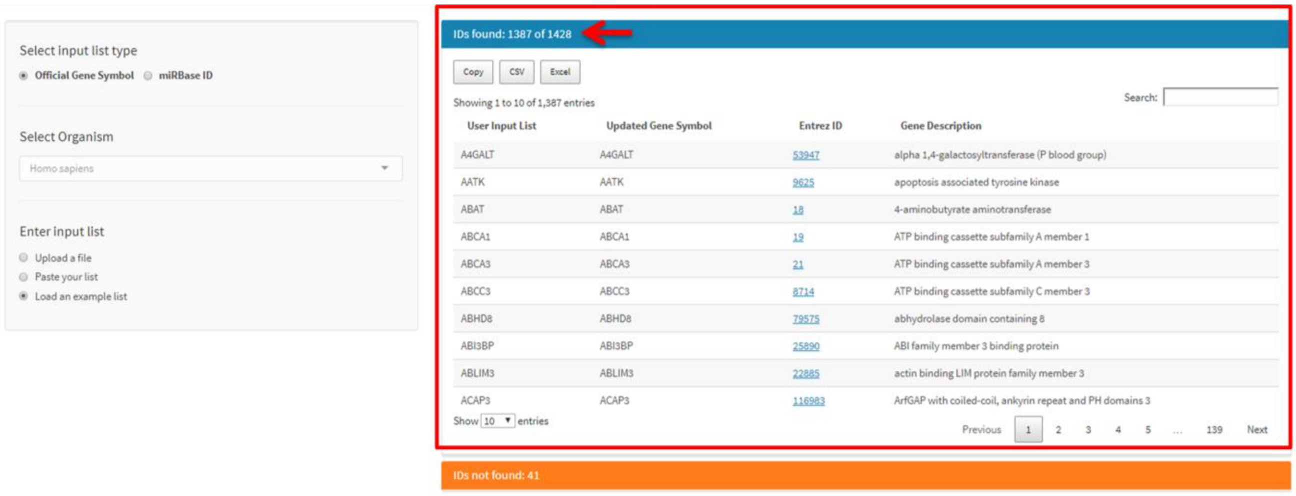

Caveat: the analysis will proceed by considering the updated gene symbols.

In the input list table, the user has also the possibility to:

i. visualize the first 10, 25, 50, 100 entries by clicking the drop-down menu at the bottom-left
ii. navigate the table trough the navigation controller buttons at the bottom-right
iii. search for a specific miRNA in the input list by typing the name in the search bar at the top-right.
iv. copy the table to the clipboard, export it in a comma separated format (CSV) or in EXCEL format through the dedicated buttons placed at the top-left.

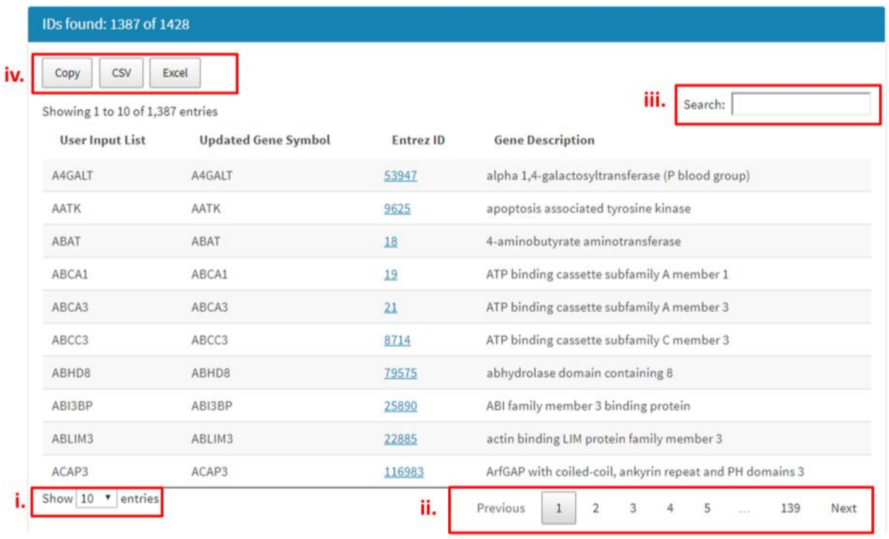

The list of not recognized mRNAs (if any) can be viewed by clicking on the **IDs not found** collapsible panel.

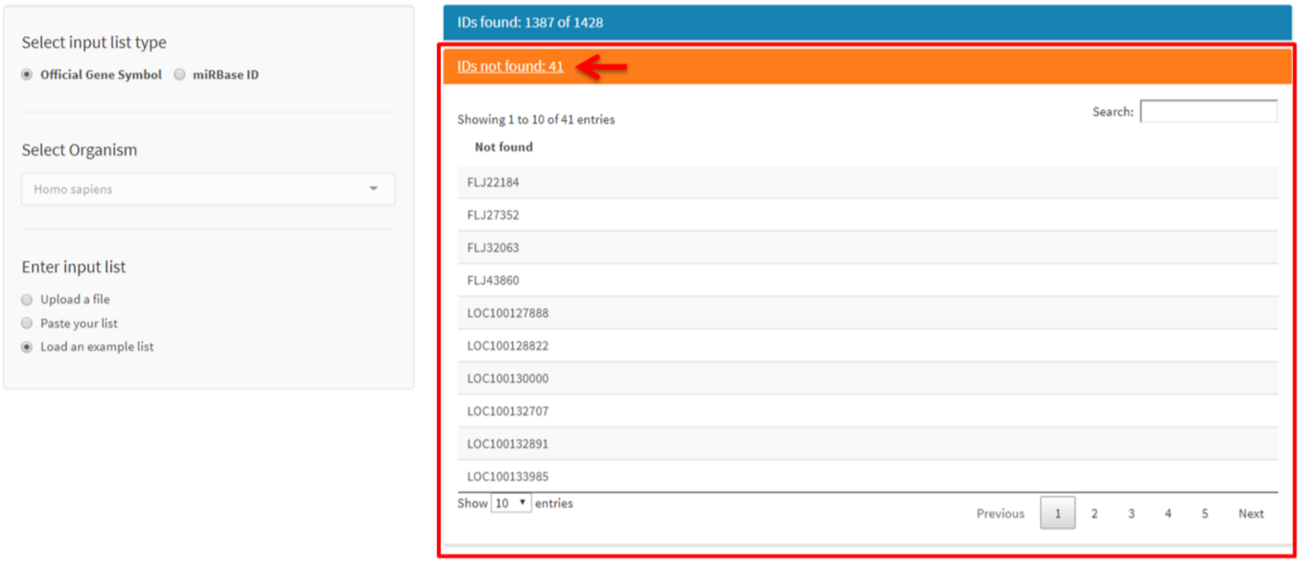

An error message appears either if the input list contains Official Gene Symbols that are not valid, or if the organism is incorrectly selected.

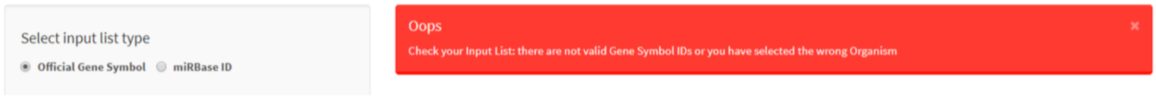

miRNA-target Enrichment link

By clicking on **miRNA-target Enrichment** link, MIENTURNET will perform a statistical analysis for over-representation of miRNA-target interactions in the input gene list.

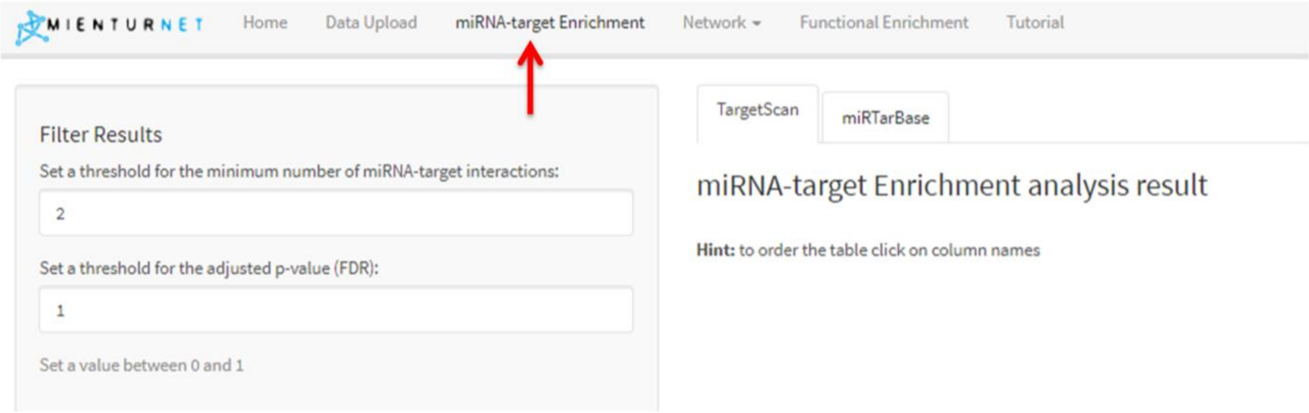

The user has the possibility to perform the enrichment analysis by using a threshold on:

i. the minimum number of miRNA-target interactions (default is 2)
ii. the adjusted p-values (default is 1)

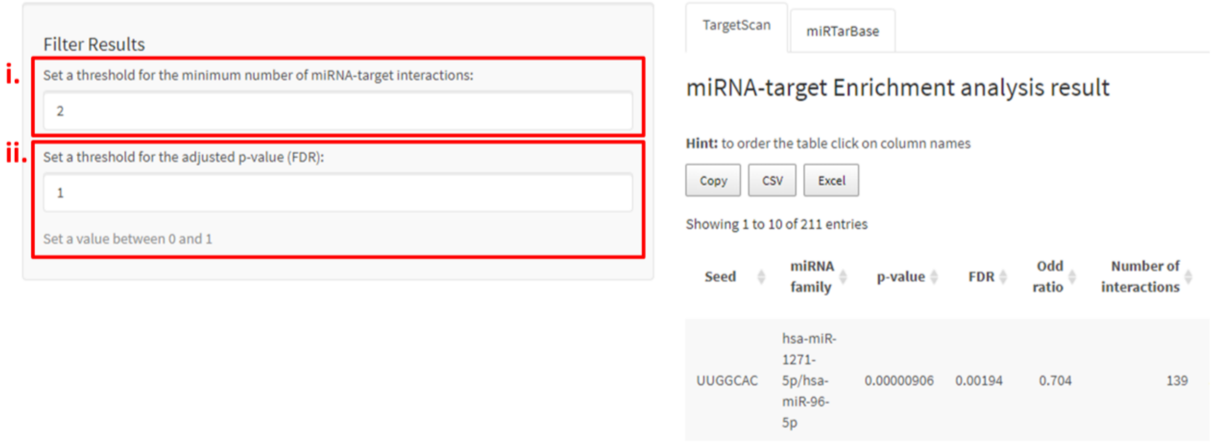

From **miRNA-target Enrichment** page, the user can choose the reference database for the miRNA-target interactions (i.e., TargetScan and/or miRTarBase) to be used to perform the enrichment analysis.

Caveat: regardless of the database choice, MIENTURNET performs the miRNA-target enrichment analysis searching for both predicted and validated interactions. A progress bar in action will appear at the bottom-right of the page as long as both analyses are not completed.

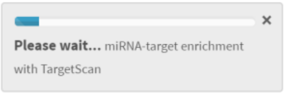

By clicking on **TargetScan** tab, the computationally predicted miRNA-target interactions will be considered.

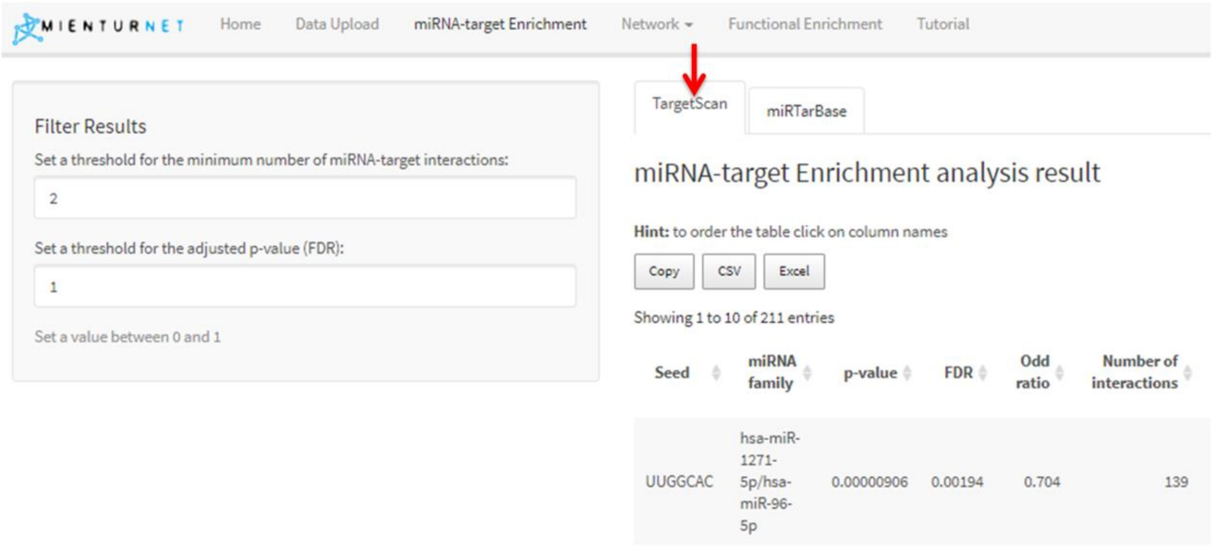

Caveat: TargetScan predicts biological targets of miRNAs by searching for the presence of conserved sites that match the seed region (i.e., the region comprising nucleotides 2-7 at the 5′-end of the mature miRNA sequence) of each miRNA [3]. Note that, a miRNA family is comprised of miRNAs with the same seed sequence. This implies that members of the same miRNA family all share the same predicted targets. As a consequence, in this case, the enrichment analysis was performed considering miRNA families instead of single miRNAs.

By clicking on **miRTarBase** tab, the experimentally validated miRNA-target interactions will be considered.

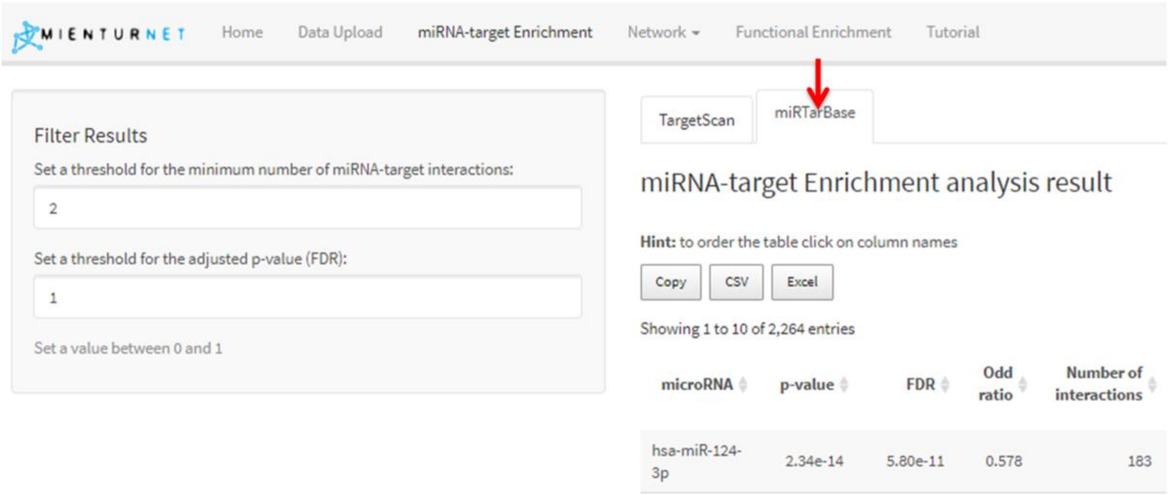

Caveat: miRTarBase collects miRNA-target interactions experimentally validated by reporter assay, western blot, microarrays, and next-generation sequencing experiments. As a consequence, in this case, the enrichment analysis was performed considering miRNAs instead of miRNA families.

For each miRNA in the chosen database (TargetScan or miRTarBase), the hypergeometric test was used to calculate the significance (p-value <0.05) of the enrichment of the input list in targets of that miRNA.

The p-value is computed as

$$p = 1 - \sum_{i=0}^{X-1} \frac{\binom{K}{i}\binom{M-K}{N-i}}{\binom{M}{N}}$$

where M is the dimension of the universe, that is the number of all predicted (validated) miRNA-target interactions encompassed in TargetScan (miRTarBase); K is the number of predicted (validated) miRNA-target interactions encompassed in TargetScan (miRTarBase) for the selected miRNA family (selected miRNA); N is the number of genes of the input gene list recognized by TargetScan (miRTarBase); X is the number of genes for which predicted (validated) miRNA-target interactions, for the selected miRNA family (selected miRNA), exist.

The results of the enrichment analysis are presented as[8]:

i. a bar plot where the Y-axis reports the top ten miRNA families resulted from the enrichment analysis, while the X-axis reports the number of their targets. The color code reflects the FDR value increasing from red to blue. This plot is resizable by grabbing and dragging an active corner at the bottom right of the page.

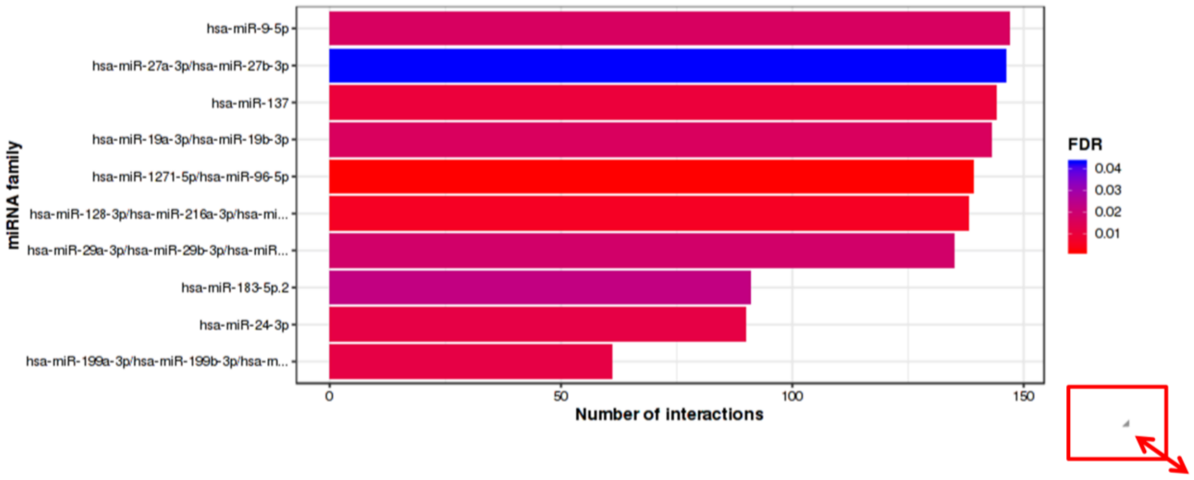

[8] Hereafter, all snapshots refer to TargetScan visualization.

ii. an interactive table where the user has also always the possibility to:

1. visualize the first 10, 25, 50, 100 entries by clicking the drop-down menu at the bottom-left
2. navigate the table trough the navigation controller buttons at the bottom-right
3. search for a miRNA or a Target Gene of interest by typing the name in the search bar at the top-right
4. copy the table to the clipboard, export it in a comma separated format (CSV) or in EXCEL format through the dedicated buttons placed at the top-left

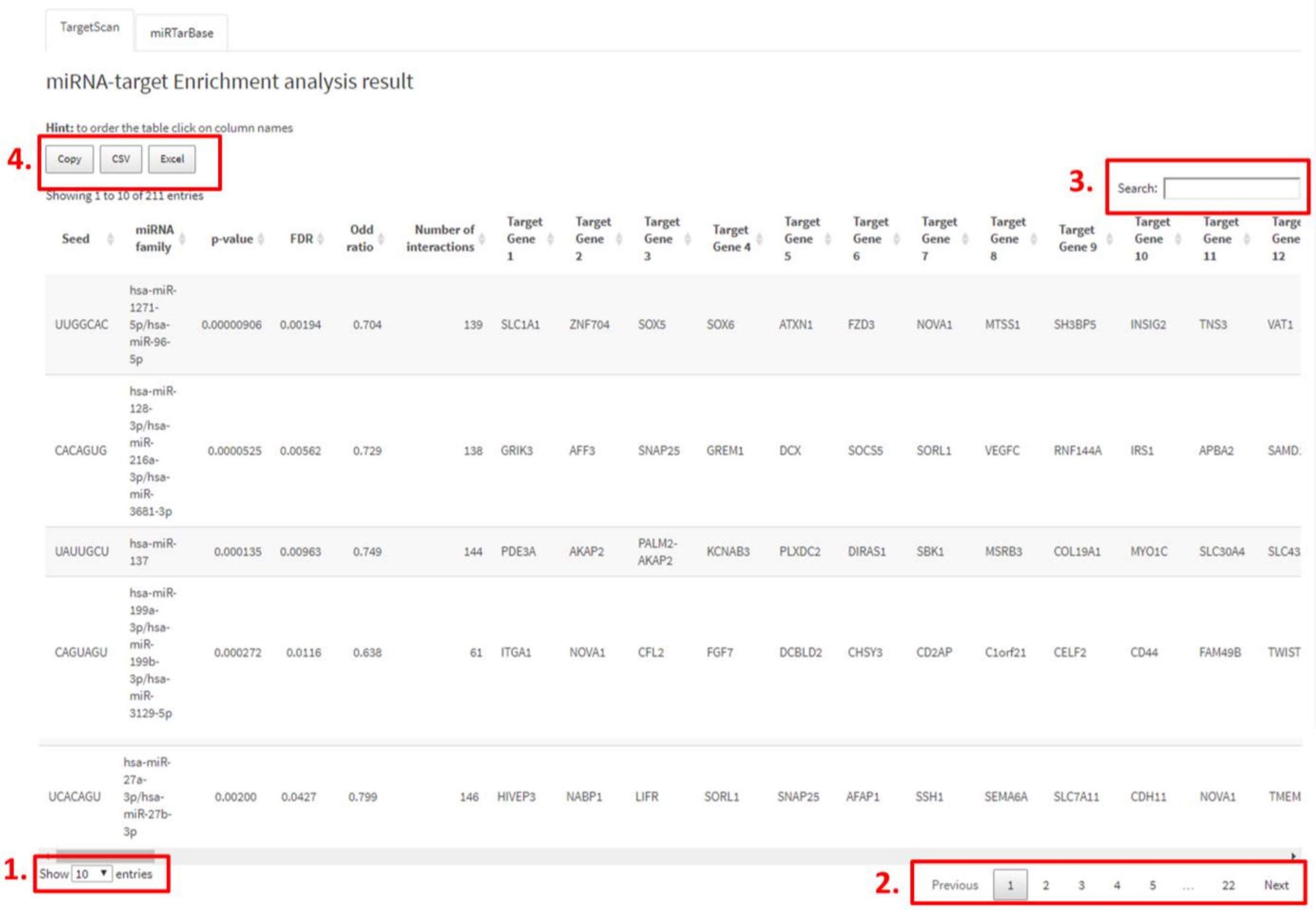

Seed	miRNA family	p-value	FDR	Odd ratio	Number of interactions	Target Gene 1	Target Gene 2	Target Gene 3	Target Gene 4	Target Gene 5	Target Gene 6	Target Gene 7	Target Gene 8	Target Gene 9	Target Gene 10	Target Gene 11	Targe Gene 12
UUGGCAC	hsa-miR-1271-5p/hsa-miR-96-5p	0.00000906	0.00194	0.704	139	SLC1A1	ZNF704	SOX5	SOX6	ATXN1	FZD3	NOVA1	MTSS1	SH3BP5	INSIG2	TNS3	VAT1
CACAGUG	hsa-miR-128-3p/hsa-miR-216a-3p/hsa-miR-3681-3p	0.0000525	0.00562	0.729	138	GRIK3	AFF3	SNAP25	GREM1	DCX	SOCS5	SORL1	VEGFC	RNF144A	IRS1	APBA2	SAMD:
UAUUGCU	hsa-miR-137	0.000135	0.00963	0.749	144	PDE3A	AKAP2	PALM2-AKAP2	KCNAB3	PLXDC2	DIRAS1	SBK1	MSRB3	COL19A1	MYO1C	SLC30A4	SLC43
CAGUAGU	hsa-miR-199a-3p/hsa-miR-199b-3p/hsa-miR-3129-5p	0.000272	0.0116	0.638	61	ITGA1	NOVA1	CFL2	FGF7	DCBLD2	CHSY3	CD2AP	C1orf21	CELF2	CD44	FAM49B	TWIST
UCACAGU	hsa-miR-27a-3p/hsa-miR-27b-3p	0.00200	0.0427	0.799	146	HIVEP3	NABP1	LIFR	SORL1	SNAP25	AFAP1	SSH1	SEMA6A	SLC7A11	CDH11	NOVA1	TMEM

The columns of the table report:

- *Seed:* seeds for each miRNA family
- *miRNA family:* names of all members for each miRNA family
- *p-value:* p-values of the enrichment analysis
- *FDR:* the adjusted p-values obtained by using the Benjamini-Hochberg (False Discovery Rate, FDR) procedure for multiple testing
- *Odd ratio:* ratios calculated as: $\frac{K/M}{X/N}$
- *Number of interactions:* total numbers of interactions for each miRNA family
- Additional extra columns reporting the names of targets included in the gene input list for each miRNA family, named with increasing numerical values

Note that, selecting miRTarBase, the first two columns of the above table will be replaced with a single column with the miRNA names (i.e., miRbase ID).

Network menu

In order to build the network of miRNA-target interactions identified by the enrichment analysis, click on **Network** menu and choose the reference database (i.e., TargetScan and/or miRTarBase) from the drop-down menu.

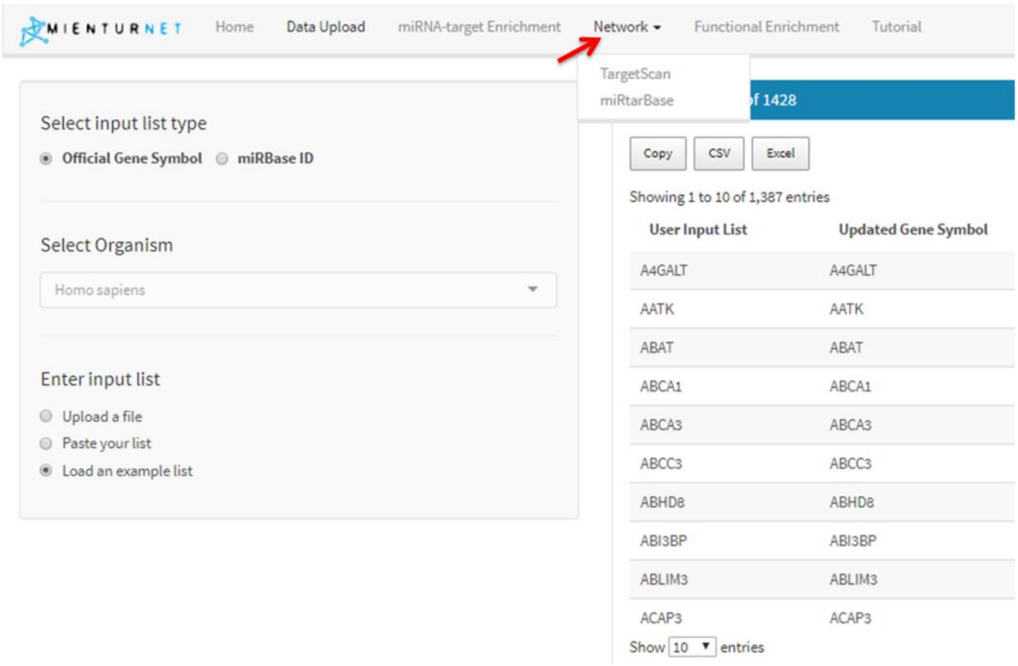

Selecting miRNA-target interactions database and filtering

By choosing **TargetScan**, the results can be filtered by using a threshold on:

i. the minimum number of miRNA-target interactions
ii. the adjusted p-values obtained from the miRNA-target enrichment analysis
iii. the *cumulative weighted context++ score* (CWCS) parameter [4] provided by TargetScan[9]
iv. the *probability of conserved targeting* (P_{CT}) parameter [5] provided by TargetScan

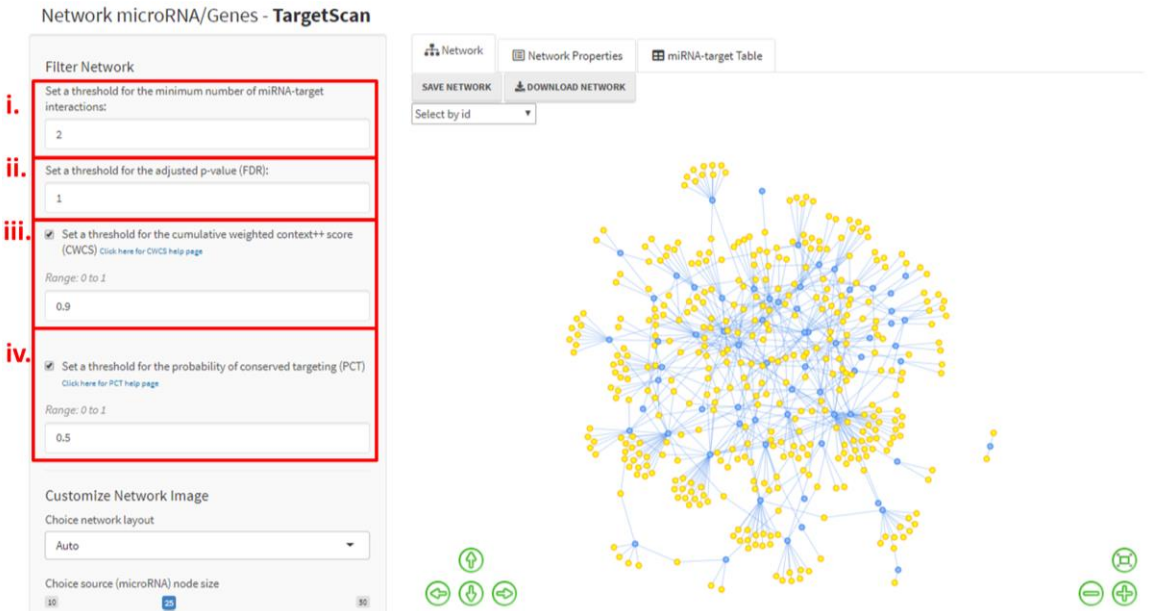

[9] This threshold refers to a percentile of the CWCS distribution.

The user can choose to apply one of these filters, more than one or none. By default, the first two thresholds are set to the values chosen in the **miRNA-target Enrichment** page. The last two parameters, CWCS and P_{CT}, are set by default to 0.9 and 0.5, respectively.

By choosing **miRTarBase**, the results can be filtered according to:

i. a threshold on the minimum number of miRNA-target interactions
ii. a threshold on the adjusted p-values obtained from the miRNA-target enrichment analysis
iii. the type of *Evidence categories* used by miRTarBase to validate the miRNA-target interactions:
    a. ‘Strong’ for considering strong experimental methods (e.g., Luciferase assay, Western)
    b. ‘Weak’ for considering weaker experimental evidence (e.g., CLIP)
    c. ‘Strong and Weak’ for considering both strong and weak experimental methods

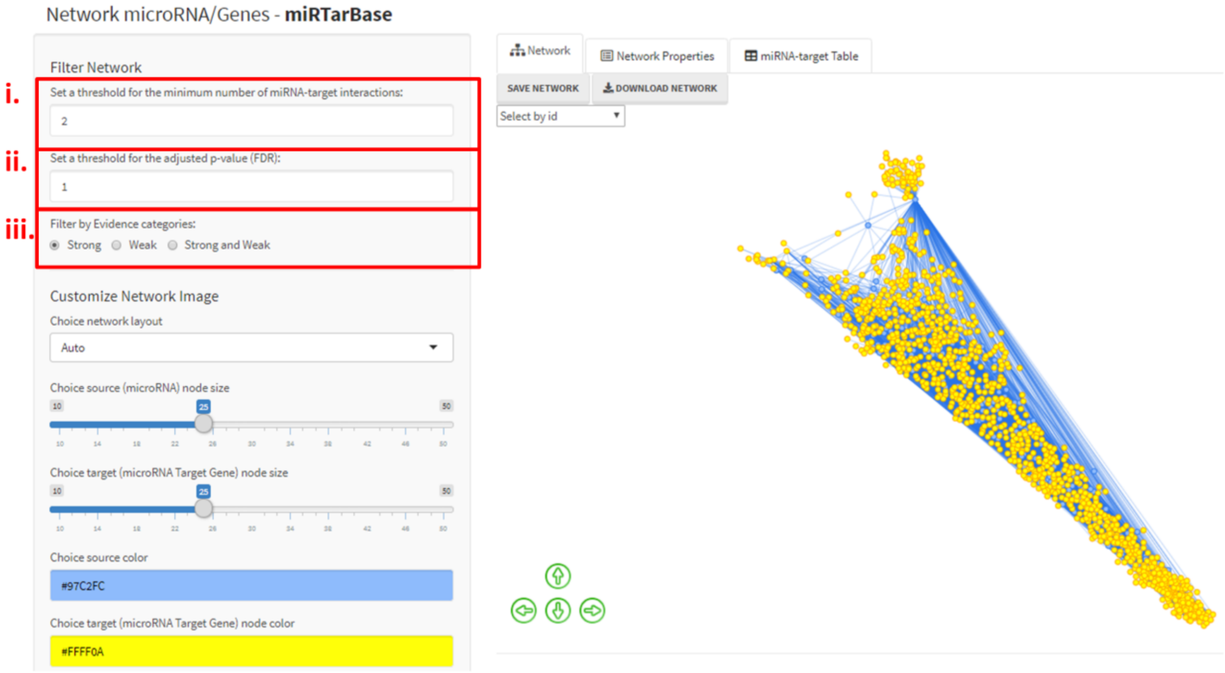

The user can choose to apply one of these filters, more than one or none. As before, by default the first two thresholds are set to the values chosen in the **miRNA-target Enrichment** page. Instead, the *Evidence categories* filter is configured as ‘Strong’.

Caveat: regardless of the database choice, the user that is not interested in the enrichment analysis can skip the previous step (i.e., the user can avoid to click on **miRNA-target Enrichment** link) and jump to the next step of **Network** menu. In this case, MIENTURNET will still perform the enrichment analysis, but will set the default value for the adjusted p-values to 1.

Network visualization and analysis

Both for **TargetScan** and **miRTarBase**, the user can interactively play with the miRNA-interaction network by pressing the following tabs:

1. **Network** tab: it allows to visualize, customize, and download the network in the form of an edgelist (i.e., a 2-column table where each row represents a single edge)
2. **Network properties** tab: it allows to perform a network analysis
3. **miRNA-target Table** tab: it allows to view and download a table where the columns are the miRNAs with the corresponding own targets

In the following, there is a detailed description for each of these tabs[10].

1. Network tab

From **Network** tab, the user can visualize the miRNA-target interactions network:

i. as an image that can be interactively explored and customized
ii. as a 2-column table in the form of an edgelist (i.e., each row represents a miRNA-target interaction).

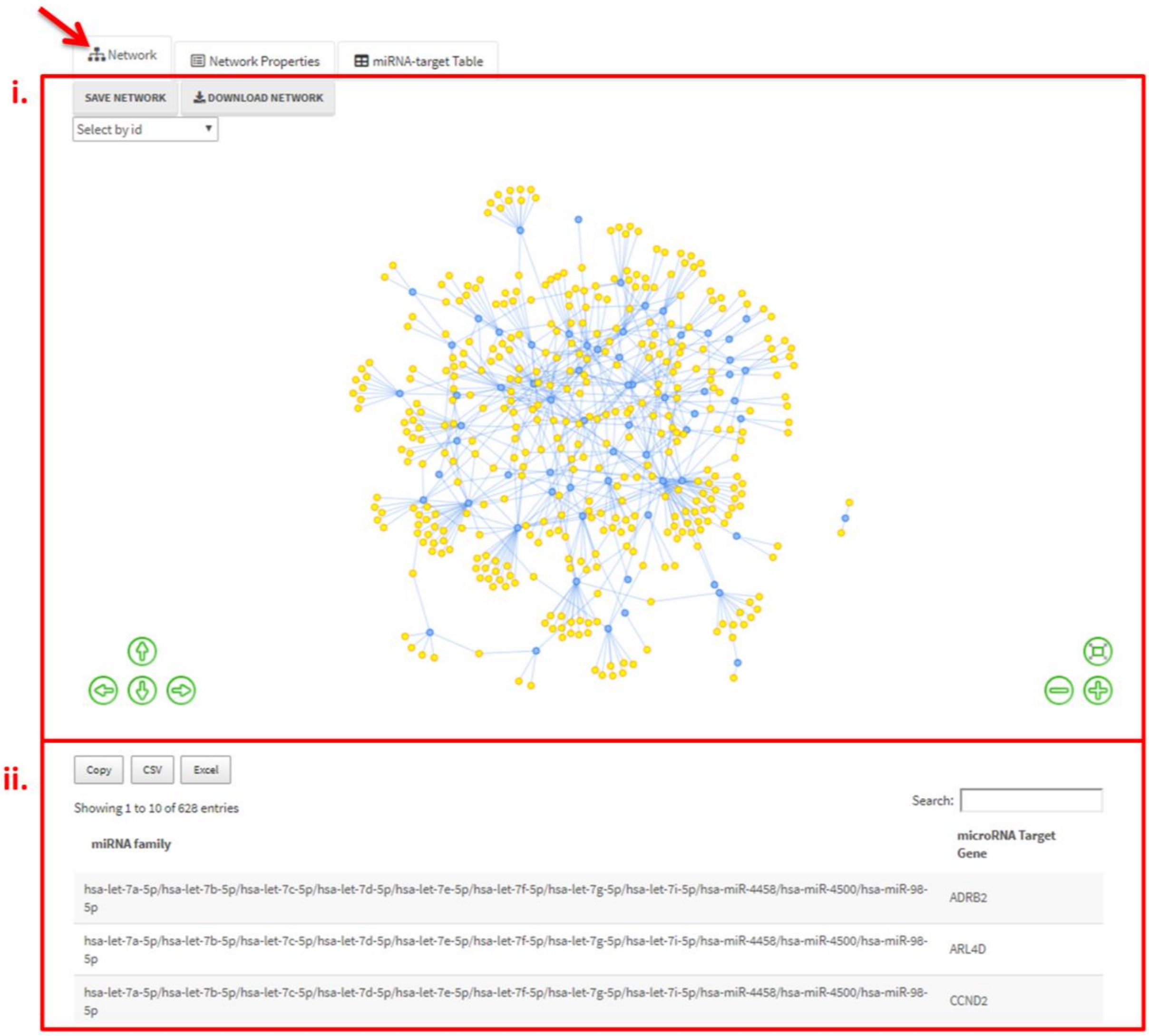

[10] All snapshots refer to TargetScan visualization, where source nodes are the miRNA families to which each miRNA belongs. Otherwise, in the miRTarBase visualization, source nodes are single miRNAs.

By using the *Customize network image* menu on the left, the user may customize:

- the network layout (details about the layout algorithms are available at http://igraph.org)
- the size of source/target nodes
- the shape of source/target nodes
- the color of source/target nodes. By default, source nodes (miRNA families or single miRNAs) are blue circles, while target nodes (mRNAs) are yellow circles.

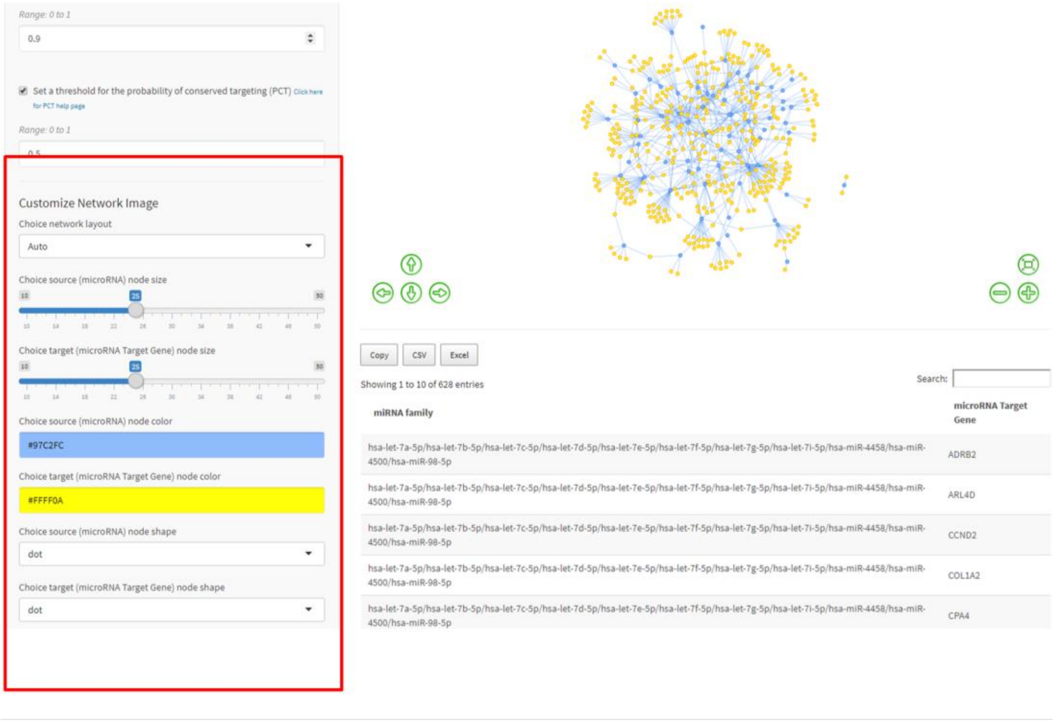

On the network image, the user has also the possibility to:

i. move the network around by using the up-down left-right arrows
ii. zoom in/out the image by using the plus/minus buttons
iii. select a specific node by scrolling down the drop-down menu "Select by id" at the top-left

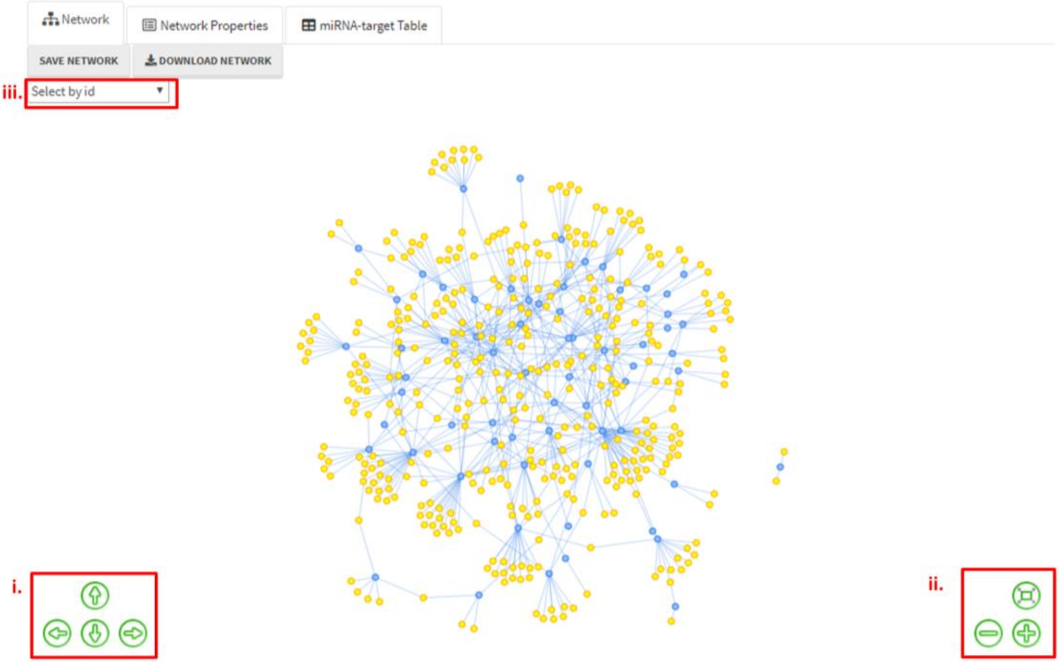

iv. select a specific node by placing the pointer over the node to be selected. Note, the selection will interest the node and its first neighbors.
v. move one or more node by selecting and dragging them with mouse

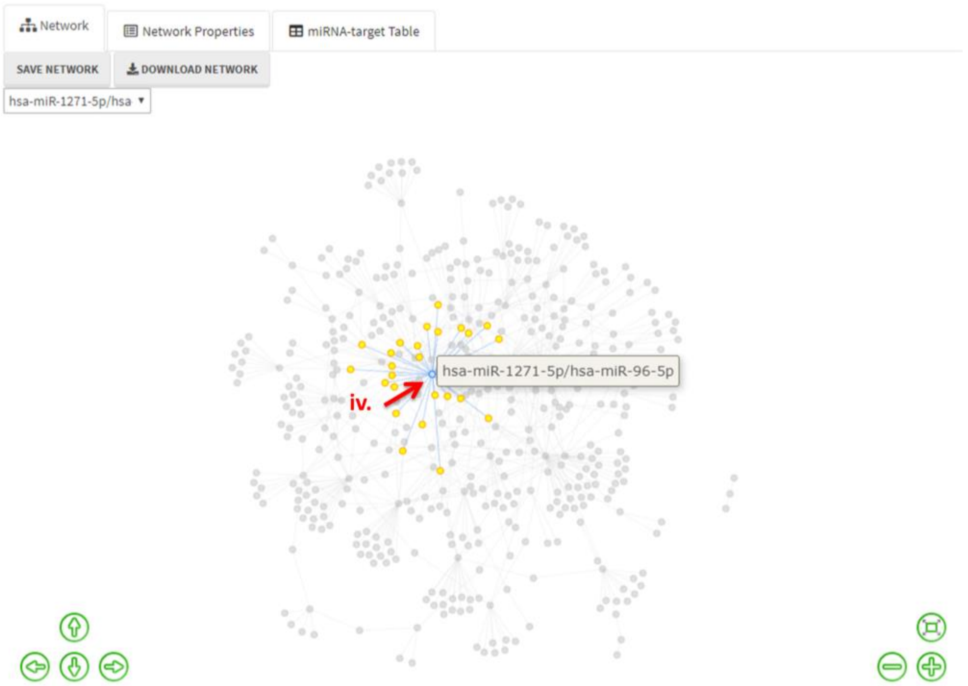

The user can export the network picture as:

i. a dynamic image in HTML format, by clicking first SAVE NETWORK button and then DOWNLOAD NETWORK button at the top-left
ii. a static image in PNG format, by pressing down on the right mouse button (i.e., right-clicking)

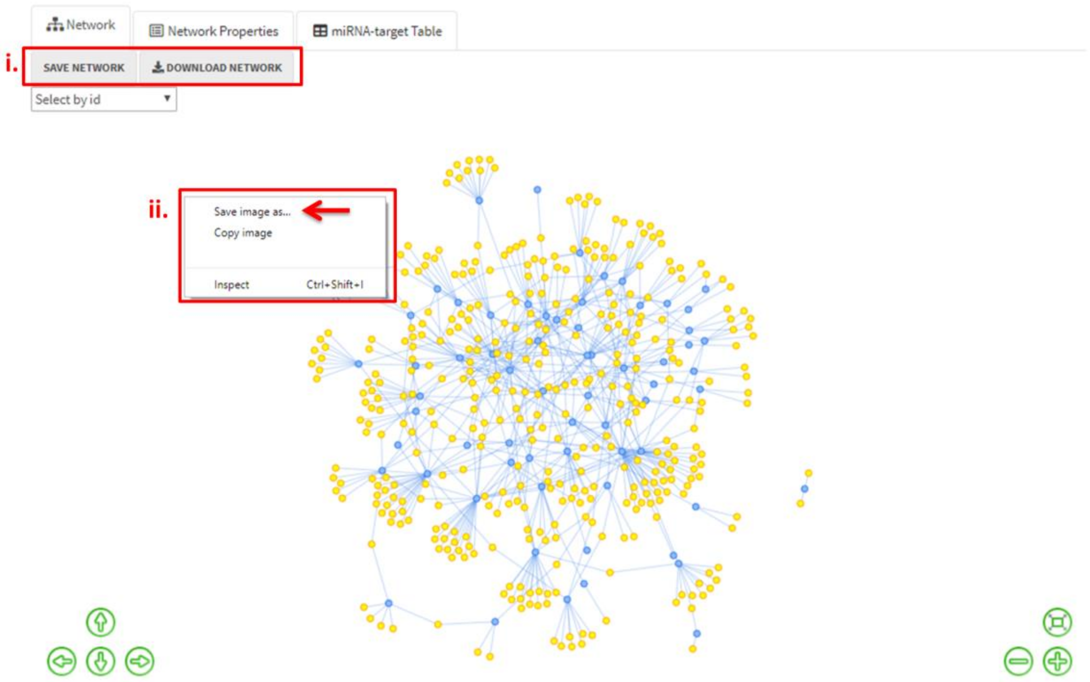

On the network table below the network image, the user has always the possibility to:

i. visualize the first 10, 25, 50, 100 entries by clicking the drop-down menu at the bottom-left
ii. navigate the table trough the navigation controller buttons at the bottom-right
iii. search for a miRNA or a target gene of interest by typing the name in the search bar at the top-right
iv. copy the table to the clipboard, export it in a comma separated format (CSV) or in EXCEL format through the dedicated buttons placed at the top-left.

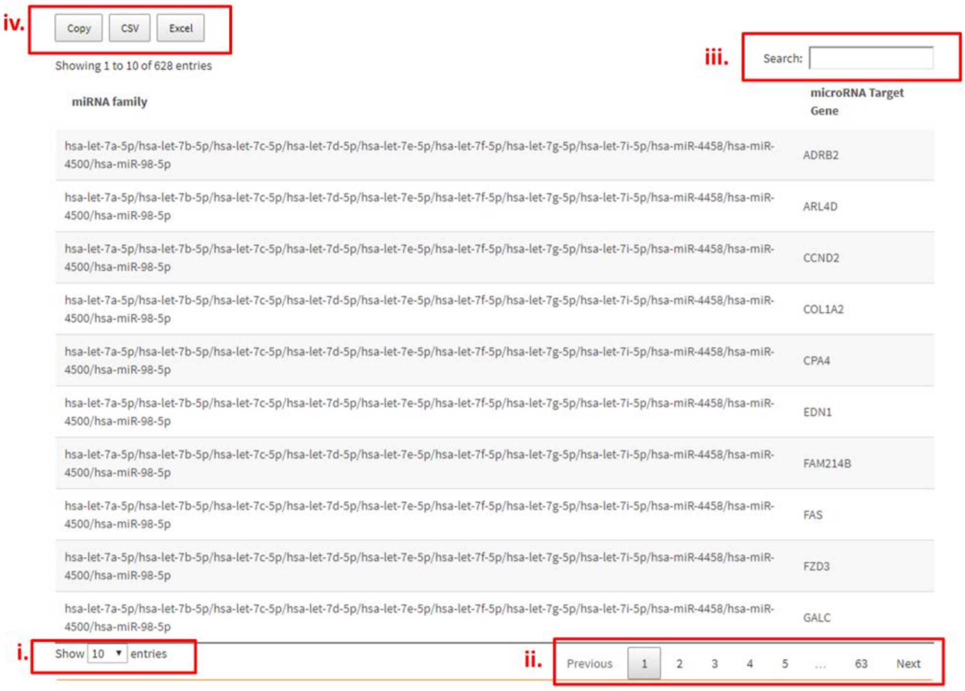

One or more mRNAs in the input list, although recognized in NCBI, could not be in the chosen database (TargetScan or miRTarBase). In this case, the mRNAs not found are listed in the **IDs not found** collapsible panel.

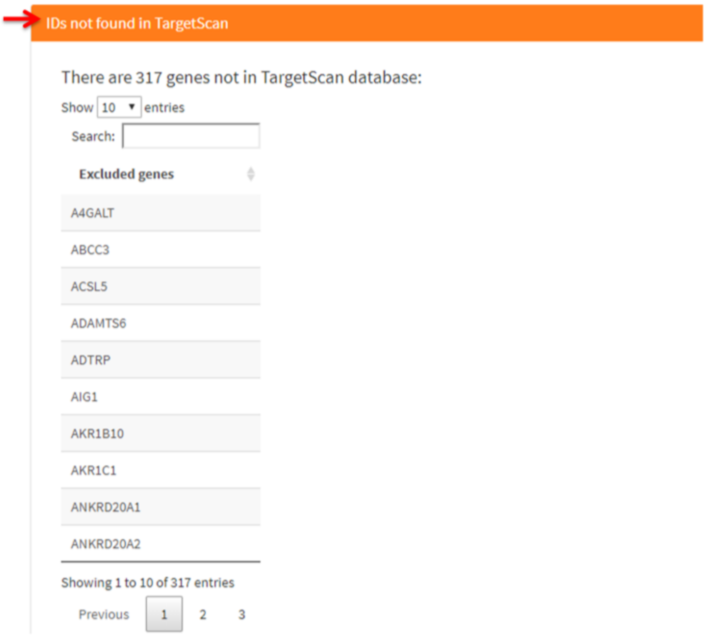

2. Network Properties tab

From **Network Properties** tab, the user can:

i. visualize a table with the topological properties for each node[11], where it is possible to:
   1. sort each column in decreasing or increasing order by clicking on the up-down arrows close to the column names
   2. visualize the first 10, 25, 50, 100 entries by clicking the drop-down menu at the bottom-left
   3. navigate the table trough the navigation controller buttons at the bottom-right
   4. search for a node of interest by typing the name in the search bar at the top-right
   5. copy the table to the clipboard, export it in a comma separated format (CSV) or in EXCEL format through the dedicated buttons placed at the top-left

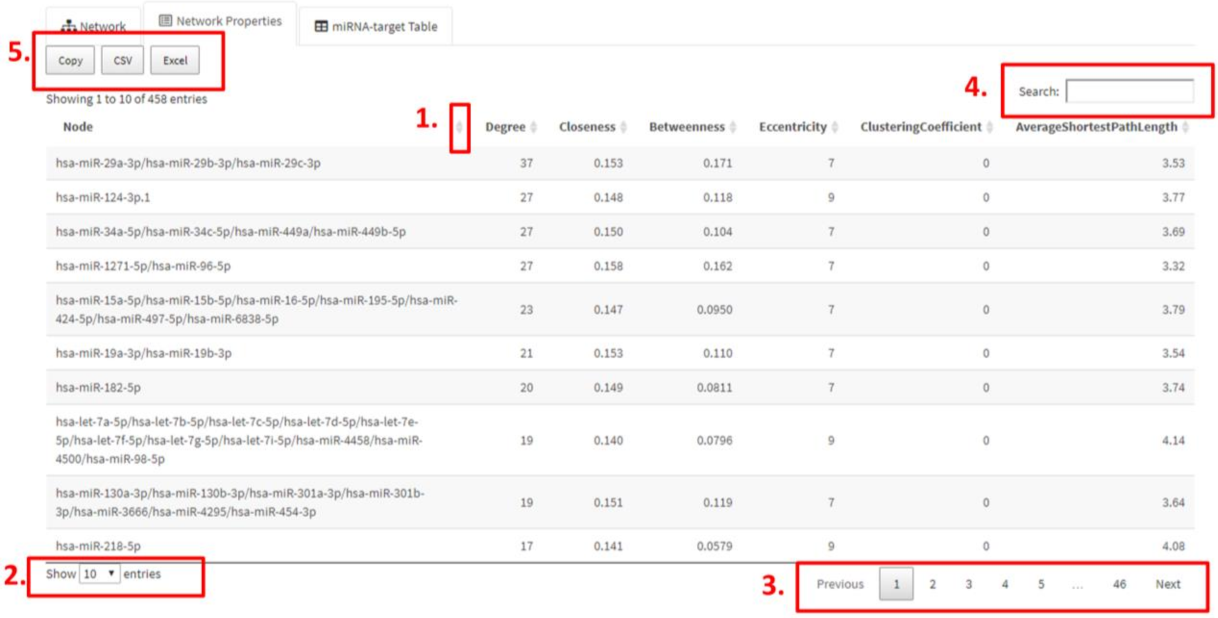

Node	Degree	Closeness	Betweenness	Eccentricity	ClusteringCoefficient	AverageShortestPathLength
hsa-miR-29a-3p/hsa-miR-29b-3p/hsa-miR-29c-3p	37	0.153	0.171	7	0	3.53
hsa-miR-124-3p.1	27	0.148	0.118	9	0	3.77
hsa-miR-34a-5p/hsa-miR-34c-5p/hsa-miR-449a/hsa-miR-449b-5p	27	0.150	0.104	7	0	3.69
hsa-miR-1271-5p/hsa-miR-96-5p	27	0.158	0.162	7	0	3.32
hsa-miR-15a-5p/hsa-miR-15b-5p/hsa-miR-16-5p/hsa-miR-195-5p/hsa-miR-424-5p/hsa-miR-497-5p/hsa-miR-6838-5p	23	0.147	0.0950	7	0	3.79
hsa-miR-19a-3p/hsa-miR-19b-3p	21	0.153	0.110	7	0	3.54
hsa-miR-182-5p	20	0.149	0.0811	7	0	3.74
hsa-let-7a-5p/hsa-let-7b-5p/hsa-let-7c-5p/hsa-let-7d-5p/hsa-let-7e-5p/hsa-let-7f-5p/hsa-let-7g-5p/hsa-let-7i-5p/hsa-miR-4458/hsa-miR-4500/hsa-miR-98-5p	19	0.140	0.0796	9	0	4.14
hsa-miR-130a-3p/hsa-miR-130b-3p/hsa-miR-301a-3p/hsa-miR-301b-3p/hsa-miR-3666/hsa-miR-4295/hsa-miR-454-3p	19	0.151	0.119	7	0	3.64
hsa-miR-218-5p	17	0.141	0.0579	9	0	4.08

The columns of the table report:

- *degree* **-** the number of incoming and outgoing edges of each node
- *betweenness* – the number of shortest paths[12] going through the node
- *average shortest path length* – the average length of the shortest paths between a node and all other nodes in the network
- *eccentricity* – the shortest path distance of a node from the farthest other node in the network
- *clustering coefficient* – measurement the probability that the adjacent nodes of a node are connected. The clustering coefficient is set to 0 for nodes with degree zero and one.
- *closeness* – the inverse of the average length of the shortest paths between a node and all other nodes in the network. Note that the closeness centrality measure is not well-defined for disconnected graphs. If there is no (directed) path between node *i* and *j*, then the total number of nodes is used in the formula instead of the path length.

[11] Details about the network topological properties are available at http://igraph.org
[12] The shortest path between two nodes is the minimum number of consecutive edges connecting them

ii. visualize the following network degree plots:

a. a bar plot of the mRNA degree, where X-axis refers to the first 30 target genes (sorted in a decreasing order according to the degree) and Y-axis refers to their degree (i.e., number of miRNA targeting them)

b. a bar plot of the miRNA degree, where X-axis refers to the first 30 miRNAs (sorted in a decreasing order according to the degree) and Y-axis refers to their degree (i.e., number of their targets)

c. the nodes degree distribution $P(k)$, with k being the degree, by plotting $\log k$ on the X-axis and $\log P(k)$ on the Y-axis. Then, the method of least squares is used to find the best-fitting curve $\log P \sim \alpha \log k$, where α is the constant to estimate. The sum of the squared residuals R^2 (i.e., the sum of the vertical deviations from the best-fitting line) is reported together with the estimation of the coefficient α.

All plots can be exported as a compressed .zip file by clicking on SAVE DEGREE PLOTS button.

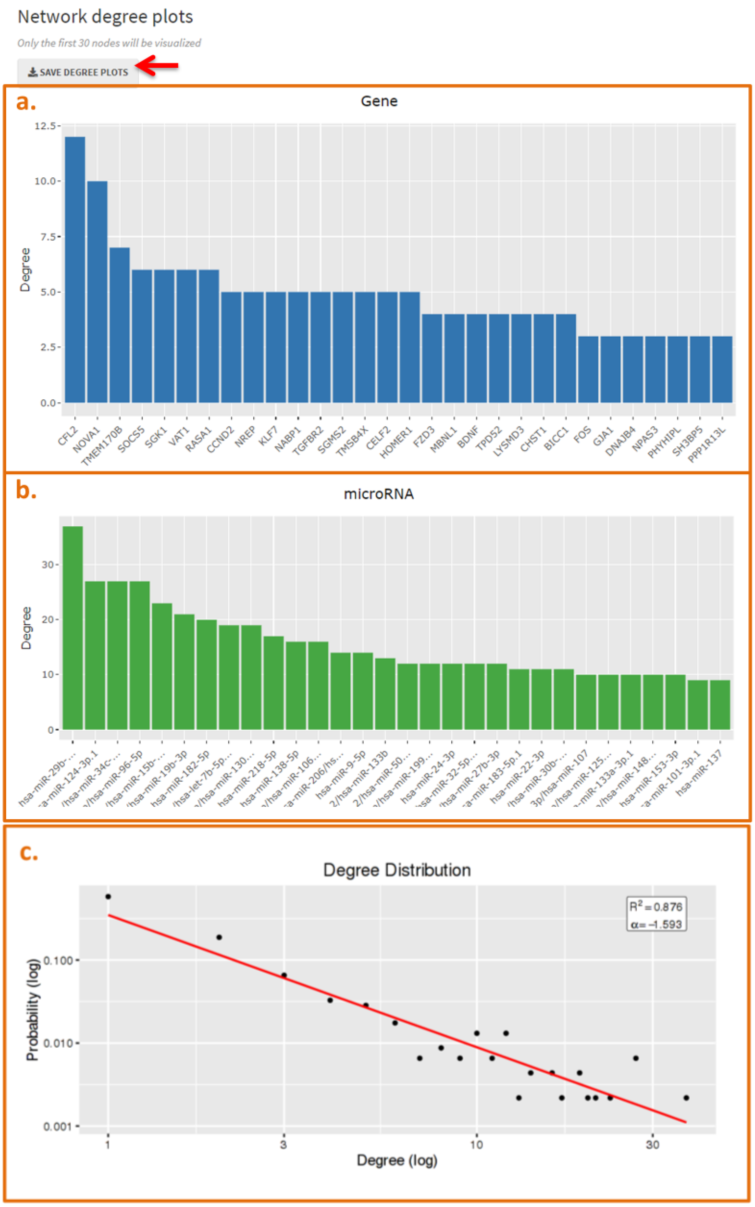

3. miRNA-target Table tab

From **miRNA-target Table** tab, the user can visualize an interactive table where the columns are the mRNAs of the input list found in the selected database (TargetScan or miRTarBase) with the corresponding miRNAs targeting them. The columns of the table are sorted in a decreasing order according to the number of miRNAs targeting each gene. The user has always the possibility to:

i. visualize the first 10, 25, 50, 100 entries by clicking the drop-down menu at the top-left
ii. navigate the table trough the navigation controller buttons at bottom-right
iii. search for a miRNA or a Target Gene of interest by typing the name in the search bar at the top-right
iv. copy the table to the clipboard, export it in a comma separated format (CSV) or in EXCEL format through the dedicated buttons placed at the top-left.

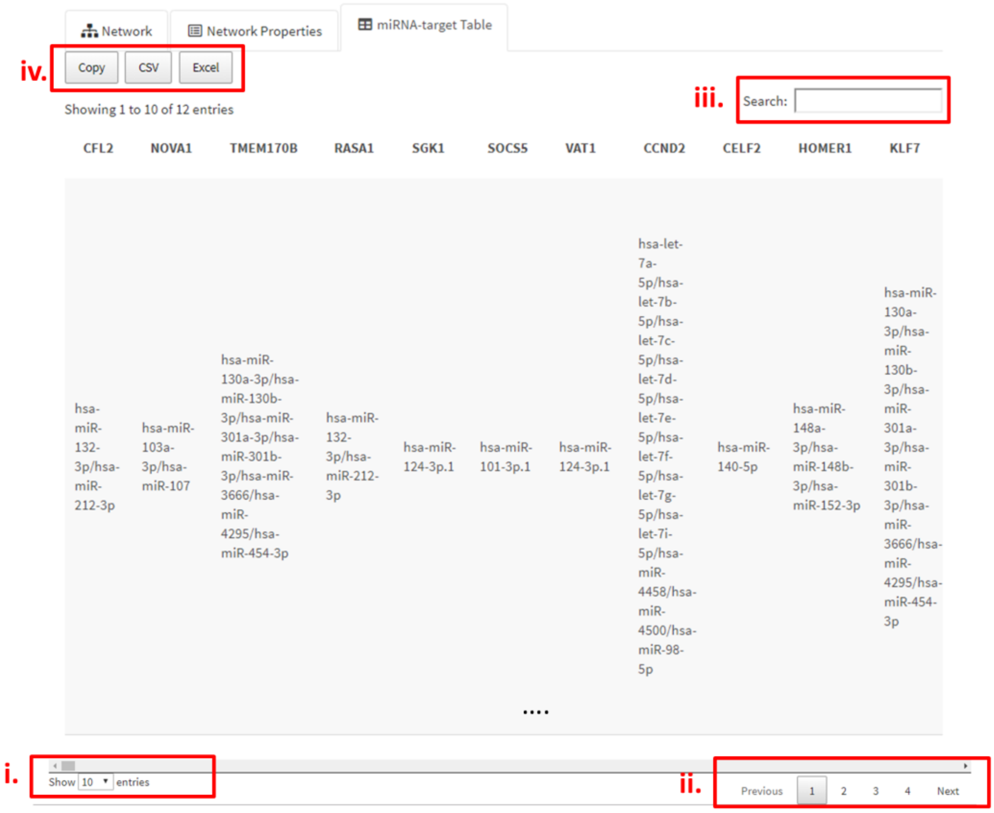

Functional Enrichment link

In order to perform the functional enrichment analysis of target genes of selected miRNAs, click on **Functional Enrichment** link and choose TargetScan or miRTarBase [13].

Currently, MIENTURNET allows to query the following annotation databases: KEGG, REACTOME, WikiPathways, Disease Ontology. Note that the last one will be available only when *Homo sapiens* is the selected organism.

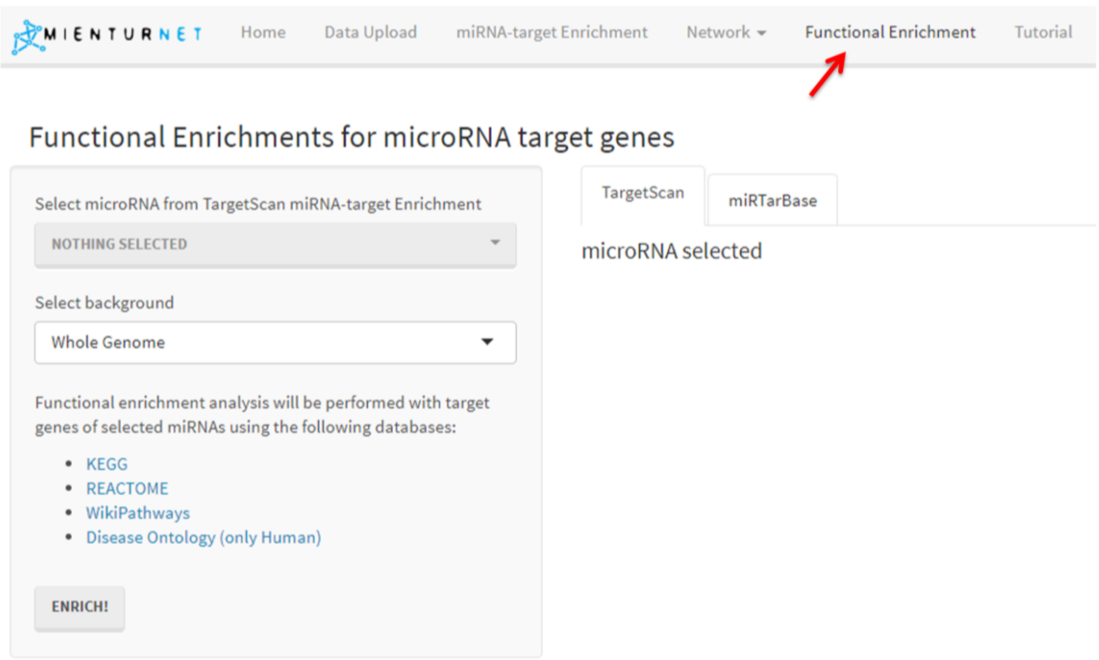

Firstly, the user can select one or more miRNA families (or single miRNA in the case of miRTarBase) by clicking on each item or by typing the name in the search box. In this example, the first three miRNA families have been selected.

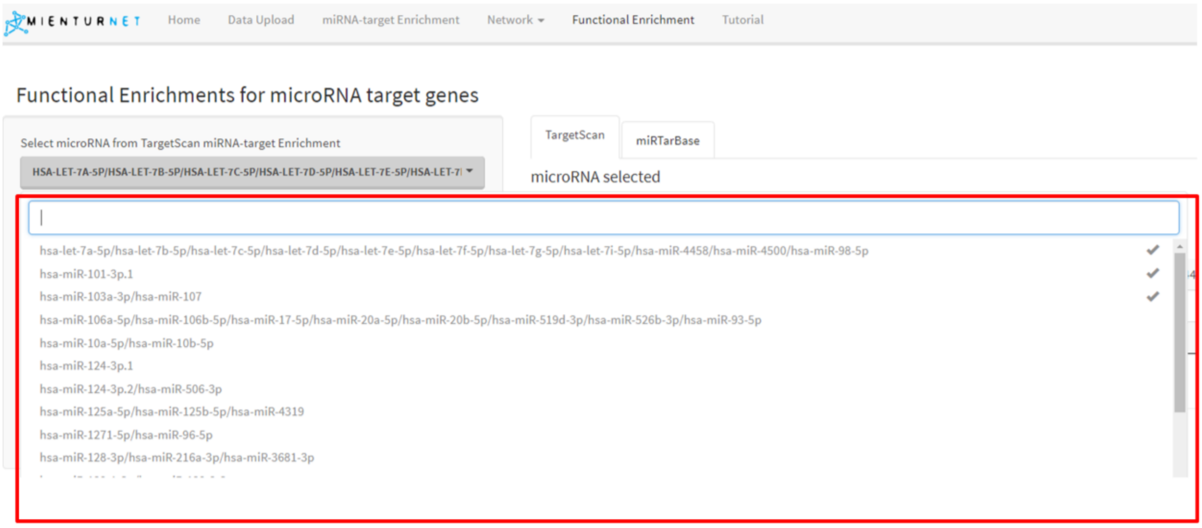

[13] All snapshots refer to TargetScan visualization, where for each selected miRNA the family to which it belongs will be reported. Otherwise, in the miRTarBase visualization, the selected miRNAs will be considered.

The selected miRNA families will appear in a table on the right along with the number of their interactions.

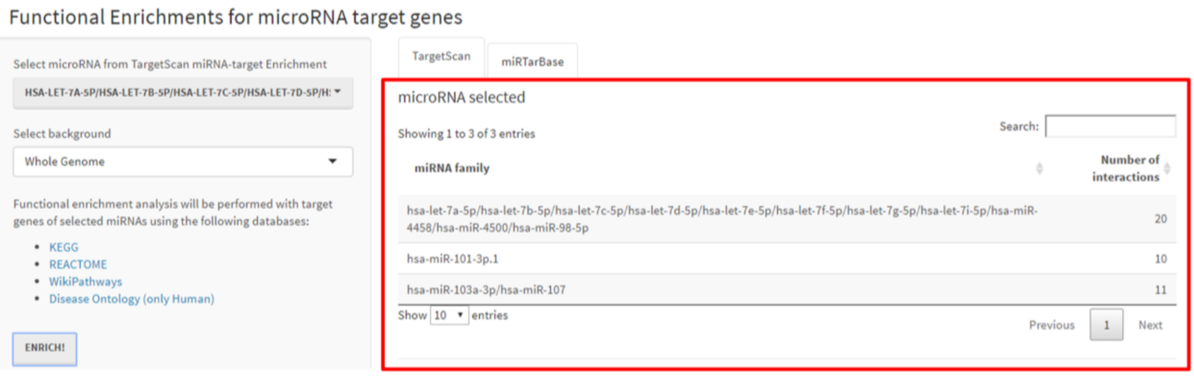

Secondly, the user can select the background set. Currently, the choice is between: “Whole Genome” (i.e., all annotated genes in the annotation database will be used as reference set), or “Your input list of genes” (i.e., the user’s input list of gens will be used as reference set).

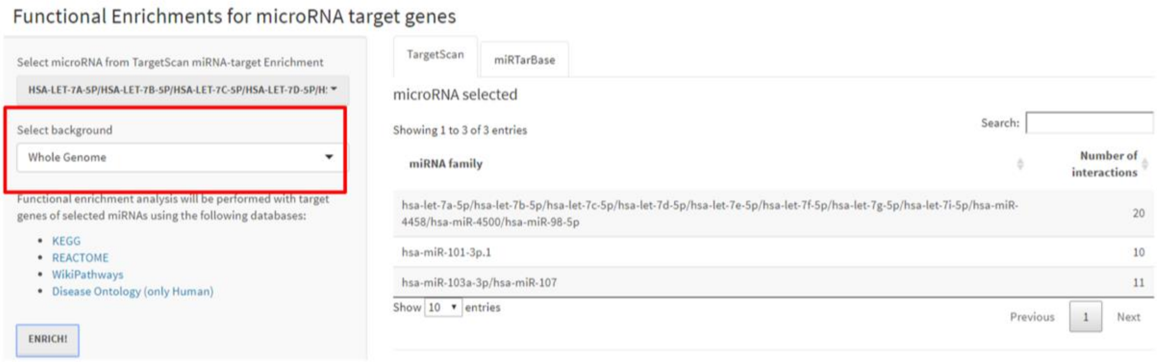

To start the analysis, click the ENRICH button.

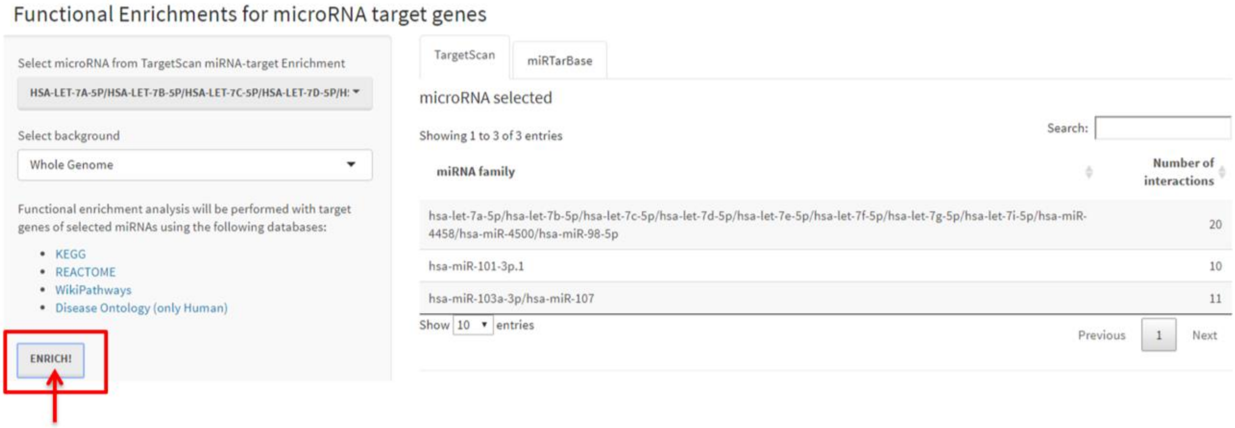

Caveat: The function enrichment analysis will regard the targets of each selected miRNA, separately. The user could see a progress bar in action at the bottom-right of the page.

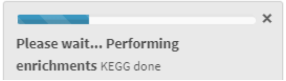

For each annotation database, the results of the functional enrichment analysis are presented as[14]:

3. an interactive table reporting: the miRNA name, the category name of the annotation database (e.g., KEGG ID), the category description, the p-value and the adjusted p-value (FDR), the list of target genes, the number of targets genes.
4. a dot plot, where the Y-axis reports the annotation categories (e.g., KEGG pathways) and the X-axis reports the miRNA family with the number of recognized targets in round brackets. The colors of the dots represent the adjusted p-values (FDR), whereas the size of the dots represents gene ratio (i.e., the number of miRNA targets found annotated in each category over the number of total genes annotated in that category). All the plots are resizable by grabbing and dragging an active corner at the bottom right of the page.

All the results can be saved by clicking on SAVE ALL RESULTS AND FIGURES button.

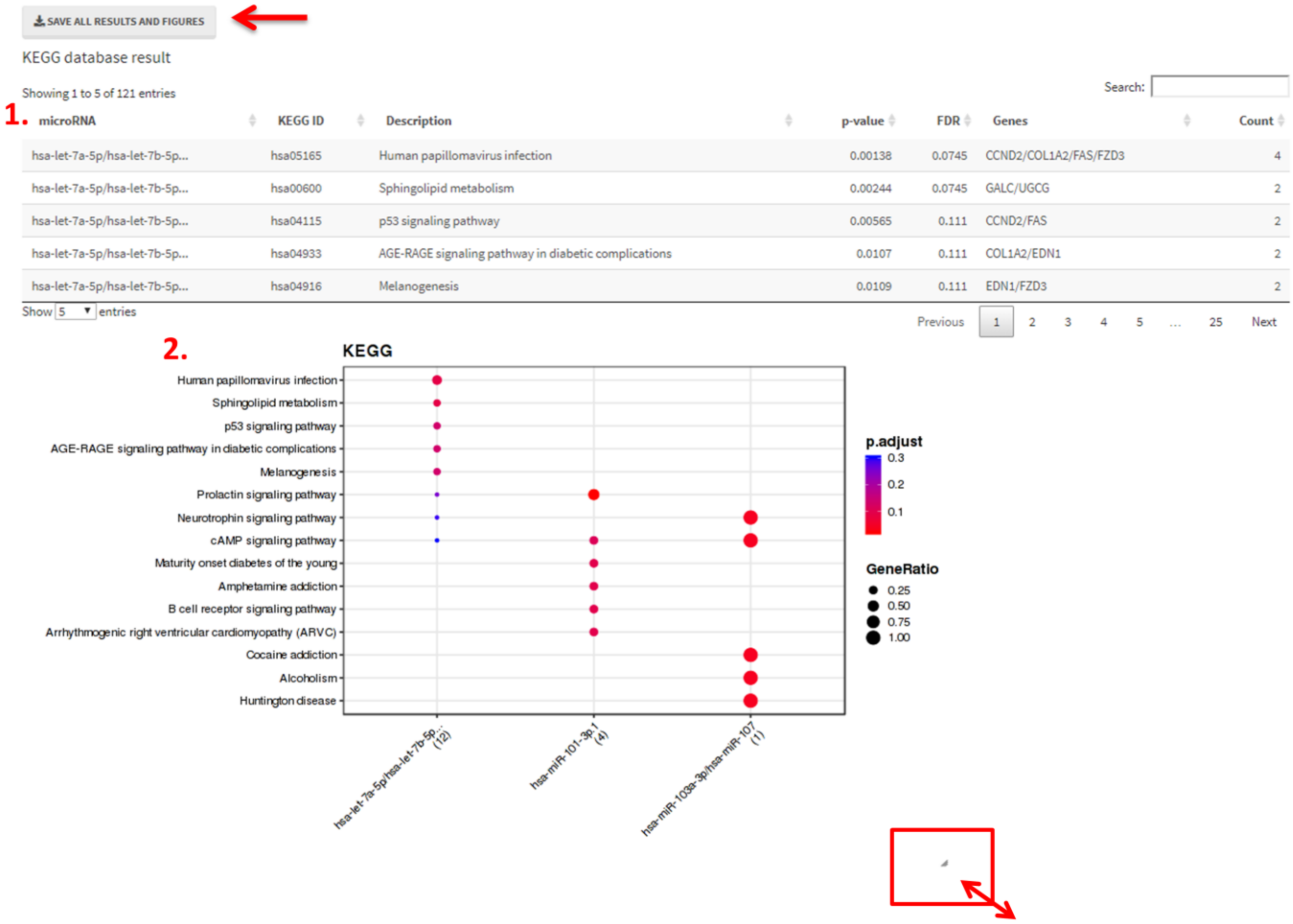

[14] The results presented here refer to the KEGG enrichment analysis.

References

[1] B. P. Lewis, I. Shih, M. W. Jones-Rhoades, D. P. Bartel, and C. B. Burge, “Prediction of mammalian microRNA targets,” *Cell*, vol. 115, no. 7, pp. 787–798, 2003.

[2] C.-H. Chou *et al.*, “miRTarBase 2016: updates to the experimentally validated miRNA-target interactions database,” *Nucleic Acids Res.*, vol. 44, no. D1, pp. D239–D247, 2016.

[3] B. P. Lewis, C. B. Burge, and D. P. Bartel, “Conserved seed pairing, often flanked by adenosines, indicates that thousands of human genes are microRNA targets,” *Cell*, vol. 120, no. 1, pp. 15–20, Jan. 2005.

[4] V. Agarwal, G. W. Bell, J.-W. Nam, and D. P. Bartel, “Predicting effective microRNA target sites in mammalian mRNAs.,” *Elife*, vol. 4, 2015.

[5] R. C. Friedman, K. K.-H. Farh, C. B. Burge, and D. P. Bartel, “Most mammalian mRNAs are conserved targets of microRNAs,” *Genome Res.*, vol. 19, no. 1, pp. 92–105, Jan. 2009.

[6] G. Fiscon, F. Conte, V. Licursi, S. Nasi, and P. Paci, “Computational identification of specific genes for glioblastoma stem-like cells identity,” *Sci. Rep.*, vol. 8, no. 1, p. 7769, May 2018.
